# A hybrid multifunctional physicochemical sensor suite for continuous monitoring of crop health

**DOI:** 10.1038/s41598-023-37041-z

**Published:** 2023-06-17

**Authors:** Nafize Ishtiaque Hossain, Shawana Tabassum

**Affiliations:** The University of Texas at Tyler, Tyler, TX 75799 USA

**Keywords:** Biotechnology, Plant sciences, Engineering

## Abstract

This work reports a first-of-its-kind hybrid wearable physicochemical sensor suite that we call PlantFit for simultaneous measurement of two key phytohormones, salicylic acid, and ethylene, along with vapor pressure deficit and radial growth of stem in live plants. The sensors are developed using a low-cost and roll-to-roll screen printing technology. A single integrated flexible patch that contains temperature, humidity, salicylic acid, and ethylene sensors, is installed on the leaves of live plants. The strain sensor with in-built pressure correction capability is wrapped around the plant stem to provide pressure-compensated stem diameter measurements. The sensors provide real-time information on plant health under different amounts of water stress conditions. The sensor suite is installed on bell pepper plants for 40 days and measurements of salicylic acid, ethylene, temperature, humidity, and stem diameter are recorded daily. In addition, sensors are installed on different parts of the same plant to investigate the spatiotemporal dynamics of water transport and phytohormone responses. Subsequent correlation and principal component analyses demonstrate the strong association between hormone levels, vapor pressure deficit, and water transport in the plant. Our findings suggest that the mass deployment of PlantFit in agricultural settings will aid growers in detecting water stress/deficiency early and in implementing early intervention measures to reduce stress-induced yield decline.

## Introduction

The world population is estimated to reach 11.2 billion by 2100, while the total cultivable land will not change significantly^1^. The most promising strategy to produce enough food for humans and livestock in the future is to make farms more efficient, profitable, and sustainable in their use of nonrenewable resources. Plants are subjected to biotic (such as microbes, herbivores, invasive plants, and pests attack) and abiotic (drought, flood, salinity, extreme heat/cold, and nutrient deficiencies) stresses throughout their lifecycle^2,3^. These environmental stresses induce time-dependent biochemical changes, including reduced transpiration and systemic oxidative stress. As a result, there occurs a progressive variation in the levels of phytohormones, which circulate throughout the plant via the xylem and phloem, and thus, the levels of phytohormones can serve as early signals of plant stress^4^. The phytohormones are considered key stress signaling molecules in plants. The primary signal molecules that are produced as a plant’s first response to environmental stresses include non-volatile phytohormones such as Salicylic acid (SA), Jasmonic acid (JA), Abscisic acid (ABA), and Indole-3-acetic acid (IAA), and volatile organic compounds including ethylene and terpenoids^5–7^. SA and JA are the two primary phytohormones released during systemic acquired resistance (a mechanism of protection against a wide variety of stresses), while ethylene and other phytohormones modulate the overall plant response^8–11^. Alternating levels of SA, JA, and ABA have been proven to be an indicator of drought, salt, and temperature stresses in plants^12–16^. Likewise, plants release ethylene and terpenoids under abiotic stresses such as drought, salinity, and temperature variations, and biotic stresses such as pests, herbivores, or microbe attacks^17^. Ethylene also regulates fruit ripening and the development and senescence processes in plants^18^. Therefore, accurate and timely measurements of SA, JA, ABA, IAA, and ethylene would aid the producers and scientists in early diagnosing crop stresses before visible symptoms appear and optimizing the resources to minimize stress-induced growth and yield declines in plants.

In addition to phytohormones, stomata (adjustable pores beneath the crop leaves) regulate photosynthesis and other internal processes in plants by controlling gas exchange with the ambient. For instance, transpiration facilitates the release of water vapor by controlling the carbon dioxide intake, oxygen release, and utilization of nutrients^19^. Particularly, transpiration is directly related to the vapor pressure deficit (VPD)^20^, which depends on both temperature as well as relative humidity (RH) levels of the ambient and leaf surfaces. Higher VPD results from significantly higher transpiration compared to the translocation of water from the soil to the leaf ^21,22^. As a result, the plant is under water stress and requires more water to utilize the peripheral CO_2_^23^. In contrast, a lower VPD value indicates vapor saturation on the leaf surface, which can be a driving factor for fungal infection on leaves^24^. Thus, the VPD is an effective measure of the transportation of water and nutrients from the soil to the leaves. Similar to phytohormone levels, VPD levels are regulated by temperature, humidity, duration of sunlight exposure, and soil water content^25–28^. Therefore, real-time measurements of VPD are crucial to plant growth monitoring and will aid in managing the rate at which the plants transpire.

The traditional investigation of plant signaling does not involve continuous and real-time measurements. As a result, the time signatures carried by the chemical species cannot be captured. A recent article published in Nature Reviews Chemistry has reviewed the recent advances in sensing technology for monitoring signaling molecules in plants^29^. This article identified the lack of studies with live whole plants as one of the major challenges in commercializing the sensors. To date, several temperature and humidity sensors have been reported in the literature^30–34^. However, a limited number of research studies have been conducted on leaf-scale measurements. Some notable leaf sensors include a flexible integrated sensor for light intensity, temperature, and humidity monitoring^35^, a microfluidic-printed electromechanical sensor for monitoring stomatal dynamics^19^, an integrated on-leaf temperature and humidity sensor for measuring vapor pressure deficit^36^, a leaf surface relative humidity sensor for tracking water movement dynamics inside plants^37^, and leaf VOC (2-hexenal(E)) sensor for plant disease diagnostics^38^. However, the aforementioned techniques do not provide real-time measurements of defense responses in plants. Traditional technologies that measure the non-volatile phytohormones such as SA, JA, ABA, and IAA include capillary electrophoresis (CE)^39^, high-performance liquid chromatography (HPLC)^40,41^, and nuclear magnetic resonance (NMR) spectroscopy^42^, while gas chromatography-mass spectroscopy and fluorescence-based techniques^43^ are used extensively for gaseous molecule detection. However, these established chemical detection technologies do not provide minimally invasive, in-situ, and high-frequency continuous plant measurements in the field because these methods are discrete, disruptive, time-intensive, and often not effective until the plants show physical signs of stress. They often involve expensive and specialized instruments. Moreover, the quality of samples degrades owing to their transportation from the field to the lab for analysis. Therefore, a substantial knowledge gap exists in understanding the real-time dynamics of plant defense responses under environmental stressors. An integrated crop-wearable sensor suite will be pivotal in plant science research for detecting crop stresses early by enabling real-time measurements of the dynamics of water uptake and phytohormone gradients.

Although flexible electrochemical sensors have been reported in the literature for monitoring phytohormones^44–47^, the research field is still in its infancy and little to no research has been conducted on developing an integrated hybrid sensing system for real-time quantification of volatile and liquid phytohormones and VPD levels along with the radial growth of stem for early diagnosis of crop stress. This work reports the first-of-its-kind integrated, flexible, and multiparametric sensor suite that enables continuous and non-destructive monitoring of a plant’s physiology with spatiotemporal fidelity. The sensor suite is comprised of leaf temperature and RH sensors for measuring VPD levels, a strain sensor for measuring the radial growth of stem with onboard pressure correction capability, and an electrochemistry-based multiplexed sensor for measuring SA and ethylene levels, to perform a comprehensive assessment of crop health in real-time. In an agricultural field, sensor performance can be affected by undesired pests, insects, bees, animals, or human intervention^48,49^. Particularly, when pests/insects sit on the strain sensor, the added weight (and hence pressure) incurs a strain variation, thereby resulting in a false positive reading. Therefore, a pressure sensor was incorporated with the strain sensor to correct the strain measurements resulting from unwanted weight/pressure variations. Our experiments demonstrate negligible variations in the SA, ethylene, temperature, and RH sensor data under repeated application of strain. Hence, a separate pressure sensor was not required to correct these sensor measurements and the onboard pressure sensor was integrated with the strain sensor only. Real-time measurements of leaf temperature, RH, SA, ethylene, and stem diameter were recorded from plants for 40 days. All the measurements were wirelessly transmitted to the cloud through an Internet-of-Things (IoT) platform interfaced with the flexible sensors. A noticeable correlation was observed between the measured parameters and periodic water stress conditions. In addition, the cross-correlation analysis elucidated a significant association between the SA, ethylene, and VPD levels, a finding that has not been reported previously. These findings suggest a promising link between hormone responses, plant transpiration, and soil water content. Furthermore, a pattern recognition algorithm, such as principal component analysis (PCA) was used to extract selective responses of the sensors for different stress and growth stages of the plants. The pilot experiments conducted with our integrated sensor suite demonstrate its potential to uncover molecular processes and their interactions underlying a stress response. Time-resolved, continuous measurement of chemical signals inside plants will also aid the implementation of early intervention measures to reduce stress-induced yield decline. The key innovations of this work are listed below:

- An IoT-enabled integrated sensor suite that is wearable to plants and provides in situ and real-time measurements of four key plant health parameters, VPD, SA, ethylene, and radial growth of stem
- Production of the sensor suite through a cost-effective screen-printing procedure
- A comprehensive cross-correlation study to establish the association between hormone levels and VPD
- PCA-enabled selective identification of sensor responses to stress and growth stages

## Materials and methods

The details of the sensor fabrication, surface functionalization, working mechanism, and spectroscopic and microscopic analyses are explained in the Supporting Information (Sections S1–S3, Figs. S1–S8, and Tables S1–S2).

### Design and architecture of multifunctional plant wearable sensor

**Figure 1:**
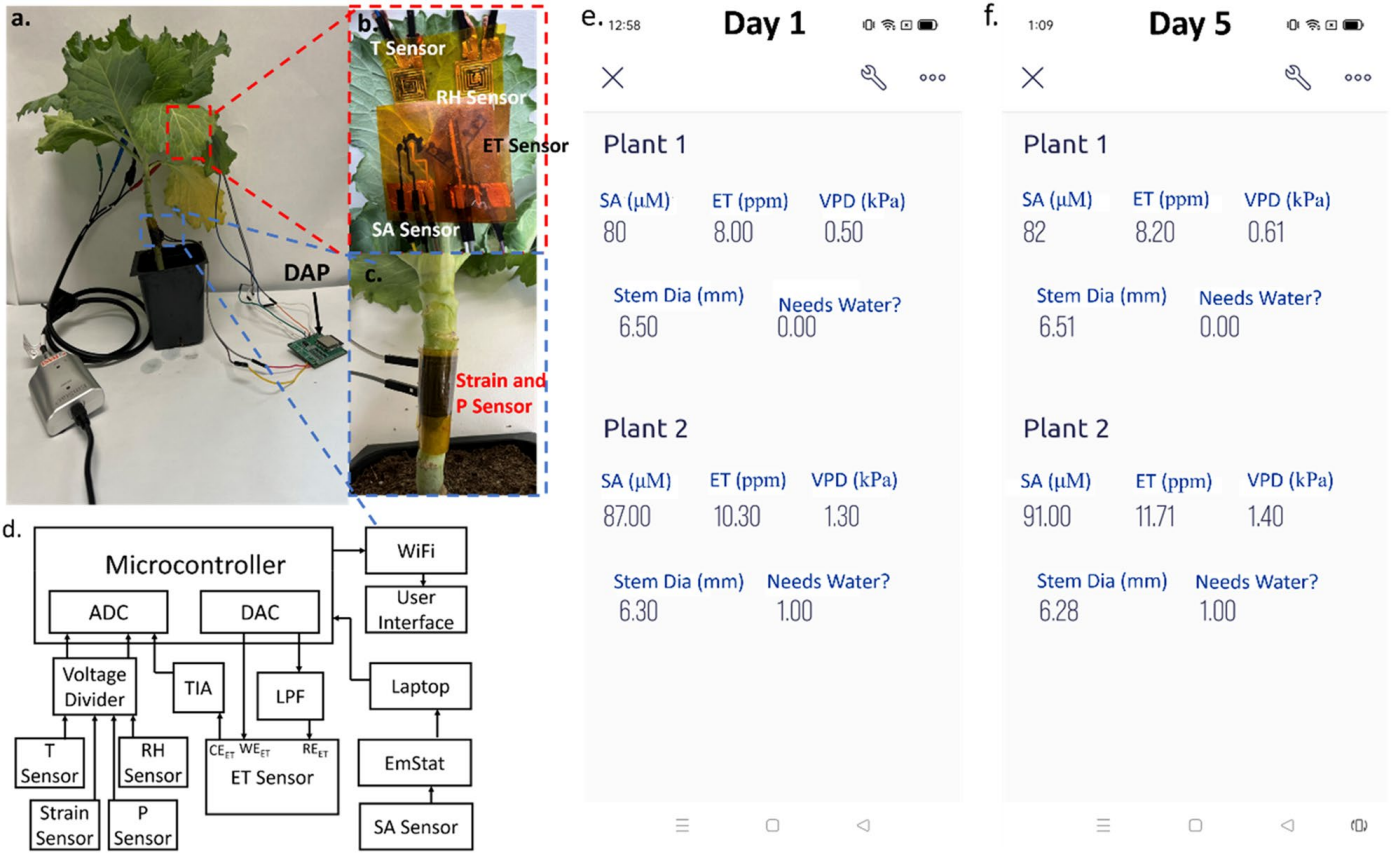
(**a**) Optical image of the plant-mounted sensor suite along with the data acquisition and processing (DAP) module. (**b**) Placement of the combined T, RH, ET, and SA sensors on the leaf. (**c**) Placement of the combined P and strain sensors on the stem. (**d**) System-level block diagram. Plant health is monitored in real-time on a smartphone app on (**e**) Day 1 and (**f**) Day 5. Here, Plant 1 and Plant 2 correspond to unstressed and water-stressed plants, respectively.

Figure 1a illustrates the optical image of the sensor suite installed on a cabbage plant and interfaced with a data acquisition and processing (DAP) module. Figures 1b,c show the placement of developed sensors on the back of the leaf and around the stem. Several reports published in the literature demonstrate that when a thin adhesive tape is used to attach the sensor at the back of the leaf, an air gap is created between the sensor and the leaf surface, thereby allowing transpiration to occur without any interference^36,37,45^. We developed a voltage divider circuit to measure the resistance variations of the temperature (T), relative humidity (RH), pressure (P), and strain sensors, and a potentiostat circuit capable of conducting cyclic voltammetry on the ethylene (ET) sensor. The main processing unit of the data acquisition and processing (DAP) module was an ESP32 microcontroller with in-built WiFi capability.

The resistance variations of the T, RH, P, and strain sensors were converted to voltage measurements via a voltage divider circuit, as shown in Fig. 1d. A constant voltage of 3.3 V was applied across the voltage divider and the auto-ranging functionality was adopted to select a known resistor from a specifically identified range. The ESP32 microcontroller had an in-built analog-to-digital converter (ADC), which read the voltage across the T, RH, P, and strain sensors and converted the analog voltages to digital values.

To obtain cyclic voltammetry measurements from the ET sensor, an 8-bit digital-to-analog converter (DAC) generated a staircase voltage waveform (the excitation signal for cyclic voltammetry) and applied that across the working (WE_ET_) and reference (RE_ET_) electrodes of the ET sensor (Fig. 1d). Two staircase waveforms were generated by two DAC modules to verify the accuracy of the excitation signal. Primarily, the timing sequence of the two staircase waveforms was measured. The timing sequence that had less than 1% deviation compared to the software timer, was selected as the excitation signal to run cyclic voltammetry. We used ESP32’s internal RC oscillator and the quartz crystal-based external clock to generate the two staircase waveforms. The utilization of two distinct oscillators for generating two waveforms introduced the delay. Since the same microcontroller (ESP32) was used for all computations and consistent programming conditions were maintained (primarily keeping the default oscillation at 125 kHz), a reproducible delay was obtained. The ESP32 microcontroller had in-built DACs to perform this operation. A low pass filter (LPF) was added between the DAC and the reference (RE_ET_) electrode of the ET sensor to remove high-frequency noise from the signal. A trans-impedance amplifier (TIA) converted the analog current measured across the working (WE_ET_) and counter (CE_ET_) electrodes of the ET sensor into a voltage signal that was read by the in-built ADC of the ESP32 microcontroller.

For the T, RH, P, strain, and ET sensors, direct memory access (DMA) operation was used to transfer the intermediate ADC reading to the specified memory space. The voltage value, V, was calculated from the ADC reading following the equation $V\,=\, \frac{ADC\, Reading*DC\, input\, voltage}{{2}^{n}-1}$ (where, DC input voltage = 3.3 V and n = 8). Data from all the sensors were processed by the microcontroller to estimate the unknown T, RH, P, strain, and ET measurements from previously stored calibration plots.

The SA sensor employed a ratiometric approach wherein the ratio of the two oxidation peak currents was used as the response signal. Due to this detection method, differential pulse voltammetry (DPV) was found more suitable to investigate the electrocatalytic activity of the SA sensor. In this regard, the commercially available EmStat Potentiostat was used to conduct DPV measurements. In the future, we will develop our own DPV circuit and integrate that into the rest of the data acquisition and processing (DAP) module.

Finally, the hormone levels (SA and ET), temperature, humidity, pressure, and stem diameter measurements were sent to the cloud wirelessly and accessed on a smartphone application via the Blynk IoT interfacing, as shown in Fig. 1e,f. For demonstration purposes, we chose two plants wherein plant 1 was unstressed and plant 2 was subjected to water deficiency from Day 1. It can be observed on the app screen that the water-stressed plant showed higher levels of SA and ET on Day 5 as compared to the measurements observed on Day 1. However, the change in VPD levels was nearly the same in both plants, indicating the potential of the hormone levels in providing an early indication of water deficiency, which is demonstrated by the ‘Needs Water’ prompt on the app screen.

## Characterization results

### Sensors calibration

**Figure 2:**
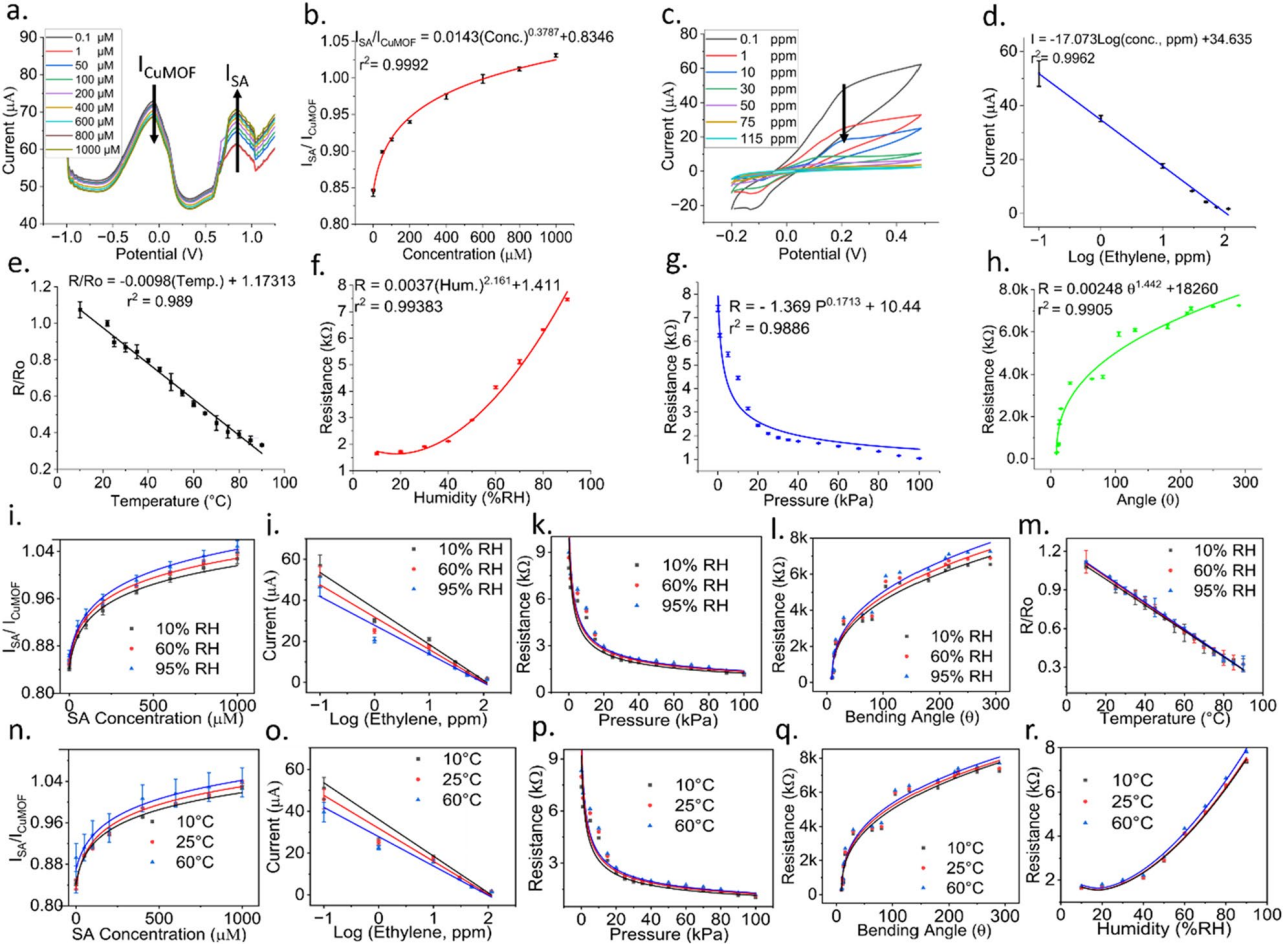
(**a**) DPV responses of the SA sensor in response to varying concentrations of Salicylic Acid. (**b**) Calibration curve of the SA sensor indicating the I_SA_/I_CuMOF_ vs. SA concentrations. (**c**) CV responses for different concentrations of ethylene. (**d**) Calibration of ethylene sensor representing the peak current vs. logarithm of the ethylene concentration. Calibration curves of (**e**) temperature sensor, (**f**) humidity sensor, (**g**) pressure sensor, and (**h**) strain sensor. Different (**i**–**m**) humidity and (**n–r**) temperature responses of the sensors. All measurements were repeated 3 times, and the error bars represent mean and standard error.

Electrochemical characterizations were performed for both salicylic acid and ethylene sensors. The salicylic acid sensor was made with a composite coating of copper metal–organic framework-carbon black-Nafion, while the ethylene sensor was fabricated with a composite copper complex (I)-single-walled carbon nanotube coating (details in Section S1 of the supporting information). First, differential pulse voltammetry (DPV) was performed in a 0.05 M tris HCl electrolyte (pH = 7.1) to calibrate the salicylic acid sensor. DPV test was performed with the commercially available potentiostat EmStat (PalmSense, Houten, Netherlands). We used voltage values in the range from − 1.0 V to 1.5 V with a 0.01 V step and scan rate of 10 mV/s. The magnitude and duration of the pulse (E_pulse_ and t_pulse_) were 0.3 V and 0.1 s, respectively. The SA sensor was calibrated with 0.1 µM, 1 µM, 50 µM, 100 µM, 200 µM, 400 µM, 600 µM, 800 µM and 1000 µM of SA. The DPV response had two redox peaks, as shown in Fig. 2a. The current peak (I_CuMOF_) located at approximately − 0.2 V was due to the reduction of Cu^2+^ in the CuMOF coating, whereas the peak (I_SA_) at 0.85 V was attributed to SA oxidation. It is noteworthy to mention that with increasing concentrations of SA, the oxidation peak current at 0.85 V increased. Simultaneously, the reduction peak current at − 0.2 V diminished owing to the increased reduction of Cu^2+^ ions. Because of the considerable separation of 1.05 V between the Cu^2+^ and the SA peaks, the ratio of two peak currents (I_SA_/ I_CuMOF_) was used as the sensor response. The DPV plots generated with EmStat were saved in an Excel file. From the potential range of -0.5 to 0 V, the maximum value is extracted, representing the peak current of I_CuMOF_. Similarly, from the potential range of 0 to 1.2 V, the maximum value is extracted, representing the peak current of I_SA_. Figure 2a and b show the DPV responses of the SA sensor and the corresponding calibration curve fitted with a power series, respectively.

Cyclic voltammetry (CV) was used to characterize the ethylene sensor. We constructed a potentiostat circuit and interfaced it with a microcontroller to carry out the CV tests, as shown in Fig. 1d. CV was performed from − 0.2 V to 0.5 V at a scan rate of 50 mV/s and potential step (i.e. E_step_) of 0.01 V. The sensor was exposed to varying concentrations of gaseous ethylene, including 0.1 ppm, 1 ppm, 10 ppm, 30 ppm, 50 ppm, 75 ppm, and 115 ppm. The CV plots shown in Fig. 2c indicate that the redox peak occurred between 0.13 V and 0.2 V. Upon exposure to ethylene, copper complex (I) bound with ethylene to form a second complex, limiting its interactions with the single-walled carbon nanotubes. As a result, the conductivity of the single-walled carbon nanotubes decreased, and hence current decreased. With an increased concentration of ethylene, there was a proportional reduction in the peak current (Fig. 2c). The microcontroller stored current values measured within the potential range from 0 to 0.4 V in an array. Using a linear search algorithm, the maximum current value of the array was identified. This maximum current value was then substituted in the stored calibration equation to convert it into the corresponding ethylene concentration. The peak oxidation current values were plotted as a function of ethylene concentrations in the logarithm scale to generate the calibration curve shown in Fig. 2d. The peak current showed a linear relation to varying ethylene concentrations. Details of the gas sensing setup is provided in Section S1.5 of the Supporting Information.

The temperature, humidity, pressure, and strain sensors were resistive by nature. This is attributed to resistance variations in response to temperature, humidity, pressure, or strain variations. The temperature sensor was fabricated with a Poly(3,4-ethylenedioxythiophene): poly(styrenesulfonate) (PEDOT:PSS) coating, and the relative humidity sensor was composed of functionalized multiwalled carbon nanotube-hydroxyethyl cellulose coating, the pressure sensor had a porous polydimethylsiloxane- deep eutectic solvent-carbon black (PDMS:DES:CB) framework, and the strain sensor was made with reduced graphene oxide (rGO) (details in Section S1 of the supporting information). The sensors were calibrated at an operating frequency of 100 Hz. The temperature sensor was calibrated with temperature values ranging from 10 °C to 90 °C. As PEDOT:PSS has a negative temperature coefficient of resistance, its resistance decreases with increasing temperature^50^. The calibration curve of the temperature sensor showed a high degree of linearity with a Pearson coefficient of 0.9899 (Fig. 2e). Next, the humidity sensor was calibrated for relative humidity values ranging from 10 to 90%. With increasing relative humidity, the resistance also increased, as demonstrated in Fig. 2f. The resistance versus relative humidity measurements was fitted with a power series having r^2^ = 0.99383. Similarly, the pressure sensor was calibrated with various pressure values ranging from 0.1 kPa to 100 kPa. As the pressure increased, the resistance of the pressure sensor decreased (Fig. 2g), confirming the negative pressure coefficient of resistance of PDMS:DES:CB^51^. The strain sensor was calibrated for various angles of curvature, as shown in Fig. 2h. As the stem grows radially, the strain sensor encounters a proportional change in its resistance. The radial growth of the stem was mimicked by cylindrical blocks of various radii (and hence various angles of curvature), as was done in our preliminary results presented at the 2021 IEEE Sensors Conference^52^. The equation that relates the angle of curvature of the strain sensor and stem radius is given by:

1 $$\theta =\frac{360 S}{2\pi r},$$

where s, r, and θ represent arc length, radius, and angle of curvature, respectively. Here, the arc length, s, is the same as the length of the sensor (2 cm). Cylindrical blocks of various radii (r = 1.8 cm, 1.43 cm, 1.25 cm, 1.09 cm, 0.88 cm, 0.72 cm, 0.63 cm, 0.54 cm, 0.53 cm, 0.45 cm, and 0.4 cm) were printed using a stereolithography 3D printer (Form 3B, Formlabs, Somerville, MA). These r values cover the stems of small plants such as bell pepper (stem diameter = 0.6 cm), cucumber (stem diameter = 1.1 cm), squash (stem diameter = 1.3 cm), tomato (stem diameter = 1.34 cm), and maize (stem diameter = 2.8 cm). The strain sensor was mounted on the cylindrical blocks and the variation in sensor resistance was measured. The r values were substituted into Eq. (1) to get the angles of curvature ranging from 8.98° to 290° (Fig. 2h).

The gauge factor (GF = $\frac{\frac{\Delta R}{R}}{\varepsilon }$), defined as the ratio of relative change in sensor resistance to the mechanical strain, was found to be 842 under a bending strain of 1.4%. Equation (2) shows the equation for the mechanical bending strain, ε

2 $$\upvarepsilon = \frac{t}{2{r}_{b}},$$

where t is the combined thickness of the polyimide sheet and the overlaid sensing layers (= 127 μm) and r_b_ is the bending radius of the sensor under the bending state. The bending radius, r_b_, was calculated following the method outlined in Ref.^53^ as was also done in our prior work^52^. Figure S9a in the supporting information shows the gauge factor versus the bending strain plot. A motorized translation stage (MTS50-Z8, Thorlabs Inc., Newton, NJ, USA) was used to measure the bending radius, as illustrated in Fig. S9b in the supporting information. The resolution, defined as the smallest detectable change in the angle of curvature in response to the radial growth in the stem, was calculated to be approximately 0.06°.

The salicylic acid and ethylene sensors could operate effectively for a total of 60 uses when utilized once a day. These sensors required recalibration every 7 days, as the measured response reduced by approximately 0.85%. In contrast, the physical sensors (temperature, humidity, strain, and pressure) were used for 60 days with 4 uses per day (a total of 240 uses). The physical sensors did not require recalibration during their lifetime. The limited lifetime of salicylic acid and ethylene sensors was primarily due to the chemical reactions occurring on the working electrodes, thus replenishing or saturating the functionalized coating materials.

To maintain cost-effectiveness, the salicylic acid and ethylene sensors were removed from the plant after 7 days, recalibrated, and then repositioned (although this may pose some inconvenience for wearable operation). However, in the future, if these sensors are extensively deployed in the field, the microprocessor can be programmed to perform onsite dynamic calibration without removing the sensors, which will maintain both wearability and cost-effectiveness.

### Sensitivity and LOD analysis

The calibration curves for salicylic acid, humidity, pressure, and strain sensors were fitted with power series (${y=ax}^{b}+c$). The sensitivity,${S}_{y}{|}_{x}$, was calculated by the method described in Ref.^54^.

3 $${S}_{y}{|}_{x} := \frac{dy}{dx} =ab{x}^{b-1},$$

where x and y represent the target parameter (i.e. SA concentration/RH/pressure/angle of curvature depending on the sensor type) and sensor response, respectively, while a and b denote parameters of the fitted curve. Sensitivity values were calculated at both the lowest and highest x values. In contrast, ethylene and temperature sensors exhibited a linear response (Fig. 2d and e) and hence the slope (m) of the linear fit ($$y=mx+c)$$ was used as a measure of sensitivity.

The limit of detection (LOD) for the physical sensors, i.e. temperature, humidity, pressure, and strain sensors, was calculated using the following formula:

4 $$LOD= \frac{3* std. dev.}{Sensitivity}.$$

The LOD for chemical sensors, i.e. SA and ethylene sensors, was calculated using the following sets of equations^55^:

5 $$LOB\,=\,mean\, of\, signal\, \left(blank \,sample\right)+1.645\, \left( std. \,dev.\, of\, blank\, sample\right),$$

6 $$yLOD=LOB+1.645 \left( std. \,dev.\, of\, target\, at\, low \,concentration\right),$$

7 $$LOD=\frac{yLOD-intercept}{slope}.$$

Tables 1 and 2 summarize the sensitivity, LOD, and resolution for all the sensors. The steps for calculating the resolution are shown in Section S4 in the Supporting Information.

**Table 1:** Performance metrics of non-linear sensors.

Sensor	Equation	Low concentration sensitivity	High concentration sensitivity	LOD	Resolution
Salicylic acid, SA	I_SA_/I_CuMOF_ = $0.0143{(SA)}^{0.3787}+0.8346$	0.002264 μM^-1^ (at 0.1 μM)	7.409 X 10^−5^ μM^−1^ (at 1000 μM)	0.644 μM	0.30 μM
Humidity, RH	R = 0.0037 $({RH)}^{2.161}+1.411$	0.011589 kΩ/(%RH) (at 10%RH)	0.1485 kΩ/(%RH) (at 90%RH)	11.321%RH	0.24%RH
Pressure, P	R=$-$ 1.369 ${P}^{0.1713}+10.44$	5.04 kΩ/(kPa) (at 0.1 kPa)	0.0164 kΩ/(kPa) (at 100 kPa)	0.3733 kPa	0.11 kPa
Strain,$\theta$	R = 0.00248 ${\theta }^{1.442}+18260$	1.5935 X 10^–6^ kΩ/° (Gauge factor = 100)	1.006 X 10^−8^ kΩ/°(Gauge factor = 900)	9.3211° (0.1%)	0.06°

**Table 2:** Performance metrics of linear sensors.

Sensor	Equation	Sensitivity	LOD	Resolution
Ethylene, ET	I = $-17.073\mathrm{ log}\left(ET\right)+34.635$	17.073 μA/log(ppm)	0.6089 ppm	0.424 ppm
Temperature, T	$\frac{R}{Ro}=-0.0098 \left(T\right)+1.17313$	0.0098/°C	10.5478 °C	1.03 °C

### Temperature and humidity corrections

In an agricultural field setting, temperature and humidity levels change frequently. Hence, we characterized the performance of our sensors in response to varying temperature and humidity levels. All but the temperature sensor were tested and corrected for temperature variations because the temperature sensor was designed to respond to temperature variations. Likewise, all but the humidity sensor were tested and corrected for humidity variations. Figure 2i–m show the calibration plots of the sensors under varying relative humidity levels. Likewise, Fig. 2n–r show the responses of all but the temperature sensor under varying temperature conditions. The coefficient of variance between the calibration plots of each sensor was found to be less than 9%.

The correction factors for the intercept, slope, and exponent (for nonlinear curve fit) were calculated at different temperature and humidity levels using the following equations^55^. Room temperature (25 °C) and humidity (60%RH) were considered as references.

8 $${\mathrm{f}}_{\mathrm{intercept}(\mathrm{temp})} = \frac{intercept(temp)}{intercept(25\,\,^\circ{\text{C}})} \quad {\mathrm{f}}_{\mathrm{intercept}\left(\mathrm{\%RH}\right)}= \frac{intercept(\mathrm{\%}RH)}{intercept(\mathrm{\%}60)},$$

9 $${\mathrm{f}}_{\mathrm{slope}(\mathrm{temp})} = \frac{slope(temp)}{slope(25\,\,^\circ {\text{C}})} \quad {\mathrm{f}}_{\mathrm{slope}(\mathrm{\%RH})}=\frac{slope(\%RH)}{slope(\mathrm{\%}60)},$$

10 $${\mathrm{f}}_{\mathrm{exponent}\left(\mathrm{temp}\right)}= \frac{exponent(temp)}{exponent(25\,\,^\circ {\text{C}})} \quad {\mathrm{f}}_{\mathrm{exponent}\left(\mathrm{\%RH}\right)} = \frac{exponent(\mathrm{\%}RH)}{exponent(\mathrm{\%}60)},$$

### Repeatability, reproducibility, bending, and hysteresis studies

**Figure 3:**
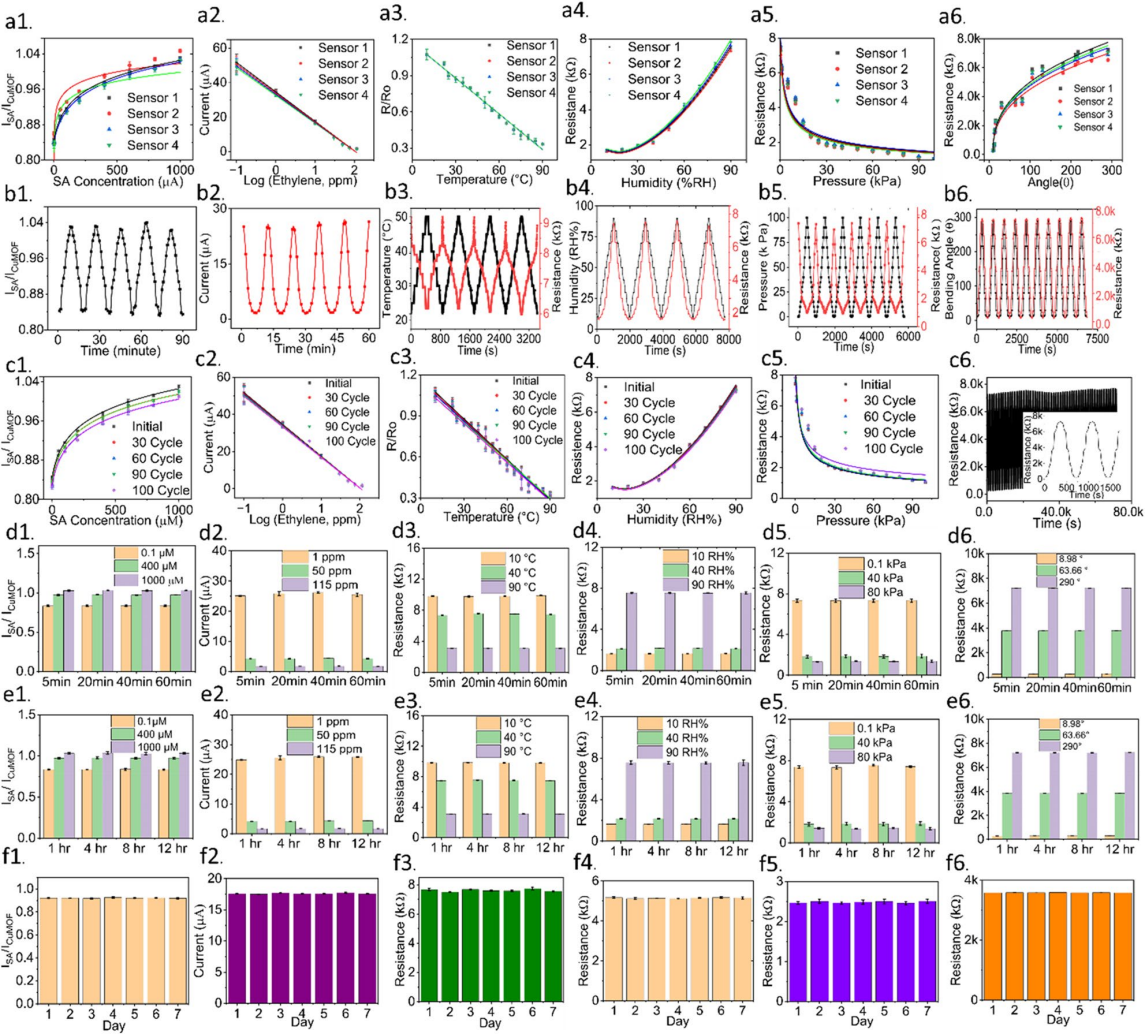
Reproducibility test for (**a1**) salicylic acid, (**a2**) ethylene, (**a3**) temperature, (**a4**) humidity, (**a5**) pressure, and (**a6**) strain sensors. Repeatability test for (**b1**) salicylic acid, (**b2**) ethylene, (**b3**) temperature (left axis: temperature, right axis: corresponding resistance), (**b4**) humidity(left axis: humidity, right axis: corresponding resistance), (**b5**) pressure(left axis: pressure, right axis: corresponding resistance), and (**b6**) strain sensors(left axis: bending angle, right axis: corresponding resistance). In **b3–b6**, the red-colored plots represent resistance. Bending test for (**c1**) salicylic acid, (**c2**) ethylene, (**c3**) temperature, (**c4**) humidity, and (**c5**) pressure sensors. All the sensors were repeatedly bent at 45° angles and the calibration curves are plotted after different cycles of bending. (**c6**) Bending of the strain sensor at 45° angles for 1000 cycles. Investigating drift over 1 h for (**d1**) salicylic acid sensor at 0.1, 400, and 1000 μM concentrations, (**d2**) ethylene sensor at 1, 50, and 115 ppm, (**d3**) temperature sensor at 10, 40, and 90 °C, (**d4**) humidity sensor at 10, 40, and 90 RH%, (**d5**) pressure sensor at 0.1, 40, and 80 kPa, and (**d6**) strain sensor at 8.98, 63.66, and 290° bending angles. Investigating drift over 12 h for (**e1**) SA sensor at 0.1, 400, and 1000 μM concentrations, (**e2**) ethylene sensor at 1, 50, and 115 ppm, (**e3**) temperature sensor at 10, 40, and 90 °C, (**e4**) humidity sensor at 10, 40, and 90 RH%, (**e5**) pressure sensor at 0.1, 40, and 80 kPa, and (**e6**) strain sensor at 8.98, 63.66, and 290° bending angles. The long-term stability of (**f1**) salicylic acid sensor (tested with 100 μM), (**f2**) ethylene sensor (tested with 10 ppm), (**f3**) temperature sensor (tested with 35 °C), (f4) humidity sensor (tested with 75% RH), (**f5**) pressure sensor (tested with 20 kPa), and (**f6**) strain sensor (testing with 30° bending angle). All measurements were repeated 3 times, with the error bars representing mean and standard error.

All six sensors were tested for repeatability, reproducibility, bending, and hysteresis, to confirm their feasibility of field deployment. Reproducibility was tested by repeating the calibration with four identical sensors from each category (Fig. 3a1–a6). The coefficient of variance for four repeated measurements was found to be less than 3%. The sensors also demonstrated repeatable characteristics under cyclic variations in SA (Fig. 3b1), ethylene (Fig. 3b2), temperature (Fig. 3b3), humidity (Fig. 3b4), pressure (Fig. 3b5), and strain (Fig. 3b6). For instance, the SA sensor was exposed to increasing, followed by decreasing concentrations of SA, and the cycle was repeated five times. The same SA concentration values (i.e. 0.1 µM, 1 µM, 50 µM, 100 µM, 200 µM, 400 µM, 600 µM, 800 µM, and 1000 µM) as shown in Fig. 2b were used for the repeatability test. A similar procedure was adopted for investigating the repeatable characteristics of other sensors. All the sensors demonstrated a coefficient of variance of less than 5%, which is reasonable for in-field operation. Figure S10 in the supporting information shows the dynamic response of all sensors over a half cycle. The responses were measured with the custom-made data acquisition and processing module shown in Fig. 1d. All the sensors demonstrated a rapid response time of less than a minute.

The flexible substrate carrying the sensors was subjected to 30, 60, 90, and 100 cycles of bending at an angle of 45°, and the calibration curve of each sensor was repeated (Fig. 3c1–c5). It was observed that even after 100 cycles of bending, the coefficient of variance between the calibration curves remained less than 3%. The hysteresis between the 0th and 100th cycles of bending was calculated to be less than 3% for all but the SA sensor (Fig. S11 in Supporting Information). A higher hysteresis for the SA sensor could be attributed to the crack formation in the carbon black coating after 100 cycles of bending^56^, which resulted in a degradation in the sensor performance by 1.23%. Generally, 45^0^ bending of the leaf surface may not occur in a live plant. Yet, if needed a separate correction factor can be introduced to account for such performance degradation under large bending angles, as is explained below. The response of the strain sensor was recorded for 1000 cycles of repeated bending, as shown in Fig. 3c6. Nevertheless, the hysteresis was found to be less than 3% even after subjecting the strain sensor to 1000 cycles of bending (Fig. S11 in Supporting Information).

The corrected intercept, slope, and exponent of the sensors were found using the values computed in Eqs. (8), (9) and (10)^55^:

11 $$intercept\left(corr.\right)={\mathrm{f}}_{\mathrm{intercept}}\left(\mathrm{temp}\right) {\mathrm{f}}_{\mathrm{intercept}}\left(\mathrm{\%RH}\right) \,\, intercept\left(init.\right),$$

12 $$slope\left(corr.\right)= {\mathrm{f}}_{\mathrm{slope}}\left(\mathrm{temp}\right){\mathrm{f}}_{\mathrm{slope}}\left(\mathrm{\%RH}\right) \,\, slope\left(init.\right),$$

13 $$exponent\left(corr.\right)= {\mathrm{f}}_{\mathrm{exponent}}\left(\mathrm{temp}\right){\mathrm{f}}_{\mathrm{exponent}}\left(\mathrm{\%RH}\right)\,\, exponent\left(init.\right).$$

In the future, a separate strain sensor can be integrated with the leaf patch and the hormone measurements (i.e. SA and ethylene levels) can be corrected by recalculating the slope, intercept, and exponent of the initial calibration graphs.

**Figure 4:**
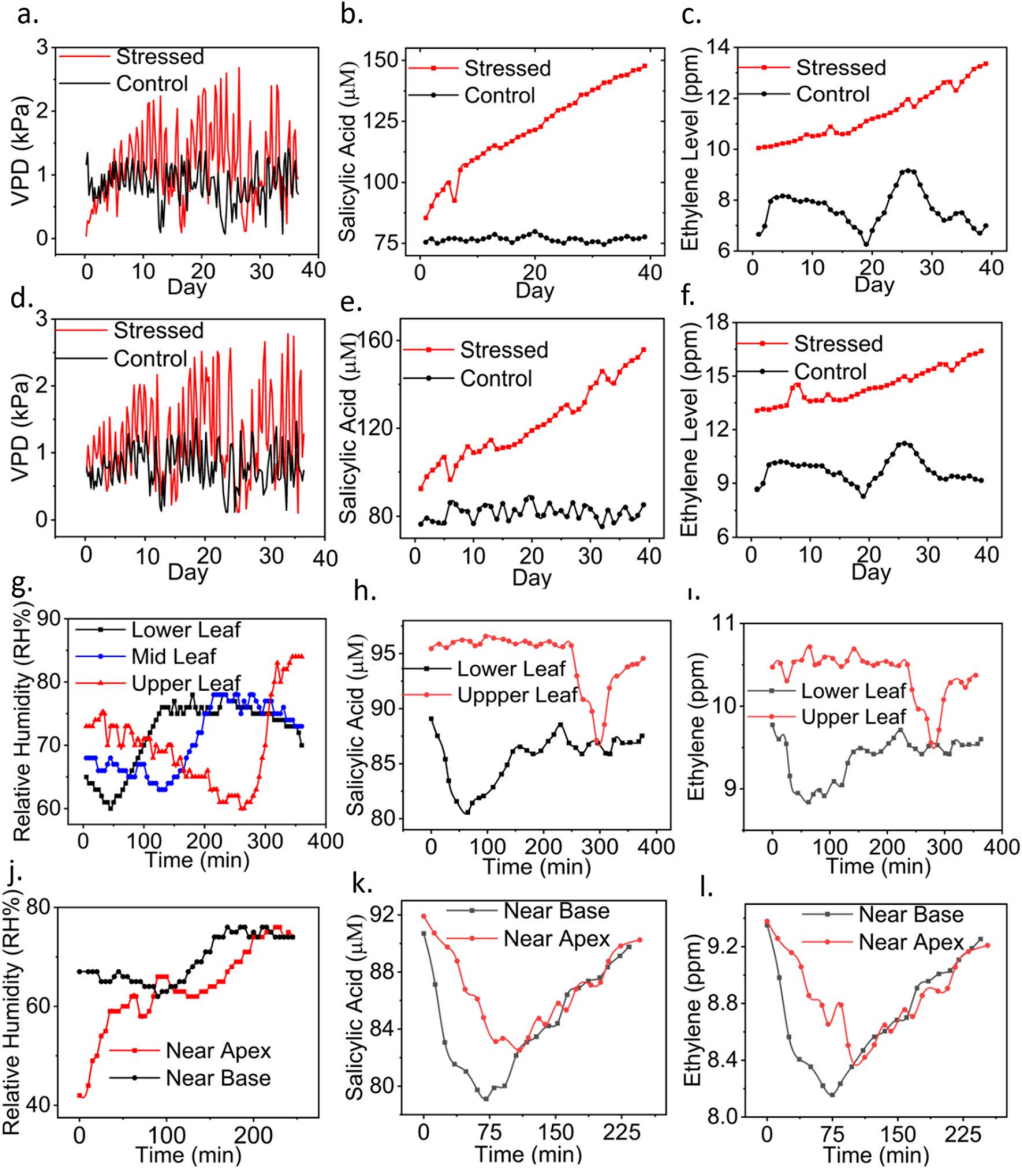
Continuous measurements of (**a**) VPD, (**b**) SA, and (**c**) ethylene levels in control and water-stressed bell pepper plants kept in sunlight. Continuous measurements of (**d**) VPD, (**e**) SA, and (**f**) ethylene levels in control and water-stressed bell pepper plants kept in shade. Monitoring water, SA, and ethylene transport across a bell pepper plant. Sensors were installed at three leaves, located at lower = 40 cm, middle = 75 cm, and upper = 105 cm leaves. (**g**) Dynamic RH changes at the lower, middle, and upper leaves. (**h**) Dynamic SA changes at lower and upper leaves. (**i**) Dynamic ethylene changes at lower and upper leaves. Dynamic (**j**) RH, (**k**) SA, and (**l**) ethylene changes in two areas (i.e. near base and apex) of the same leaf that was located 75 cm above the soil surface. The two sensors were placed 5 cm apart.

**Figure 5:**
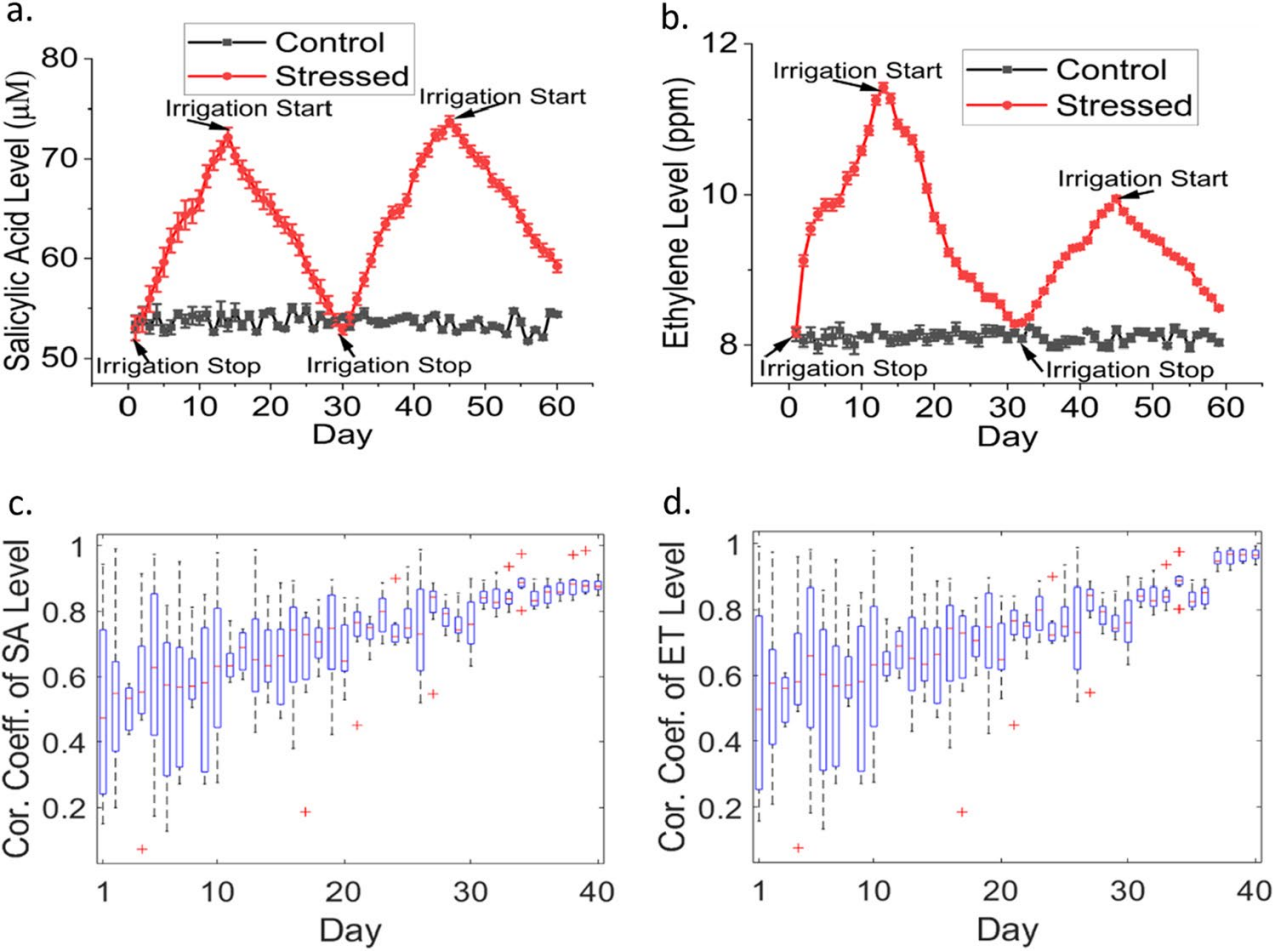
Continuous measurements of (**a**) SA and (**b**) ethylene levels in the leaf under periodic water stress and irrigation. Measurements were repeated with 10 stressed plants and 10 control plants, with error bars representing the plant-to-plant variations. Autocorrelation studies of (**c**) SA (**d**) ethylene levels in stressed plants.

The repeatability and reproducibility tests for salicylic acid, ethylene, pressure, and strain sensors were done at 25 °C temperature and 60% relative humidity (RH). The temperature sensor was tested for repeatability and reproducibility at a constant RH of 60%, while the temperature was varied. Likewise, the humidity sensor was tested at a constant room temperature of 25 °C, while the humidity was varied. For real-time data collected from the plants, Figs. 4 and 5 show salicylic acid (SA) and ethylene (ET) levels after correcting for any changes caused by variations in the temperature (T) and relative humidity (RH). Similarly, the stem diameter measurements reported in Table S3 of the Supporting Information were T and RH corrected. Hence, to further demonstrate the dependence of the sensor measurements on T and RH in a dynamic environment, Fig. S12 in the Supporting Information shows corrected and uncorrected (i.e., raw) measurements of SA, ET, stem diameter, and pressure over 5 days in water-stressed bell pepper plants.

### Drift analysis

The sensors were characterized for drift by first measuring the sensor response every 20 min over an hour (Fig. 3d1–d6) and then every 4 h over 12 h (Fig. 3e1–e6). The mean coefficient of variance was < 2%, indicating the minimal drift displayed by the sensors.

### Stability analysis

The long-term stability of the six sensors was evaluated over a week. The sensor responses are demonstrated in Fig. 3f1–f6. The coefficient of variance in the sensor response was measured to be < 2% over 7 days, indicating an acceptable stable response for in-plant measurements.

### Selectivity analysis

The details of selectivity analysis are described in Section S5 and the results are plotted in Fig. S13 of the supporting information. The salicylic acid and ethylene sensors showed a high degree of selectivity to other interfering species.

## Real-time plant measurements

### Water stress experiments in bell pepper plants

A few pilot experiments were conducted under field conditions in an outdoor garden located at The University of Texas at Tyler (32.3163°N and − 95.2510°W). The sensors were first tested with live bell pepper plants. The plants were purchased from a local nursery. The sensor suite comprising the temperature, humidity, SA, and ethylene sensors was installed at the back of the leaves using a thin adhesive tape. The combined flexible patch carrying the strain and pressure sensors was wrapped around the stem. A micron scale (10 µm) hole was punched into the leaf so that sap from the vascular bundle could reach the sensor surface for SA detection^45^. Measurements were recorded from 4 bell pepper plants, where plant #1 and plant #2 were water-stressed and control (i.e. unstressed) plants kept in sunlight, respectively, and plant #3 and plant #4 were water-stressed and control plants placed in the shade, respectively. The stressed plants were deprived of water during the stress period, while the control plants were irrigated with 50 mL of water every day at 9:30 am. No fertilizers or insecticides were applied. Measurements were collected from all 6 sensors for 40 days (from September 18, 2021, to October 27, 2021). Temperature and humidity measurements were recorded four times a day (9:30 am, 1:00 pm, 5:00 pm, and 8:00 pm), while SA, ethylene, and pressure-corrected stem diameter were measured once a day (at 1:00 pm). The results are demonstrated in Fig. 4a–f and Table S3 in the supporting information. The SA and ethylene levels depicted in Fig. 4b, c, e and f were calculated after correcting for variations in leaf microclimate (i.e. temperature and humidity), according to Eqs. (8, 9, 10, 11, 12 and 13). Figure S14 in the supporting information shows the optical images of water-stressed and unstressed bell pepper plants taken after 40 days of measurements.

Based on the evidence in the literature, variations in plant physiology (i.e., changes in hormone levels, temperature, and humidity) are monitored at different intervals based on the conditions the plant is exposed to. For instance, researchers monitored the levels of salicylic acid once a day to analyze parameters such as photosynthesis, stomatal behavior, plant growth, and cell membrane integrity^57^. In another work, the response of salicylic acid is monitored on an hourly basis following the introduction of a plant pathogen^58^. The dynamic response of various phytohormones including abscisic acid, jasmonic acid, indole-3-acetic acid, Brassinosteroids, and gibberellic acid, has been examined at intervals ranging from 15 min to 24 h in Arabidopsis thaliana plants under different stress conditions such as wounding, UV exposure, cold stress, heat stress, drought stress, and salinity stress. Consequently, this study concluded that significant changes in phytohormone responses occur within 15 min followed by a wounding effect^59^. Researchers also evaluated the metabolic changes in soybean plants by measuring the temperature and humidity on an hourly basis^60^. Our sensors, with a response time of less than a minute, are capable of measuring these changes directly on-site. If a more frequent analysis (e.g., down to every minute) is necessary, our sensors can accommodate that need also. Unlike existing monitoring techniques that involve the destructive collection of plant samples, which adds additional stress to the plant when collected more frequently, our in situ sensors eliminate the need for sample collection and enable more frequent sensing in a minimally invasive manner.

Vapor pressure deficit (VPD) was calculated using the following Eqs. ^61^:

14 $$VPD=VPsat-VPair,$$

15 $$VPsat=0.6107 \times{10}^{\frac{7.5T1}{ 237.3+T1}},$$

16 $$VPair=0.6107 \times{10 }^{\frac{7.5Ta}{237.3+Ta}} \times \frac{RH}{100},$$

where VP_sat_ = saturated vapor pressure inside the leaf (in kPa) and VP_air_ = vapor pressure of the air. T_l_ and T_a_ represent temperatures on the leaf surface and in the air (in °C), respectively and RH is the relative humidity at the leaf surface.

The variations in leaf temperature and humidity agree with evidence found in the literature. Leaf temperature (in both stressed and control plants) was always found lower than the air temperature, which was due to the cooling produced by transpiration. In addition, the relative humidity of air was lower than the relative humidity levels measured beneath the leaf^36^, which was a driving factor for transpiration. The real-time VPD, leaf RH, and leaf temperature measurements recorded over 10 days are plotted in Fig. S15 of the supporting information. VPD is an effective measure of the transportation of water from root to shoot. Increased VPD leads to rapid transpiration in plants, which results in over-drying and stressing of the plants. In contrast, a lower VPD value indicates vapor saturation on the leaf surface, which can lead to fungal infection in leaves^24^. Hence, it is crucial to maintain an optimum VPD level in plants. VPD values measured with our temperature and humidity sensors were found to be higher in the water-stressed plants as compared to the control plants, indicating the closure of stomata and hence the reluctance of the plant to transpire. It is noteworthy that stomatal closure is a common adaptation response of plants to the onset of drought^62^. This can also be explained mathematically with Eqs. (14), (15) and (16). VPD is determined by the combination of VPsat and VPair. In response to water stress, VPsat increases as the leaf temperature rises. Although VPair is influenced by air temperature (T_a_) and leaf relative humidity (RH), the adjustment factor (RH/100) in Eq. (16) is insignificant (less than 1) and cannot compensate for the increasing VPsat value. Hence, with the leaf temperature (T_1_ in Eq. (15)) rising and the RH/100 factor in Eq. (16) causing VPair to decrease rapidly in water-stressed plants, the overall VPD increases (please note Eq. (14)).

The microscopic images of leaves further confirmed stomata closure in water-stressed plants and opening in control plants (Figs. S16 and S17 of supporting information). VPD levels in the water-stressed plant kept in the sunlight (Fig. 4a) increased faster than the levels measured in the water-stressed plant kept in the shade (Fig. 4d). This finding suggests that the plants continuously exposed to the sun encountered additional heat stress, resulting in an elevated level of VPD. We measured VPD three times during the day and once at night. As nightfall occurs, there is a decrease in temperature in the open air, leading to a corresponding decrease in VPD. Similar oscillations in VPD measurements, both in the short-term and long-term, have been observed in previous literature^36,63^. According to the literature^64^, an increasing trend in VPD is considered a crucial indicator of water stress conditions in plants, despite the presence of oscillations in the VPD data. Analyzing the VPD data in the context of the upward trend in the water-stressed plants and the relatively stable trend in the controlled plants helps make sense of the VPD data.

Moreover, a progressive increase in SA and ethylene levels was observed in water-stressed plants starting from Day 1 (Fig. 4b, c, e, f). The SA levels in the control plants fluctuated around a mean of 75.35 μM with a standard deviation of 2.523 μM in Fig. 4b and a mean of 80.31 μM with a standard deviation of 3.226 μM in Fig. 4e. Likewise, the ethylene levels in the control plants fluctuated around a mean of 8.81 ppm with a minimal standard deviation of 0.311 ppm in Fig. 4c and a mean of 10.21 ppm with a standard deviation of 0.336 ppm in Fig. 4f. Therefore, the sensor demonstrated its ability to distinguish hormone emissions between stressed and unstressed plants. These findings would enable early diagnosis of crop stress from SA and ethylene levels and facilitate immediate intervention measures to reduce stress-induced productivity losses. The radial growth (derived from stem diameter) of both the water-stressed plants went down owing to water deficiency and hence the crop growth declined (see Table S3, supporting information).

In addition, the time-series SA levels measured with our sensor in the leaves of live bell pepper plants over 40 days were validated against the values from high-performance liquid chromatography, as shown in Fig. S18a–d in the supporting information. A few microliters of sap samples were collected from the plants every day over the 40 days. The samples were analyzed with high-performance liquid chromatography equipment to measure the SA concentrations. Our sensor could accurately estimate the SA concentrations in both control and water-stressed plants over 40 days. A high Pearson correlation coefficient greater than 0.92 was observed, suggesting the excellent reliability of the sensor. Due to the absence of a Flame Ionization Detector (FID) (which is required to analyze ethylene gas because ethylene is an organic compound and FID senses carbon ions with ultra-high precision^65^) extension to the gas chromatography–mass spectrometry equipment at our facility and other nearby institutional facilities, we adopted an indirect approach to analyze the ethylene gas directly emitted from plant leaves. First, we calibrated the ethylene sensor for a known set of ethylene concentrations mixed with other interfering gases (i.e., N_2,_ CH_4,_ N_2_O, and NH_3_). Next, the sensor responses were recorded for unknown ethylene concentrations emitted from the plant. Afterward, the sensor was exposed to a range of known ethylene concentrations to find out the concentrations at which the sensor response approximately matched with the responses recorded for unknown ethylene emitted from plants. The results were plotted in Fig. S18g,h, which show that our ethylene sensor has a very high accuracy, with a Pearson correlation coefficient of 0.99. Moreover, the VPD values measured with our flexible temperature and relative humidity sensors were compared against the VPD values measured with commercially available rigid temperature (LM35, Texas Instruments, TX) and humidity (DHT11, Adafruit, NY) sensors. As outlined in Tables S4 and S5 of the supporting information, our sensors show excellent accuracy with minimal deviation from the commercial sensors. It was observed that Compared to the traditional mass spectroscopy-based technique that requires expensive instrumentation, a disruptive and complex sampling process, and skilled operators, the proposed crop-wearable sensor suite allowed real-time and in situ monitoring along with an early diagnosis of stress conditions in live plants. Moreover, in contrast to commercially available rigid integrated circuits, our sensors are flexible and easily compliant to delicate parts of the plant including leaves and stems.

### Kinetics of SA and ethylene transport across bell pepper plants

The sensor suite was used to monitor water transport across a bell pepper plant (Fig. 4g–l). A correlation was observed between the hormone levels (i.e., SA and ethylene) and water transport (Fig. 4g–i). Three sensors were installed at the lower (40 cm), middle (75 cm), and upper (105 cm) leaves, with the leaf height measured from the soil surface. The plant was irrigated before the test. The transition in RH levels at the leaves located at three different heights indicate water transport from the root to the shoot. The kinetics of SA and ethylene levels were found to highly correlate with the kinetics of water transport (represented by leaf RH and VPD values). An upward transition in leaf RH was followed by a downward transition in SA and ethylene levels almost instantaneously. A decrease in the SA and ethylene levels was most likely due to water reaching the leaf. It took almost 221 min for the water to reach from lower to upper leaves. Figure S19 in the supporting information further depicts a comparative analysis of the kinetics of SA and VPD levels at the lower and upper leaves. The results again confirm the correlation between SA and VPD kinetics. Over 400 min, except for the transitions, the upper leaf emitted a greater amount of SA and ethylene (96.36 ± 4.35 μM for SA and 10.8 ± 0.22 ppm for ethylene) than the lower leaf (87.11 ± 3.5 μM for SA and 9.68 ± 0.34 ppm for ethylene), as illustrated in Fig. 4g–i. These results would advance the understanding of the relationship between sensor-measured phytohormone levels and soil water availability. Moreover, the spatiotemporal distribution of water and hormone levels across the whole plant can be investigated by mounting sensors at multiple locations of the same plant. The information gained from these experiments could be valuable to geneticists and breeders in breeding crop cultivars with specific traits such as improved productivity and high water-use efficiency under drought conditions.

Furthermore, the difference in SA and ethylene levels between the base and apex of the same leaf (located 75 cm above the soil surface) was also measured, as shown in Fig. 4j–l. For this experiment, two sensors were installed 5 cm apart on the same leaf. The differences in the RH, SA, and ethylene dynamics were observed at the leaf scale.

Figure 4j–l show an increase in salicylic acid and ethylene as relative humidity kept increasing. A direct correlation between the concentrations of endogenous salicylic acid and ethylene and relative humidity on the leaf surface has not been reported elsewhere. Based on the mechanism of transpiration and biosynthesis pathways of secondary metabolites (e.g. salicylic acid and ethylene) reported in the literature, a possible explanation could be the following. After applying water to the plant, water is transported from the roots to the leaves. When stomata are open, water vapor is lost to the external environment, resulting in an increased rate of transpiration and thus increasing the relative humidity beneath the leaf surface. Nevertheless, the relative humidity eventually reaches a saturation point over time, indicating that the plant does not have adequate available water for transpiration. As a result, the water content released through the stomata ceases to exhibit any further upward trend in relative humidity. With a continuous supply of water, plants will continue to transpire and relative humidity beneath the leaves will continue to change. In this experiment, the plant was subjected to low water stress (deficit irrigation), in order to induce water deficiency after a certain period of time. Salicylic acid and ethylene are secondary metabolites that are produced by the plant cell through metabolic pathways derived from the primary metabolic pathways^66^. The biosynthesis pathways of salicylic acid and ethylene are triggered in response to abiotic stressors such as water deficiency^67,68^. During our experiment, we observed an initial decrease in the levels of salicylic acid and ethylene as the plant began to uptake water. This downward trend is illustrated in Fig. 4k–l. After approximately 75 min, salicylic acid and ethylene levels started to increase which could be attributed to a reduction in the rate of transpiration as the relative humidity approaches saturation.

### Cyclic water stress experiments in cabbage plants

Furthermore, the sensors were demonstrated to measure SA and ethylene levels in cabbage plants under periodic water stress conditions over 2 months (from September 21, 2021, to November 19, 2021). Ten cabbage plants were subjected to water stress and ten plants were unstressed (control). The stressed plants were not irrigated during the stress period, while the control plants were irrigated with 20 mL of water every day. Two cycles of water stress were applied wherein each cycle lasted 30 days. Water stress was applied in the first 15 days, followed by irrigation in the next 15 days. This 30-day cycle was repeated. SA and ethylene levels were measured once a day (at 1:00 pm). A noticeable time-series correlation was observed between the SA/ethylene levels and the water stress periods (Fig. 5a,b). The hormone levels started to elevate in response to a water stress condition, while immediately after irrigation the hormone levels declined. The time-series SA levels measured with our sensor in the leaves of live cabbage plants over 60 days were validated against the values from high-performance liquid chromatography, as shown in Fig. S18e and f in the supporting information. It should be noted that although all the plants were grown under the same environmental conditions, plant-to-plant variations in growth, development, and metabolism were observed. Therefore, a statistical analysis was performed based on the hormone levels obtained from multiple plants. The SA and ethylene levels in the 10 water-stressed plants were analyzed via autocorrelation analysis, as shown in Fig. 5c,d. Although the autocorrelation coefficient demonstrated a large standard deviation during the first 10 days of the experiment, the deviation started to diminish eventually and reached ± 0.910589 for SA and ± 0.98633 for ethylene. This could perhaps be explained by the plant-to-plant variability in hormonal responses at the beginning. However, as days passed, the plants got acclimated to the water stress condition and exhibited a similar phytohormone response with a smaller standard deviation. Such findings would inform the optimum number of sensors needed to be deployed in a large agricultural field. For instance, we hypothesize that one sensor can be deployed per acre of the field wherein crops exhibit similar phytohormone responses driven by micro-environmental factors or soil structure/treatment. More research is needed to elucidate this hypothesis and we believe our sensors will play a pivotal role in advancing the scientific understanding of environment-plant interactions in the field scale.

Throughout the 60-day testing period on plants (as depicted in Fig. 5), there were no notable detrimental effects on plant growth caused by the attachment of the sensor suite. This was evidenced by the unchanged phytohormone response in control plants (measured with our sensor as well as verified with high-performance liquid chromatography), indicating that the presence of the sensors did not induce additional stress to the plants. The absence of any hindrance to plant growth can be attributed to the sensor's placement at the back of the leaf, with the help of a 100 µm-thick adhesive tape, which enabled the formation of a small gap between the leaf and sensor surface. This allowed unimpeded light interactions on the upper leaf surface and gas exchange through the stomata located on the underside of the leaf, which are crucial for plant physiological processes (e.g., photosynthesis). Furthermore, the lightweight sensors did not cause any leaf bending or deformation during the testing period. Notably, previous studies employing similar sensor integration techniques on plant leaves have also reported no noticeable stress on plants or growth inhibition^69–71^.

### Water stress experiments at different growth stages of tomato plants

SA, ethylene, and VPD levels were also measured at different growth stages of a plant. Tomato seedlings were grown to conduct this investigation owing to their conducive growth during the study period (from March 15, 2022, to April 3, 2022). SA, ethylene, and VPD levels were measured in plants aged 5, 10, 15, and 20 days, counted from germination. Figure S20 in the supporting information depicts the optical images of plants on different days of growing. The sensor patch was reconfigured to fit into the smallest leaf. The same sensor patch was used to measure SA, ethylene, and VPD levels at 5, 10, 15, and 20 days old plants. Figure S21 in supporting information depicts the sensor patch installed on a 15-day-old tomato plant. The calibration graphs of the modified sensors are shown in Fig. S22, supporting information, and real-time SA, ethylene, and VPD measurements are shown in Fig. S23, supporting information.

### Feature analysis

**Figure 6:**
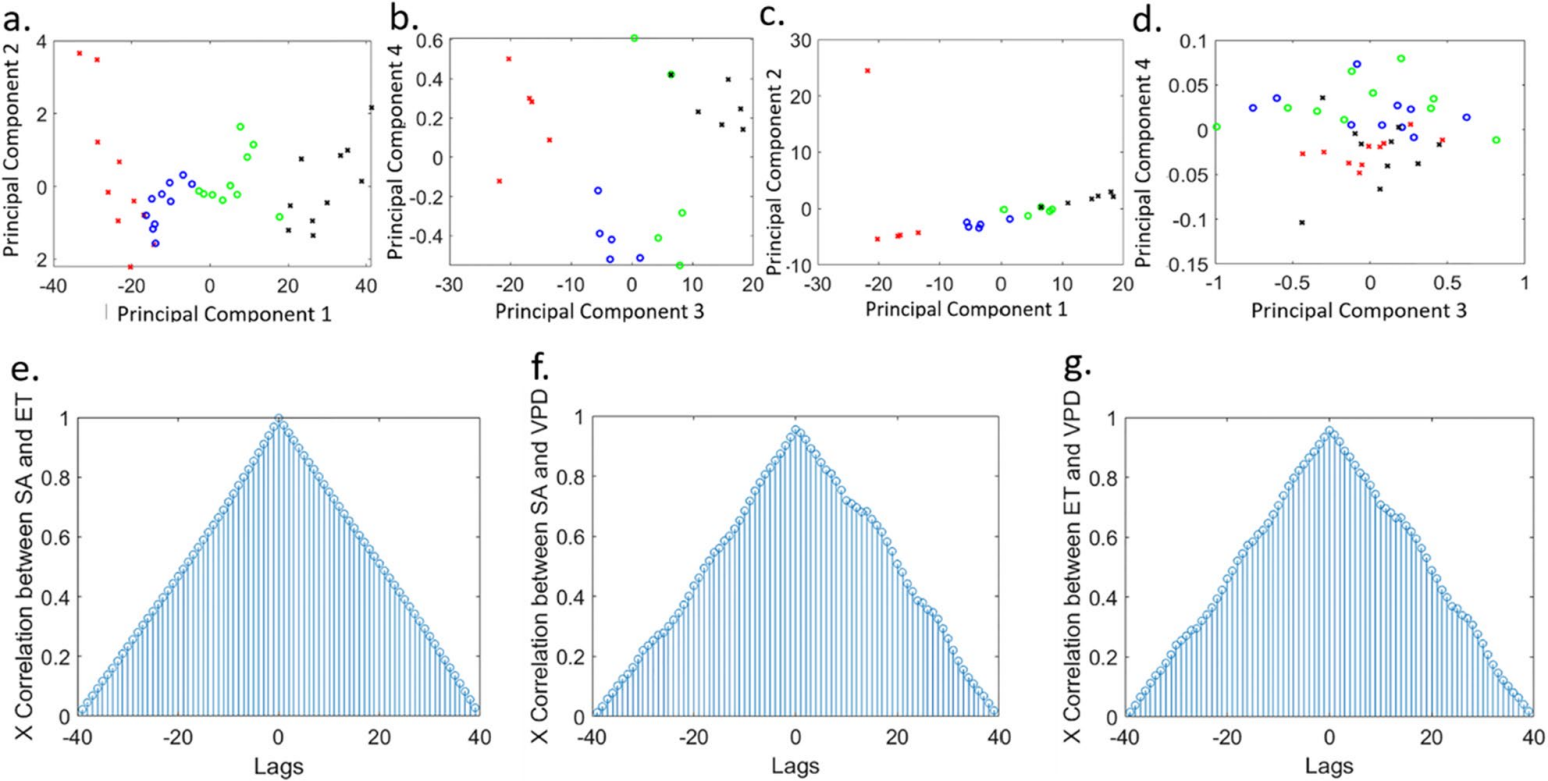
Principal component analysis for different stress stages of tomato plants: (**a**) scatter plot of principal component 1 versus principal component 2 and (**b**) scatter plot of principal component 3 versus principal component 4. Principal component analysis for different growth stages of tomato plants: (**c**) scatter plot of principal component 1 versus principal component 2 and (**d**) scatter plot of principal component 3 versus principal component 4. Normalized cross-correlation coefficients between (**e**) SA and ET, (**f**) SA and VPD, and (**g**) ET and VPD, in water-stressed bell pepper plants. Here, *ET* ethylene, *VPD* vapor pressure deficit, and ‘X’ is used as the short form for ‘cross’.

The sensor measurements were analyzed by a principal component analysis (PCA)-based pattern recognition algorithm. This analysis was conducted to gain a mathematical understanding of the capability of SA and ethylene levels in distinguishing chunks of stress levels in plants. The experiment was conducted over 40 days and every 10 days in a row was considered one stress period. Ten plants were used for this experiment. The four stress periods were defined by differing amounts of water applied to the plants. The variables of the principal component analysis were salicylic acid concentration, ethylene concentration, VPD, and stem diameter measurements over 40 days of stress stages (Fig. 6a,b) and 20 days of the growth period (Fig. 6c,d). Salicylic acid, ethylene, and stem diameter were measured once a day, while VPD was measured 4 times a day. The daily average of VPD values was computed and fed into the principal component analysis algorithm. Therefore, for stress analysis, each variable (i.e., salicylic acid, ethylene, VPD, and stem diameter) was represented as a 40 × 1 array for 40 days of measurements. On the other hand, for growth analysis, each variable (i.e., salicylic acid, ethylene, VPD, and stem diameter) was represented as a 20 × 1 array for 20 days of measurements. The variables were fed into the principal component analysis function in MATLAB, and multiple principal components were extracted. These components were then plotted to visualize the correlations present in the data.

The plot of principal component 1 versus principal component 2 showed a clear distinction between the four stress periods (Fig. 6a). However, as expected, the principal components 3 and 4 could not provide clear identifiable separation among the four stress periods (Fig. 6b). The redcross, blue circle, green circle, and black cross symbols represent 0–10 days, 11–20 days, 21–30 days, and 31–40 days of water stress, respectively.

A similar analysis was conducted at four different growth stages of tomato plants. A total of 16 plants were used for this study with 4 plants per growth stage. A noticeable separation among the growth stages was observed in the principal component 1 versus principal component 2 plot. The red cross symbols in Fig. 6c,d indicate 0–5 days of growth, blue circles represent 6–10 days of growth, green circles represent 11–15 days of growth, and black crosses represent 16–20 days of growth.

Further investigation was carried out wherein the singular value deposition and corresponding cumulative energy were analyzed (results are plotted in Fig. S24 in the supporting information). Principal components 1 and 2 were found to possess higher cumulative energy, thereby justifying their use in differentiating the stress and growth periods in plants. These results suggest that the hormone levels (SA and ethylene) could clearly distinguish different growth and stress stages in plants.

A cross-correlation analysis was performed to identify the association between the measured crop parameters (SA, ethylene, and VPD). For this study, 10 bell pepper plants were subjected to water stress. Leaf SA and ethylene levels were measured once a day (1:30 P.M.), while temperature and humidity values were recorded four times a day (8.00 a.m., 12.00 p.m., 4.00 p.m., and 8.00 p.m.), over 40 days. The normalized cross-correlation coefficients are plotted in Fig. 6e–g. A symmetrical triangular shape with respect to lag = 0 signifies a high similarity between the two parameters under consideration^72,73^. It is evident from Fig. 6e–g that the following pairs are highly similar: SA and ethylene; SA and VPD; ethylene and VPD, as is also suggested by the dynamic plots in Fig. 4g–l. The beginning of lag at -40 and ending at + 40 represents 40 days of data collection. It is noteworthy that the cross-correlation between SA and ethylene had a perfect triangular shape. In contrast, due to the oscillatory nature of VPD, the normalized cross-correlation coefficients between SA and VPD as well as ethylene and VPD showed a slightly distorted triangular shape. These results conclude the significant correlation between SA, ethylene, and VPD levels in water-stressed plants.

MATLAB codes for all the data analytics algorithms are provided in sections Code S1-S3 in the supporting information.

## Comparative analysis of sensor performance

The performance of our sensors was compared against the sensors recently reported in the literature. Table 3 below shows the comparative analysis. As can be observed, our sensors offer a wide linear operation range, low detection limit, high sensitivity, and stable performance for more than 60 days. The major features of our sensor suite include hybrid monitoring of both chemical (i.e., SA and ethylene) and physiological (i.e. temperature, humidity, and stem diameter) signaling in the plant, the capability to detect water stress/deficiency early, and ability to correlate chemical (i.e., SA and ethylene) signaling with plant water movement (i.e. VPD).

**Table 3:** Comparative Analysis.

Target	Sensing material	Sensitivity	LOD	Linear range	Stability	Reference
Salicylic acid	CB–MWCNT–Nafion/Fc	0.00107 µM^−1^	3.3 µM	25.0–1000 μM	N/A	^46^
	Ce/ZrO_2_	1013.5 µA mM^-1^ cm^−2^	1.1 µM	5.0 μM–1.0 mM	N/A	^74^
	Molecular Imprinted Polymer (MIP)/TiO_2_ nanorod/ fluorinated tin oxide	0.013 µA µM^−1^	39 nM	0.1 µM–50 µM	N/A	^75^
	Carbon electrode	29.9 nA/μM	2.54 μM	5–200 μM	N/A	^76^
Ethylene	[PdCl_2_(PhCN)_2_] and ^n^Bu_4_N[NO_2_]	1.2%/ppm	15 ppb	500 ppb–50 ppm	16 days	^77^
Temperature	Ti/Au	32 mΩ/°C	N/A	10–32 °C	151 days	^78^
	PEDOT/polyurethane	0.95%/°C	0.2 °C	20–40 °C	N/A	^79^
	Laser Induced Graphene (LIG)	5.102/°C	N/A	5–45 °C	16 days	^36^
Humidity	Laser Induced Graphene (LIG)	1.05–5.89%/RH	N/A	30–90 RH%	16 days	^36^
	Nafion/silver nanowire	0.02%/RH	2%	2–100 RH%	N/A	^69^
	Ti/Au	1.6 (a.u.)/RH	N/A	45–95 RH%	151 days	^78^
Strain	PDMS/LIG	0.448%/µm	N/A	10–50 µm diameter	11 days	^80^
	Polyvinyl alcohol/activated charcoal	1.006–2.193 (gauge factor)	N/A	0–400%	14 days	^81^
Salicylic acid	CuMOF/ CB/Nafion	7.4 × 10^−5^–0.002264 μM^−1^	0.644 µM	0.1–1000 µM	60 days	This work
Ethylene	Cu(I) complex	17.073 µA/log(ppm)	0.6089 ppm	0.1–115 ppm	60 days	
Temperature	PEDOT:PSS/GOPS	0.0098/°C	10.5478 °C	10–90 °C	> 60 days	
Humidity	fMWCNT/HEC/PVPP	0.012–0.1485 kΩ/ (%RH)	11.321RH%	10–90 RH%	> 60 days	
Pressure	Porous PDMS/CB/DES	0.016–5.04 kΩ/ kPa	0.3733 kPa	0.1–100 kPa	> 60 days	
Strain	rGO	100–900 (gauge factor)	0.1%	0.1–1.2%	> 60 days	

## Conclusion and discussion

In summary, this work presents a fully integrated sensor suite that consists of six sensors for measuring salicylic acid, gaseous ethylene, relative humidity, and temperature values of plant leaves, and combined pressure and strain sensors to quantify the radial growth of the stem. The sensor array provides a real-time evaluation of plant stress levels. A correlation study demonstrated a significant correlation between the salicylic acid, ethylene, and vapor pressure deficit measurements. Data collected from the sensor array were also fed into a principal component analysis-based pattern recognition algorithm to differentiate between different stress and growth stages of plants. The results from this research will advance crop research and production through multiple technological innovations: (1) enabling the development and expansion of the sensor design for multiplexed detection of plant’s defense-related phytohormones such as salicylic acid, indole-3-acetic acid, methyl jasmonate, abscisic acid, and volatile organic compounds (VOCs), i.e., ethylene, monoterpenes, and sesquiterpenes emitted from different parts of the plants (including leaves, roots, and stems) to provide a complete crop health diagnostic solution and (2) forming comprehensive correlations between the plant growth and environmental parameters (biotic stresses due to bacteria, virus, insect infestation, and abiotic stresses owing to drought/floods, temperature variations, soil nutrient/salinity/pH deficiencies, heavy metals toxicity, etc.). Moreover, the correlations between the hormone levels and vapor pressure deficit suggest a possible association of hormone levels with soil water content, which may substantially contribute to irrigation scheduling. Hence, the implementation of techniques from our proposed project would allow producers to receive information on real-time crop stress information and the amount of water needed to be applied to avoid over or under-irrigation.

## Supplementary Information

Supplementary Information.

## Data Availability

The datasets generated and/or analyzed during the current study are not publicly available due to a pending patent disclosure but may be available from the corresponding author upon reasonable request.
