# Supplementary material for: A hybrid multifunctional physicochemical sensor suite for continuous monitoring of crop health
Source: Sci Rep. 2023 Jun 17;13:9848. doi: 10.1038/s41598-023-37041-z (PMC10276867; doi:10.1038/s41598-023-37041-z)
Supplement: Supplementary file 1 — Supplementary Information. [file 41598_2023_37041_MOESM1_ESM.docx]

Supporting Information

A Hybrid Multifunctional Physicochemical Sensor Suite for Continuous Monitoring of Crop Health

Nafize Ishtiaque Hossain^1^ and Shawana Tabassum^1^*

^1^The University of Texas at Tyler, Tyler, TX 75799, USA

*E-mail: [stabassum@uttyler.edu](mailto:stabassum@uttyler.edu)

S1. Materials and Methods

S1.1. Chemicals and Reagents

Ammonium hydroxide, dimethylformamide (DMF), N-methyl-2-pyrrolidone (NMP), ethanol, polyvinylpyrrolidone (PVP), polyvinylpolypyrrolidone (PVPP), copper (II) nitrate tetrahydrate, copper (I) trifluoromethane sulfonate benzene complex, 2-amino terephthalic acid, carbon black, sodium borohydride, toluene, 1,2-dichlorobenzene, hydroxyethyl cellulose, poly(3,4-ethylenedioxythiophene):poly(styrenesulfonate), Nafion, fluorinated Nafion membrane, 3(glycidyloxypropyl) trimethoxysilane, benzophenone, diphenylamine, graphene ink in ethanol, silver/silver chloride (Ag/AgCl) paste, sodium hydroxide, and Terion X 100 were purchased from Sigma Aldrich. Reduced graphene oxide, single-walled carbon nanotube, and multi-walled carbon nanotube were purchased from ACS materials, while tris-HCl, sulfuric acid, and nitric acid were obtained from Fisher Scientific.

**S1.2. Instruments**

A programmable automated cutter (PrismCut, Model P20) from USCutter, Tukwila, WA, USA was used to design the sensors. An AutoCAD Fusion 360 software from AutoCAD, Autodesk Inc., San Rafael, CA, USA was used to create CAD designs. Cyclic voltammetry (CV) and differential pulse voltammetry (DPV) tests were conducted using the commercially available potentiostat EmStat (PalmSense, Houten, Netherlands). Hitachi TM 4000 plus Scanning Electron Microscope (SEM) was used to image the morphology and microstructure of coated surfaces, while Nicolet Avatar 360 E.S.P ATR-FTIR (Attenuated Total Reflectance-Fourier Transform Infrared) spectrometer was used to identify the functional groups and characterize covalent bonding formation in the coatings. Applent AT 3817 LCR meter was utilized to measure the resistance for sensors calibration. A motorized translation stage (MTS50-Z8, Thorlabs Inc., Newton, NJ, USA) and brushed DC servo motor controller (KMTS5E, Thorlabs Inc., Newton, NJ, USA) were used to conduct the stress-strain characterization of the device. To analyze the accuracy of our salicylic acid sensor, a high-performance liquid chromatography (HPLC) apparatus from Shimadzu, Kyoto, Japan was used. The HPLC consisted of a Shimadzu LC-10ATvp liquid chromatographic pump and ZIC-HILIC SeQUANT PEEK-lined stainless-steel column (250 mm × 4.6 mm, 5µm, 200Å), a Shimadzu SPD-10Aº detector at a wavelength of 201 nm, and a 7125 injector with 50-L sample loop. A mass flow controller (MFC) (EW-32658-06, Cole-Parmer, Vernon Hills, IL, USA) was used to regulate the flow of gases during the calibration and selectivity tests of the ethylene sensor.

**S1.3. Sensors Fabrication**

The hormone sensors (SA and ethylene) were comprised of three electrodes: a working electrode, a counter electrode, and a reference electrode. The temperature, humidity, strain, and pressure sensor, were each composed of two interdigitated electrodes. The conductive electrodes for SA, temperature, humidity, strain, and pressure sensors were fabricated on a flexible polyimide substrate, while the ethylene sensor was made on a flexible Nafion sheet using a screen-printing technique. Nafion was used because of its solid polymeric electrolytic performance, which is suitable for gas sensing ^[S1]^. When a conductive material (graphene in this work) is deposited over Nafion and an appropriate potential is applied across two electrodes, ionic displacement occurs through Nafion, thereby enabling electrolysis in the gas phase ^[S2]^. The thicknesses of the polyimide and Nafion sheets were 125 µm and 150 µm, respectively. Figure S1a illustrates the step-by-step fabrication procedure for the SA, temperature, humidity, pressure, and ethylene sensors and Figure S1b shows the fabrication procedure for the strain sensor. First, the two-dimensional (2D) design of the electrodes was created in AutoCAD Fusion 360 and the design was exported to the programmable cutter. Next, a double-sided tape was used to attach the Nafion sheet to the polyimide substrate (Figure S1a-i). This was done to realize all six sensors in one combined sheet. Prior to cutting, a transfer tape was attached to the polyimide and Nafion sheet areas dedicated to the SA and ethylene sensors, respectively. In contrast, the regions of the polyimide sheet that were meant for the temperature, humidity, and pressure sensors, were coated with a conductive copper (Cu) tape, as shown in Figure S1a-ii. Afterward, the combined sheet was loaded into the craft cutter. Cutting of the electrode patterns was performed in two steps. First, the PrismCut machine cut the electrode patterns for SA and ethylene sensors using a blade with a 30° angled tip at a speed of 97mm/s and a force of 4.01N to achieve optimized results. Next, the sheet was again inserted into the cutter and the cutting procedure was repeated at 4.0 N force and 100 mm/s speed to design the temperature, humidity, and pressure sensors (Figure S1a-iii). The pressure sensor was separated with a scissor followed by peeling off the Cu tape from non-electrode areas to achieve the copper-interdigitated electrodes (Figure S1a-iv). The temperature, humidity, pressure, and strain sensors, each had a dimension of 2cm x 2cm. The dimension of the salicylic acid sensor was 2cm x 5 cm and the dimension of the ethylene sensor was 4cm x 5cm.

The next steps involved screen printing of the electrodes with conductive inks. First, the transfer tape was removed from the areas dedicated to the reference electrodes of the SA and ethylene sensors (Figure S1a-iv). A squeegee was used to apply a uniform layer of Ag/AgCl paste on the exposed regions. Next, the sheet was placed inside a convection oven and heated at 80°C for 30 minutes. After annealing the Ag/AgCl paste, the sheet was removed from the oven and cooled down to room temperature. Likewise, the working and counter electrodes of the SA and ethylene sensors were screen printed with graphene ink (Figure S1a-v). The graphene-coated sheet was heated in the oven for 30 minutes at 100°C. Finally, the transfer tape was removed from the SA and ethylene sensor regions to transfer the electrodes to the polyimide and Nafion sheet, respectively. Figure S1a-vi shows the reference electrodes (RE_SA_, RE_ET_), working electrodes (WE_SA_, WE_ET_), and counter electrodes (CE_SA_, CE_ET_) of SA and ethylene (ET) sensors, and the working electrodes of relative humidity (WE_RH_) and temperature (WE_T_) sensors. It is to be noted that the strain and pressure sensors were made on opposite sides of the same piece of polyimide sheet. Figure S1b shows the fabrication process flow for the strain sensor. Briefly, the process starts by attaching a transfer tape (Figure S1b-ii) to the back side (Figure S1b-i) of the pressure sensor. Subsequently, the electrode patterns were cut on the transfer tape (Figure S1b-iii) followed by the removal of the transfer tape from the interdigitated areas (Figure S1b-iv). Finally, graphene ink was applied to the exposed interdigitated patterns (Figure S1b-v) and the remaining transfer tape was removed from the polyimide sheet (Figure S1b-vi).


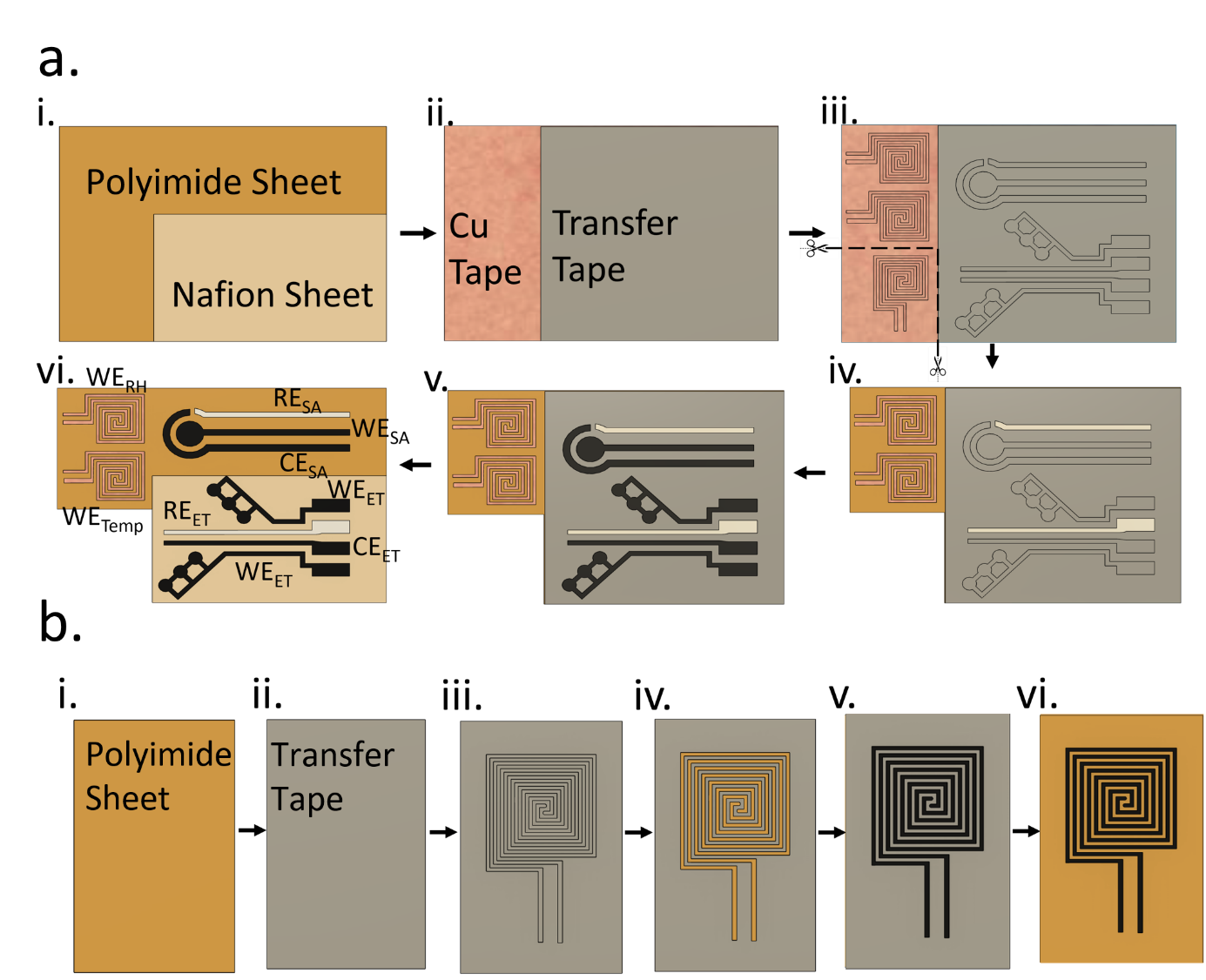


**Figure S1.** (a) Step-by-step demonstration of fabricating the integrated sensor suite: (i) Nafion sheet attached to the polyimide sheet, (ii) transfer tape and copper (Cu) tape applied to the respective regions, (iii) electrode designs transferred to the sheet by the craft cutter, (iv) the pressure sensor was removed by a scissor, undesired portions of the Cu tape were peeled off, and the reference electrode regions of the SA and ethylene sensors were coated with Ag/AgCl paste, (v) counter and working electrodes of the SA and ethylene sensors were coated with graphene paste, and (vi) the transfer tape was peeled off to transfer the electrodes to the polyimide and Nafion sheets. (b) Step-by-step illustration of the fabrication of the strain sensor: (i) the back side of the pressure sensor (which was removed from the combined sheet in steps 1a-iii, iv), (ii) polyimide sheet was covered by the transfer tape, (iii) electrode patterns of the strain sensor were transferred, (iv) the transfer tape was removed from interdigitated electrode areas, (v) the interdigitated electrode patterns were coated with graphene ink, and (vi) the transfer tape was peeled off from the remaining areas of the polyimide sheet.

**S1.4. Surface Functionalization**

S1.4.1. A composite coating of copper metal-organic framework-carbon black-Nafion for Salicylic Acid sensing

The synthesis of copper-based metal-organic framework (CuMOF) is reported in our previous work ^[S3]^. Briefly, 8 mL of dimethylformamide (DMF) and 8 mL of ethanol were mixed and centrifuged at 300 rpm for 15 minutes followed by ultrasonication for 30 minutes. Afterward, 0.4g of polyvinyl pyrrolidone (PVP) was added to the mixture and sonicated for 30 minutes. Next, 46.64 mg of copper (II) nitrate tetrahydrate was separately mixed with 10.86 mg of 2-aminoterephthalic acid in 4 mL of DMF and ultrasonicated for 30 minutes. The resulting solutions were mixed and ultrasonicated for 40 minutes. The mixture was then heated in a convection oven at 100°C for 5 hours. The resulting green precipitate was collected and subsequently dissolved in 40 mL of DMF. Next, this solution was centrifuged at 1000 rpm for 30 minutes and a green CuMOF precipitated at the bottom. Finally, the supernatant DMF was removed and the CuMOF precipitate was dried at 50°C in the oven. To obtain the optimized coating for SA, five different weight ratios (1:3, 1:2, 1:1, 2:1, 3:1) (w/w) of CuMOF and carbon black (CB) were dissolved in DI water by ultrasonication for 30 minutes. Next, 5 µL of Nafion (w/v) (0.01%) was added to the resulting solutions and ultrasonicated for 30 minutes to obtain a composite of CuMOFs-CB-nafion. Finally, 10 µL of the composite solution was drop cast on the working electrode (WE_SA_) of the SA sensor. The 2:1 weight ratio of CuMOF and CB provided the highest sensitivity and current ratio in response to SA level variations and the lowest limit of detection (LOD), as demonstrated in Figure S2 and Table S1. Hence, all the subsequent measurements were carried out with a 2:1 weight ratio of CuMOF to CB.


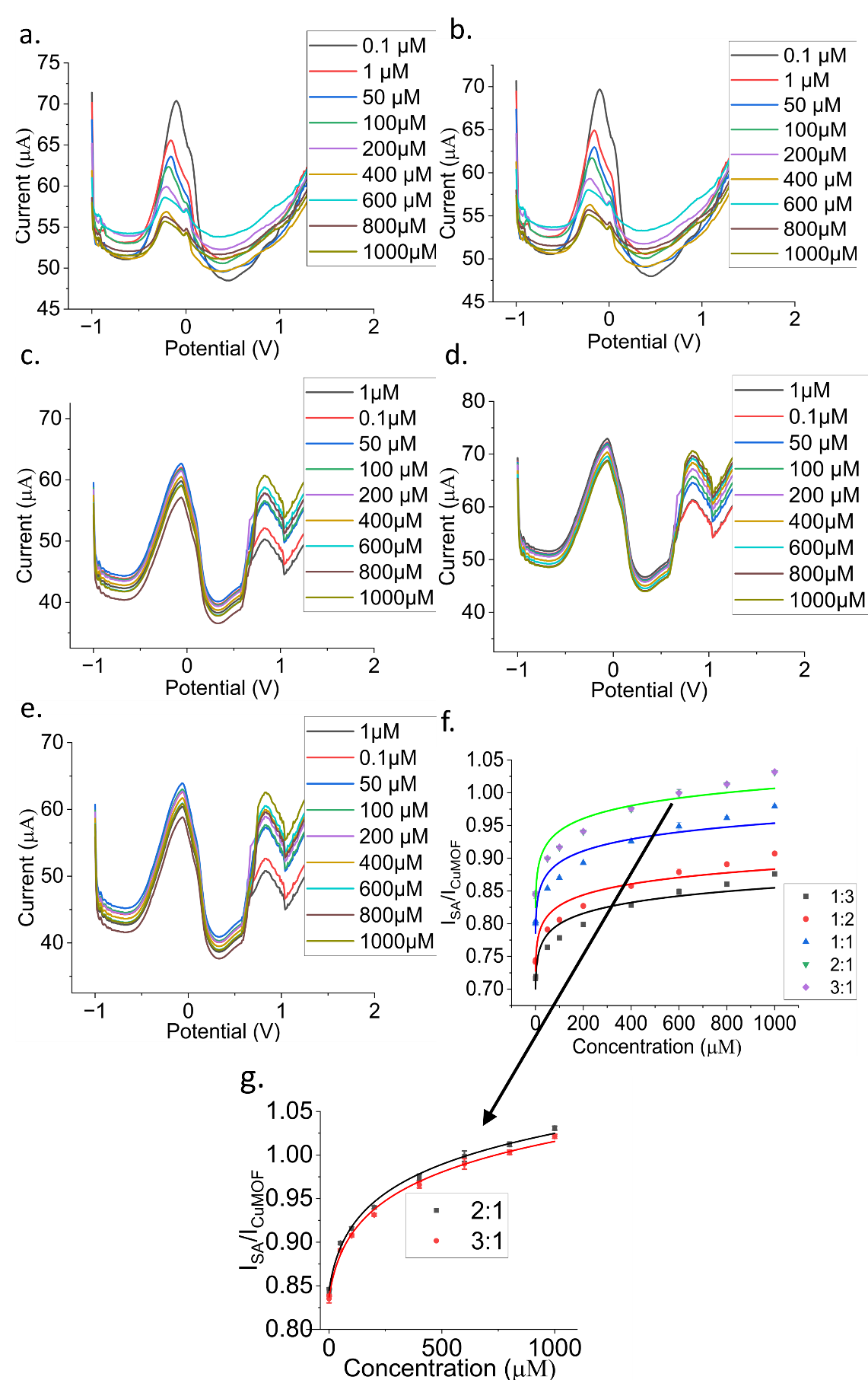


**Figure S2.** Differential Pulse Voltammetry (DPV) plots for SA levels ranging from 1 µM to 1000 µM when the working electrode, WE_SA_, was coated with (a) 1:3, (b) 1:2, (c) 1:1, (d) 2:1, and (e) 3:1, weight ratios of CuMOF and CB. (f) Calibration curves of the SA sensor for five different weight ratios of CuMOF to CB (i.e., 1:3, 1:2, 1:1, 2:1, and 3:1). (g) The calibration curves obtained with 2:1 and 3:1 weight ratios of CuMOF and CB are zoomed in to show the slight differences between the two plots.

**Table S1.** Sensitivity and LOD analysis of the SA sensor for different weight ratios of CuMOF and CB.

| CuMOF:CB | Low Conc. Sensitivity | High Conc. Sensitivity | Limit of Detection (LOD) |
| --- | --- | --- | --- |
| 1:3 | 0.002216 μM^-1^ | 7.32589 X 10^-5^ μM^-1^ | 0.6977 μM |
| 1:2  1:1  2:1  3:1 | 0.002115 μM^-1^  0.002233 μM^-1^  0.002264 μM^-1^  0.002198 μM^-1^ | 7.3158 X 10^-5^ μM^-1^  7.3894 X 10^-5^ μM^-1^  7.409 X 10^-5^ μM^-1^  7.3968 X 10^-5^ μM^-1^ | 0.678 μM  0..687 μM  0.664 μM  0.7005 μM |

S1.4.2. A composite copper complex (I)-single-walled carbon nanotube coating for ethylene sensing

The recipe in our earlier work ^[S4]^ was followed to prepare the copper complex (I) solution. At first, 0.4g of NaBH_4_ and 7.55g of [3,5-(CF_3_)_2_-pyrazol-1-yl] (also known as 3,5-(CF_3_)_2-_pz) were mixed with kerosene in a conical flux to form a homogeneous mixture. The solution was slowly heated to 190°C with a 1°C/min ramp and kept at that temperature for 4 hours. The flux was partially submerged in silicone oil during the heating process. The solution was occasionally (every 15 minutes) heated with a heat gun until pyrazole melted. Next, the solution was cooled down to room temperature and diethyl ether was added to filter out the excess reagents. The resulting white solid was characterized with nuclear magnetic resonance (NMR) imaging to confirm the formation of the product Na[HB(3,5-(CF_3_)_2_-pz)_3_]. Next, 8 mg of copper(I) trifluoromethane sulfonate benzene complex was dissolved in 3 mL dry, degassed toluene. Subsequently, 17 mg of Na[HB(3,5-(CF_3_)_2_-pz)_3_] was introduced and the resulting mixture was stirred for 20 hours at room temperature. The solution was filtrated through a Whatman 0.02 *μ*m syringe filter and a colorless solution of copper complex-1 was obtained.

In a separate tube, 0.5 mg of single-walled carbon nanotube (SWCNT) was added to a mixture of 0.8 mL 1,2-dichlorobenzene and 1.16 mL toluene, and the resulting mixture was sonicated for 2 hours to prepare a homogeneous solution. Next, the freshly prepared copper complex-1 solution was added to this mixture and sonicated for another 1 hour. Finally, 30 µL of this solution was drop cast on the working electrode (WE_ET_) of the ethylene sensor.

S1.4.3. Functionalized multiwalled carbon nanotube-hydroxyethyl cellulose coating for relative humidity sensing

The selective coating for the humidity sensor was composed of functionalized multiwalled carbon nanotube (f-MWCNT) and hydroxyethyl cellulose (HEC). Modification of MWCNT was done to increase its hydrophilicity and prepare a homogeneous dispersion in an aqueous solution ^[S5, S6]^. Therefore, the MWCNT was functionalized with hydroxyl (-OH) groups using acid treatment. At first 200 mg of MWCNT was added to a 3:1(v/v%) mixture of sulfuric acid and nitric acid. Next, this mixture was stirred at 500 rpm for 3 hours while applying 140°C reflux simultaneously. Afterward, both the reflux and stirring were stopped and the mixture was left to cool down to room temperature. Ammonium hydroxide was added until the pH of the solution reached 5.5. Next, the f-MWCNT was vacuum filtered using a 0.2 µm PTFE membrane and then heated in the convection oven at 140°C for 12 hours. Following this step, a 1.2 wt% of f-MWCNT dispersion was made in DI water. Magnetic stirring was performed continuously for 3 hours to prevent the evaporation of DI water ^[S7]^. Consequently, four different weight ratios of f-MWCNT and HEC (1:6, 1:4, 1:2, 1:1) were prepared and stirred for 3 hours. It is noteworthy that HEC is hygroscopic and hence has a high solubility in water, thereby preventing the agglomeration of f-MWCNT ^[S8]^. The mixture containing f-MWCNT and HEC was constantly stirred for 30 hours to prepare a homogeneous solution. Next, 50 wt% of polyvinylpolypyrrolidone (PVPP) was added with a 1:2 binder to filler ratio and the resulting solution was stirred for 5 hours to achieve homogeneity. This ink was evenly applied to the interdigitated electrodes to realize a humidity sensor ^[S9]^. The f-MWCNT to HEC ratio of 1:6 (with the length of f-MWCNT being 10nm) provided the highest sensitivity in response to variations in relative humidity levels and the lowest LOD (see Figure S3 and Table S2). Hence, all the subsequent measurements were carried out with a 1:6 weight ratio of f-MWCNT to HEC.

S1.4.4. PEDOT:PSS for Temperature Sensing

Poly(3,4-ethylenedioxythiophene): poly(styrenesulfonate) (PEDOT:PSS) was used as the selective coating for the temperature sensor. The process to synthesize this coating is described in our earlier work ^[S10]^. Briefly, 1.3 wt% of 100 mg PEDOT:PSS was mixed with 50 mg Trion X-100 surfactant, and the mixture was centrifuged at 300 rpm for 10 minutes. Different amounts of the 3(glycidyloxypropyl)trimethoxysilane (GOPS) cross-linker were added to this mixture. The weight ratio of PEDOT:PSS to GOPS was varied (1:1, 1:3, 1:5, 1:7, 1:9, and 1:11). The mixture was centrifuged at 400 rpm for 30 minutes to achieve uniformity and then degassed at a desiccator for 30 minutes to remove air bubbles from the mixture. Finally, 300 µL of this solution was drop cast over the interdigitated electrode of the temperature sensor and subsequently annealed at 140°C for 60 minutes so that the solvent was entirely evaporated, and the cross-linking was complete. It was observed that the PEDOT: PSS to GOPS ratio of 1:9 provided the highest sensitivity to temperature variations (Figure S4). Additionally, to protect PEDOT:PSS from moisture and light, Kapton tapes of different thicknesses (1⁄2 mil, 1 mil, and 2 mil) were used to encapsulate the PEDOT:PSS coating. Our experiments showed that the 2 mil Kapton tape resulted in the best performance (Figure S5). Light, particularly UV light, has an impact on the conductivity of PEDOT:PSS coating. As sunlight contains a considerable amount of UV radiation, measures need to be taken to mitigate this artifact, along with the humidity artifact. To address this issue, the PEDOT:PSS/GOPS coating was shielded with Kapton tape, which possesses the ability to block UV light ^[S11]^.

**S1.4.5. A porous PDMS framework for pressure sensing**

Benzophenone and diphenylamine were mixed at a molar ratio of 1:1 to form a deep eutectic solvent (DES) ^[S12].^ The eutectic solution had a yellowish color due to charge transfer interactions. Subsequently, a fixed amount of CB was introduced to the yellow DES, which formed a uniform gel owing to the van der walls and π- π interactions between DES and CB. Separately, polydimethylsiloxane (PDMS) polymer was prepared using the standard mixing ratio of 10-parts base elastomer and 1-part curing agent. Then the as-prepared PDMS was added to the DES-CB gel to get a slurry-like ink. Different weight ratios of 1:1:0.02, 1:0.5:0.02, and 1:0:0.04 were experimented with to find the optimized PDMS:DES:CB (Figure S6). It was observed that PDMS:DES:CB = 1:1:0.02 resulted in the highest sensitivity to pressure changes. Hence this weight ratio was used for subsequent analysis. This composite ink was drop cast over the interdigitated electrodes of the pressure sensor followed by a two-step curing process. The ink was annealed at 75°C to cure the PDMS followed by final annealing at 140°C to remove the DES. Phase separation between the DES and PDMS occurred due to their immiscibility. During the 75°C annealing step, the boiling point of DES was higher than the curing temperature of PDMS. As a result, DES remained as a liquid template in the casted film. When the layer was heated to 140°C, DES was evaporated, leaving behind a porous structure in the resulting composite film ^[S13]^. Such a porous network offered superior performance in response to applied pressure variations.

**S1.4.6. Reduced graphene oxide coating for strain sensing**

The strain sensor was made of a reduced graphene oxide (rGO) dispersion. rGO was added to N-methyl-2-pyrrolidone (NMP) and sonicated for 1 hour to prepare a 2mg/mL solution. Subsequently, 50 µL of this uniform dispersion was drop cast over the graphene-interdigitated electrode ^[S14]^. Reduced graphene oxide is sensitive to water vapor. Therefore, the rGO-coated electrode was encapsulated with different thicknesses of Kapton tape (1⁄2 mil, 1 mil, and 2 mils). The 2 mil Kapton tape demonstrated optimized sensor performance.

**S1.5. Gas Sensing Setup for Characterization Studies**

The ethylene gas sensor was calibrated by flowing a series of known concentrations of ethylene gas (0.1 ppm, 1 ppm, 10 ppm, 30 ppm, 50 ppm, 75 ppm, and 115 ppm), pre-diluted with nitrogen, into a sensing chamber containing the sensor. The volume of the sensing chamber was 12in x 12in x 8in = 30.48cm x 30.48cm x 20.32cm = 18877.8977cm^3^.

The flow rate of each gas was regulated by a mass flow controller (MFC) (EW-32658-06, Cole-Parmer, Vernon Hills, IL, USA). Within the enclosed chamber, a constant flow rate of dry nitrogen at 10 cm^3^/s was maintained, while the flow rate of ethylene was adjusted using the MFC to achieve different concentrations. To prevent any potential interference caused by moisture, dry nitrogen was employed to eliminate moisture from the chamber before exposing the sensor to each ethylene concentration.

The same testing setup was used for the selectivity test. In that case, multiple gas flow controllers were used simultaneously to control the flow rate of the gases in order to produce a gas mixture inside the sensing chamber.


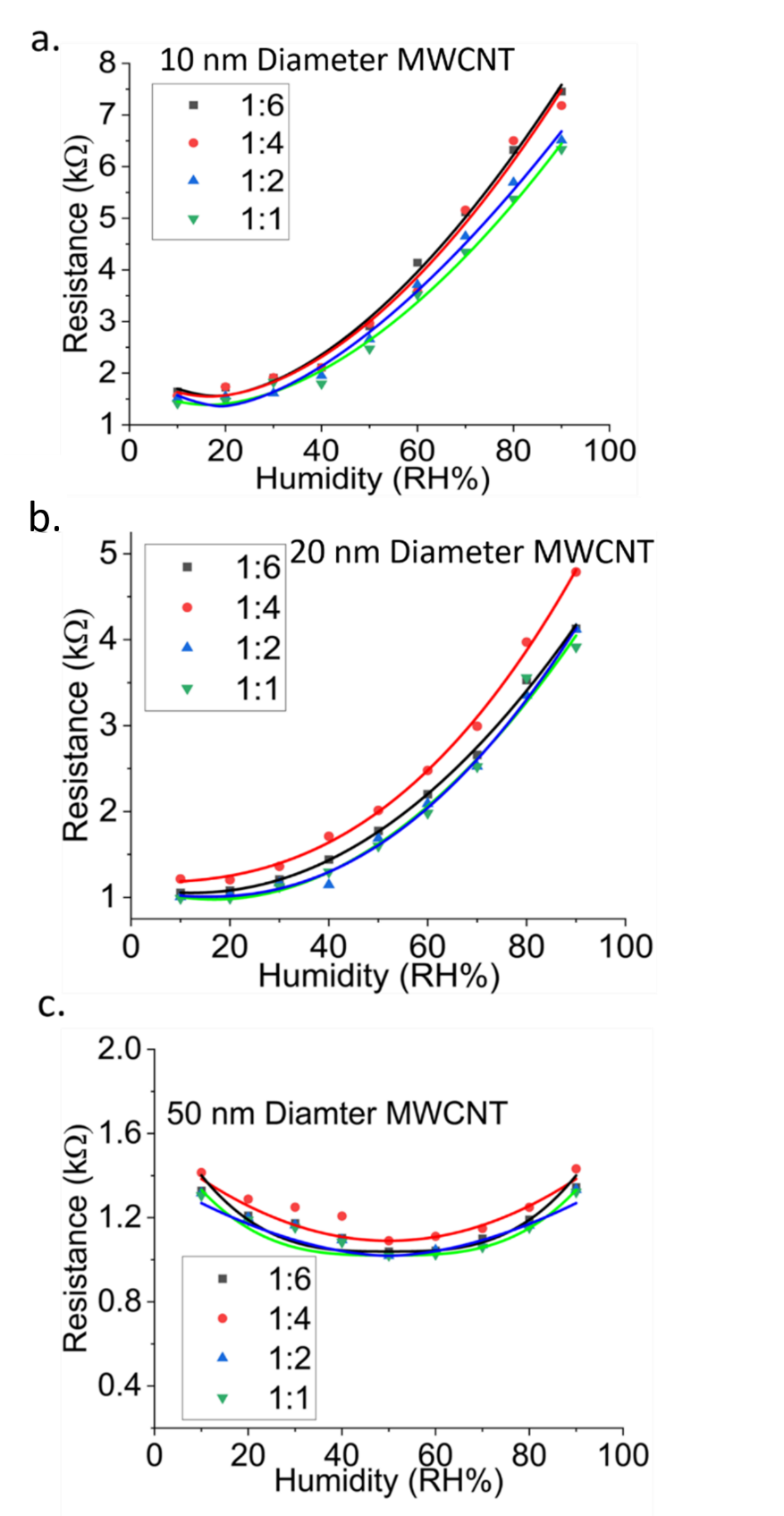


**Figure S3.** Calibration curves of the humidity sensor for different weight ratios of MWCNT to HEC (1:1, 1:2, 1:4, and 1:6) with the lengths of MWCNT being (a) 10 nm, (b) 20 nm, and (c) 50 nm.

**Table S2.** Sensitivity and LOD analysis of the different ratio of f-MWCNT:HEC

| f-MWCNT size | Ratio  (f-MWCNT:HEC) | Low-RH Sensitivity  kΩ/(%RH) | High-RH Sensitivity  kΩ/(%RH) | Limit of Detection (LOD) |
| --- | --- | --- | --- | --- |
| 10 nm | 1:6  1:4  1:2  1:1 | 0.011589  0.011089  0.001056  0.0010158 | 0.1485  0.1324  0.1245  0.1215 | 11.321 %RH  11.541 %RH  11.856 %RH  12.057 %RH |
| 20nm  50nm (fitted with polynomial) | 1:6  1:4  1:2  1:1  1:6  1:4  1:2:  1:1 | 0.011223  0.011258  0.010876  0.010681  0.0004  0.0003  0.0002  0.0001 | 0.1365  0.1408  0.1376  0.1357  0.005  0.0052  0.0025  0.0011 | 11.258 %RH  11.311 %RH  11.452 %RH  11.523 %RH  41.732 %RH  40.365 %RH  45.258 %RH  49.568 %RH |





**Figure S4.**  Calibration curves of the temperature sensor for different weight ratios of PEDOT:PSS to GOPS i.e., 1:1(G1), 1:3(G3), 1:5(G5), 1:7(G7), 1:9(G9) and 1:11(G11). Sensitivity with G1, G3, G5, G7, G9, and G11 was found to be 0.0702, 0.0703, 0.0759, 0.0802, 0.098, and 0.0853 kΩ/°C, respectively.





**Figure S5.**  Calibration curves of the temperature sensor covered with different thicknesses of Kapton tape: 0.5 mils, 1 mil, and 2 mils.





**Figure S6.**  Calibration curves of the pressure sensor for different weight ratios of PDMS:DES:CB (1:1:0.02, 1:0.5:0.02, and 1:0:0.04).

**S2. Sensing Mechanism**

**S2.1. Salicylic Acid sensor**

The sensing mechanism relied on electrochemistry wherein the redox reaction of SA on the chemically functionalized working electrode (WE_SA_) was translated to a current flow proportional to the hormone concentration. The sensing mechanism of the electrochemical SA sensor is illustrated in Figure S7a. The working electrode was coated with a CuMOF:CB: Nafion layer selective to SA. The data logger (commercial EmStat potentiostat) sent a staircase voltage pulse between the working (WE_SA_) and reference electrodes (RE_SA_) of the sensor. As a result, SA was oxidized on the working electrode and formed SA^+^, while the CuMOF was reduced. This redox reaction generated a current flow between the working (WE_SA_) and counter (CE_SA_) electrodes, recorded and analyzed by the data logger. A ratiometric approach was used to compute the response signal from the ratio of SA and CuMOF oxidation peak currents (more details in our prior work ^[S3]^). The current ratio was correlated to the concentration of SA oxidized at the WE_SA_ surface.

S2.2. Ethylene sensor

The ethylene sensor was made on a thin Nafion sheet. A triangular voltage waveform was applied between the working (WE_ET_) and reference (RE_ET_) electrodes by our data acquisition and processing (DAP) module. Upon exposure to gaseous ethylene, the active sites in the composite layer of copper complex and SWCNT were blocked by ethylene, which resulted in a decrease in the current measured between WE_ET_ and CE_ET_. The Nafion sheet enabled electrolysis in the gas phase without the need for any buffer solution (Figure S7b).

S2.3. Temperature, humidity, pressure, and strain sensors

The temperature, humidity, pressure, and strain sensors were each made of two interdigitated electrodes. The dimension of the interdigitated electrode was the same for all the sensors. The impedance of the electrode was a function of electrode separation, electrode length, width, and the coating property ^[S15]^. The resistance of the selective coating changed in response to variations in temperature/RH/pressure/strain (Figure S7c). An Applent LCR meter (AT 3817A) was used to calibrate the sensors. However, to make the system wearable and collect in situ measurements from plants, the sensor was connected in series with a known resistor to form a voltage divider circuit, as also explained in the main article. As a result, the resistance variations were converted into voltage signals that were detected by a microcontroller. Figure S7c shows the voltage divider circuit where R_known_ represents a known resistance and R_sensor_ is the sensor resistance. V_i_ represents the supply voltage of 3.3V and V_0_ is the voltage measured across the temperature/RH/pressure/strain sensor. The auto-ranging functionality was adopted to select R_known_ from some previously known resistor values. The resistance of the sensor, R_sensor_, was measured using the following voltage divider formula:

R_sensor_ = (R_known_) /((V_i_/V_0_) -1) (S1)


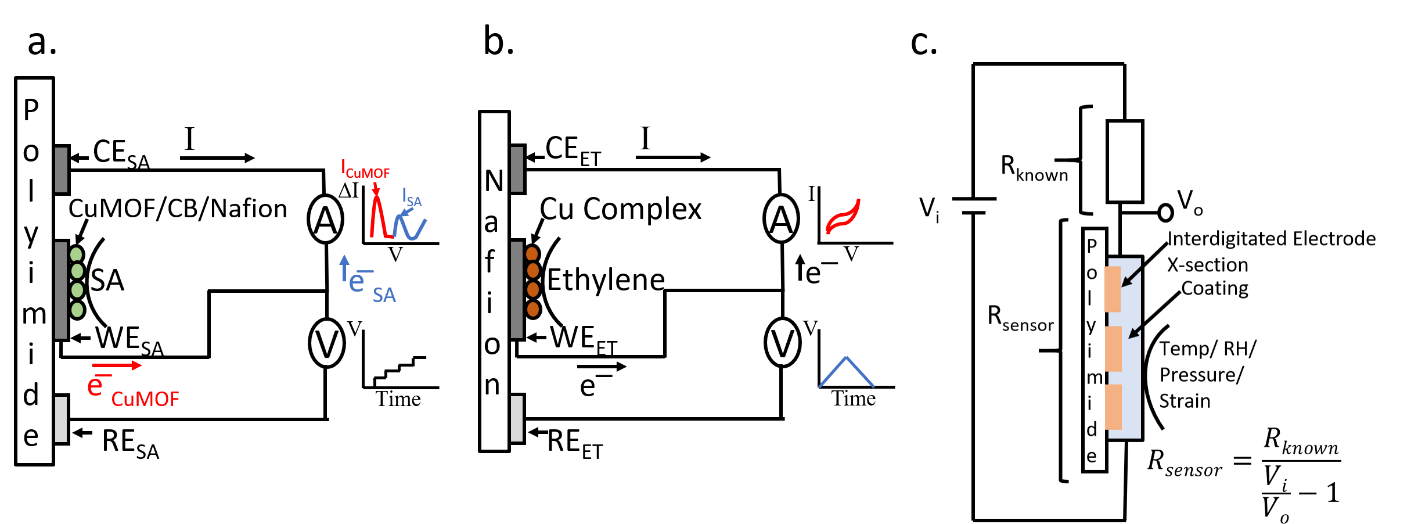


**Figure S7.** Working principle of (a) SA sensor coated with CuMOF:CB:Nafion, (b) ethylene sensor coated with a Cu complex selective layer, and (c) temperature, humidity, pressure, and strain sensors, each coated with the respective selective layer.

**S3. Spectroscopic and Electron Microscopic Analysis**

The CuMOF coating was characterized by Fourier Transform Infrared (FTIR) spectroscopy and scanning electron microscopy (SEM). The FTIR spectrum in Figure S8a shows that the peaks at 3550 cm^-1^ and 3390 cm^-1^ were due to the asymmetric and symmetric stretching vibrations of -NH_2_, confirming the presence of amino groups in CuMOF. The stretching vibration peak that appeared at 2950 cm^-1^ for -OH was mitigated by the coordination interactions between Cu^2+^ ion and -COOH of 2-aminoterephthalic acid. The FTIR spectra of CuMOF agreed with the previous reports ^[S16]^. The morphology of the as-prepared CuMOF-CB-Nafion coating was investigated by SEM (Figure S8b). The obtained coating displayed a layer-by-layer structure with a wrinkled surface, indicating the large surface area of the CuMOF-CB-Nafion nanosheets.

One crucial step in the synthesis of the copper complex (I) coating was the accurate formation of the intermediate product Na[HB(3,5-(CF_3_)_2_-pz)_3_]. Hence, the formation of this intermediate product was verified through NMR spectroscopy, as depicted in Figure S8c. The spectroscopic results confirmed the presence of the product Na[HB(3,5-(CF_3_)_2_-pz)_3_]. Furthermore, the SEM image demonstrated the size, distribution, and morphology of the copper complex (I) nanoparticles deposited on the graphene-coated electrode surface (Figure S8d). The nanoparticles had a mean diameter of about 10 nm.

The f-MWCNT was characterized with FTIR (Figure S8e). The absorption peaks between 3500 cm^-1^ and 4000 cm^-1^ were due to the presence of -OH functional groups. The peaks located between 2975-3065cm^-1^, 1390-1400cm^-1,^ and 1100-1160cm^-1^ confirmed the presence of C-H bonds. The unexpected peaks that occurred between 1950 to 2510 cm^-1^ represented the artifacts of the diamond ATR setup. The FTIR spectra of the f-MWCNT coincide with the previous reports ^[S17]^. The SEM image in Figure S8f shows the size, distribution, and morphology of the HEC/MWCNT network. It is evident from the SEM image that a homogeneous and uniform MWCNT mesh was obtained.

Figure S8g shows the SEM image of the porous PDMS network formed by first trapping and then evaporating the deep eutectic solvent. The phase segregation and porous structure were observed. The pores had diameters ranging from 10 μm to 400 μM. Finally, Figure S8h shows the SEM image of reduced graphene oxide (rGO) bundles, which appeared as randomly aggregated thin sheets, with distinct edges, wrinkled surfaces, and folding.


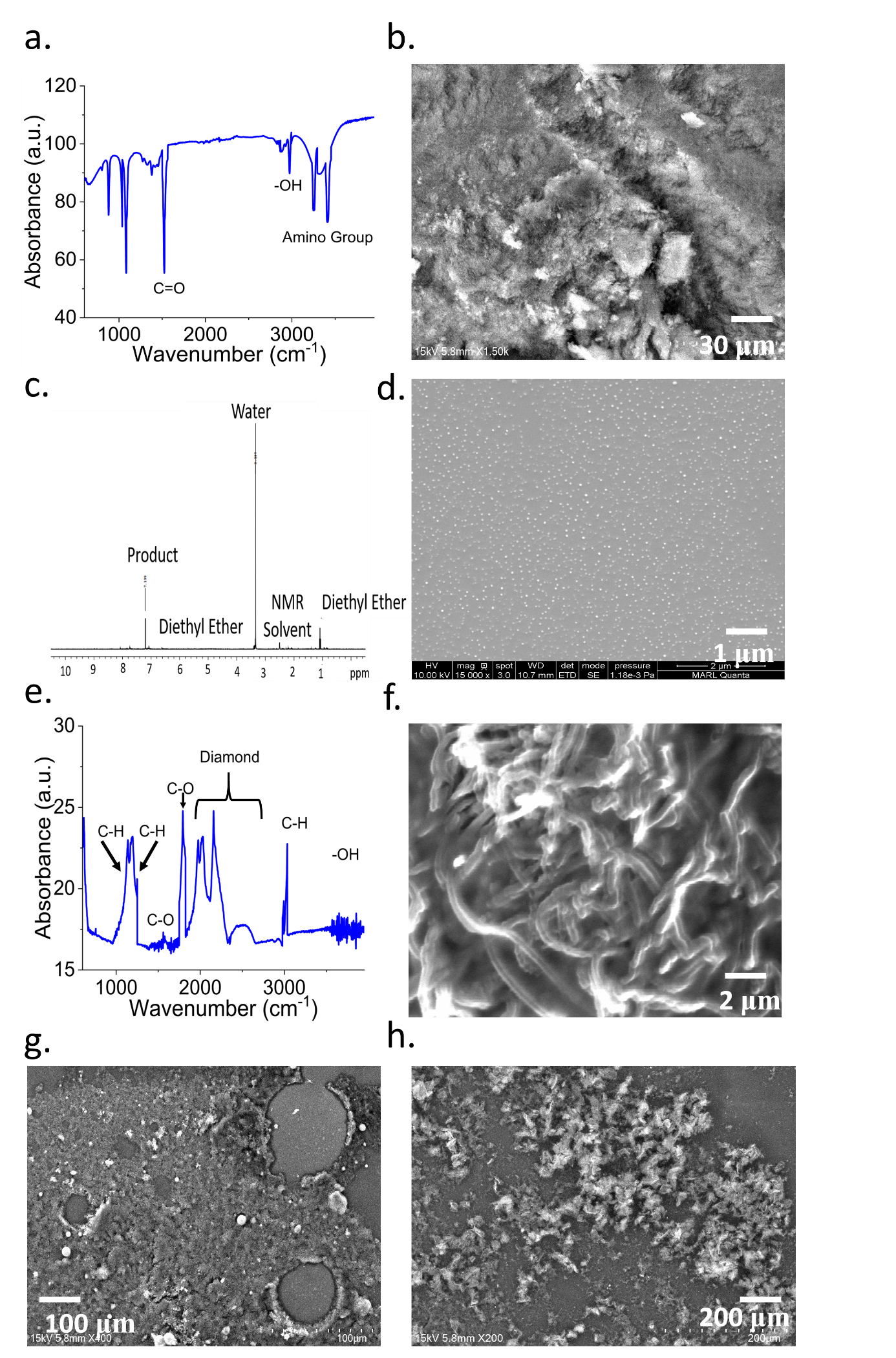


**Figure S8.** (a) FTIR spectrum showing the functional groups present in CuMOF. (b) SEM image showing the morphology of the CuMOF/CB/Nafion coating spread over the SA working electrode. (c) NMR spectroscopy of the ether mixture of Na[3,5-(CF_3_)_2_-pz]. (d) SEM image of copper complex (I) nanoparticles over the working electrode of the ethylene sensor. (e) FTIR characterization of functionalized MWCNT. (f) SEM image shows the morphology of the HEC/MWCNT/PVPP coating. (g) SEM image of the porous PDMS indicating the formation of pores due to the evaporation of DES. (h) SEM image of rGO.


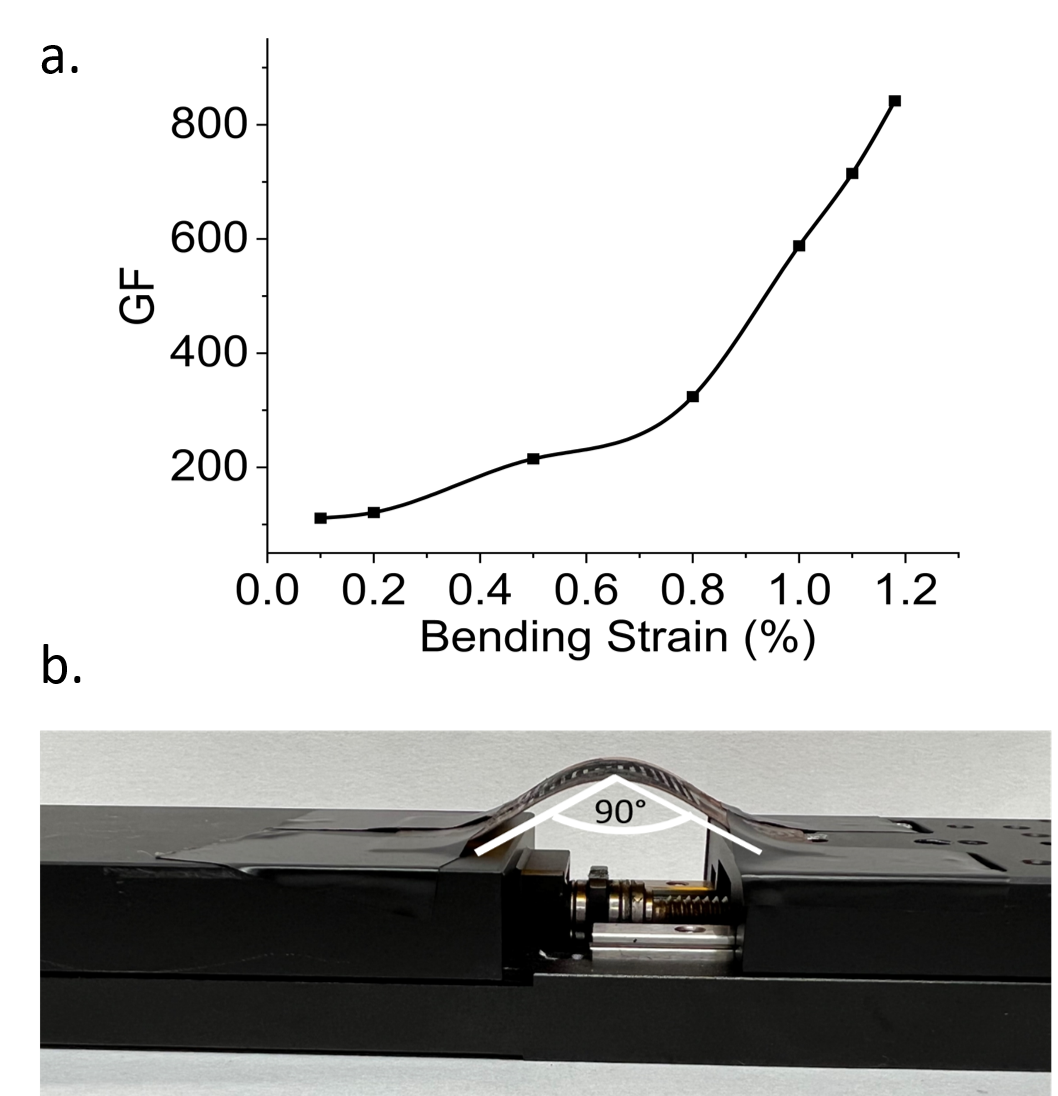


**Figure S9.** (a) Gauge Factor (GF) versus bending strain. (b) Motorized base set-up to measure the bending radius of the strain sensor.

**S4. Calculation of Sensor Resolution**

We used an iterative approach to calculate the resolution of the sensors. The target (i.e., salicylic acid and ethylene concentrations, relative humidity, temperature, and strain values) was varied until a detectable change in the sensor response was observed. Simultaneously, the measured response was compared against the response calculated from the calibration curves shown in Figure 2 in the manuscript.

**Calculation of Resolution for the Strain sensor:**

The LOD of the strain sensor is found to be 9.3211° from Equation (4) in the manuscript.

The resistance value at 9.3211° bending angle was measured as

R = 18260.062 Ω

From the calibration equation as listed in Table I of the manuscript:

$R=0.00248\left( \theta\right)^{1.442}+18260= 0.00248\left( 9.3211 \right)^{1.442}+18260= 18260.062 \Omega$

The value calculated from the calibration equation is the same as the measured value.

The resistance value at 9.3411° bending angle was measured as

R = 18260.062 Ω

From the calibration equation, $R=0.00248\left( 9.3411 \right)^{1.442}+18260= 18260.062 \Omega$

No detectable change is observed in the R values measured for 9.3211° and 9.3411° bending angles.

The resistance value at 9.3611° bending angle was measured as

R = 18260.062 Ω

From the calibration equation, $R=0.00248\left( 9.3611 \right)^{1.442}+18260= 18260.062 \Omega$

No detectable change is observed in the R values measured for 9.3211° and 9.3611° bending angles.

The resistance value at 9.3811° bending angle was measured as

R = 18260.063 Ω

From the calibration equation, $R=0.00248\left( 9.3811 \right)^{1.442}+18260= 18260.0625=\sim18260.063 \Omega$

The difference between measured R in response to 9.3811° and 9.3211° is distinguishable. Moreover, the value calculated from the calibration equation matches with the measured value.

Hence, we conclude that the minimum change in the bending angle that our strain sensor can detect is = 9.3811° - 9.3211° = 0.06°.

As the strain sensor can detect 0.06° increment of angle of curvature, this is the resolution of the developed strain sensor.

**Calculation of Resolution for the SA sensor:**

The identical sensor was employed for all calculations. Three repetitions of measurements were conducted at each concentration, and the reported current values represent the average of three consecutive measurements.

The LOD of the SA sensor is found to be 0.644 μM from Equations (5-7) in the manuscript.

The current responses at 0.644 μM (~0.65 μM) of SA were measured as

I_SA_ = 63.81 μA

I_CuMOF_ = 75.36 μA.

The ratio, I_SA_/I_CuMOF_ = 63.81/75.36 = 0.8467

From the calibration equation as listed in Table I of the manuscript:

$$\frac{I_{\mathrm{SA}}}{I_{\mathrm{CuMOF}}}=0.0143 {(SA)}^{0.3787}+0.8346=0.0143{(0.644)}^{0.3787}+0.8346=0.8467$$

The value calculated from the calibration equation matches with the measured value.

We continued this process for every 0.1 μM increment in SA concentration until a detectable change in the sensor response was observed and the measured response matched with the response calculated from the calibration.

The sensor response at 0.75 μM was

I_SA_ = 63.84 μA

I_CuMOF_ = 75.4 μA.

The ratio, I_SA_/I_CuMOF_ = 63.84/75.4 = 0.8467

From the calibration equation:

$$\frac{I_{\mathrm{SA}}}{I_{\mathrm{CuMOF}}}=0.0143 {(SA)}^{0.3787}+0.8346=0.0143{(0.75)}^{0.3787}+0.8346=0.8474$$

The value calculated from the calibration equation does not match with the measured value.

The sensor response at 0.85 μM was

I_SA_ = 63.85 μA

I_CuMOF_ = 75.41 μA.

The ratio, I_SA_/I_CuMOF_ = 63.85/75.41 = 0.8467

From the calibration equation:

$$\frac{I_{\mathrm{SA}}}{I_{\mathrm{CuMOF}}}=0.0143 {(SA)}^{0.3787}+0.8346=0.0143{(0.85)}^{0.3787}+0.8346=0.8480$$

The value calculated from the calibration equation does not match with the measured value.

The sensor response at 0.95 μM was

I_SA_ = 63.967 μA

I_CuMOF_ = 75.381 μA.

The ratio, I_SA_/I_CuMOF_ = 63.967/75.381 = 0.8486

From the calibration equation:

$$\frac{I_{\mathrm{SA}}}{I_{\mathrm{CuMOF}}}=0.0143 {(SA)}^{0.3787}+0.8346=0.0143{(0.95)}^{0.3787}+0.8346=0.8486$$

The value calculated from the calibration equation matches with the measured value. Moreover, the difference between measured I_SA_/I_CuMOF_ in response to 0.95 μM and 0.65 μM is distinguishable.

Hence, we conclude that the minimum change in the SA concentration that our sensor can detect is = 0.95 - 0.65 = 0.3 μM.

A similar approach was used to calculate the resolution of other sensors.


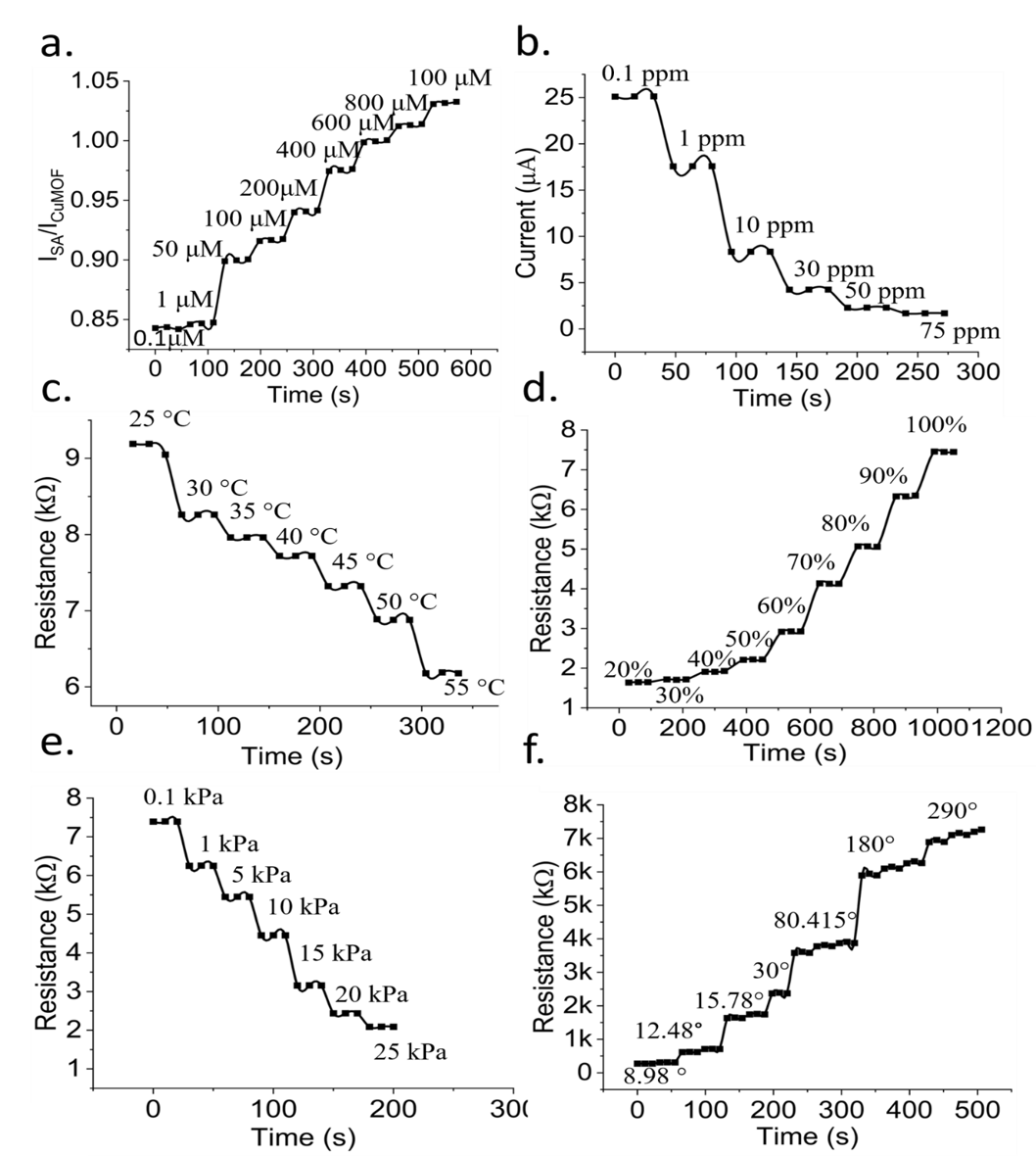


**Figure S10.** Dynamic response of (a) SA sensor, (b) ET sensor, (c) Temperature sensor, (d) Relative humidity Sensor, (e) Pressure Sensor, and (f) Strain sensor.


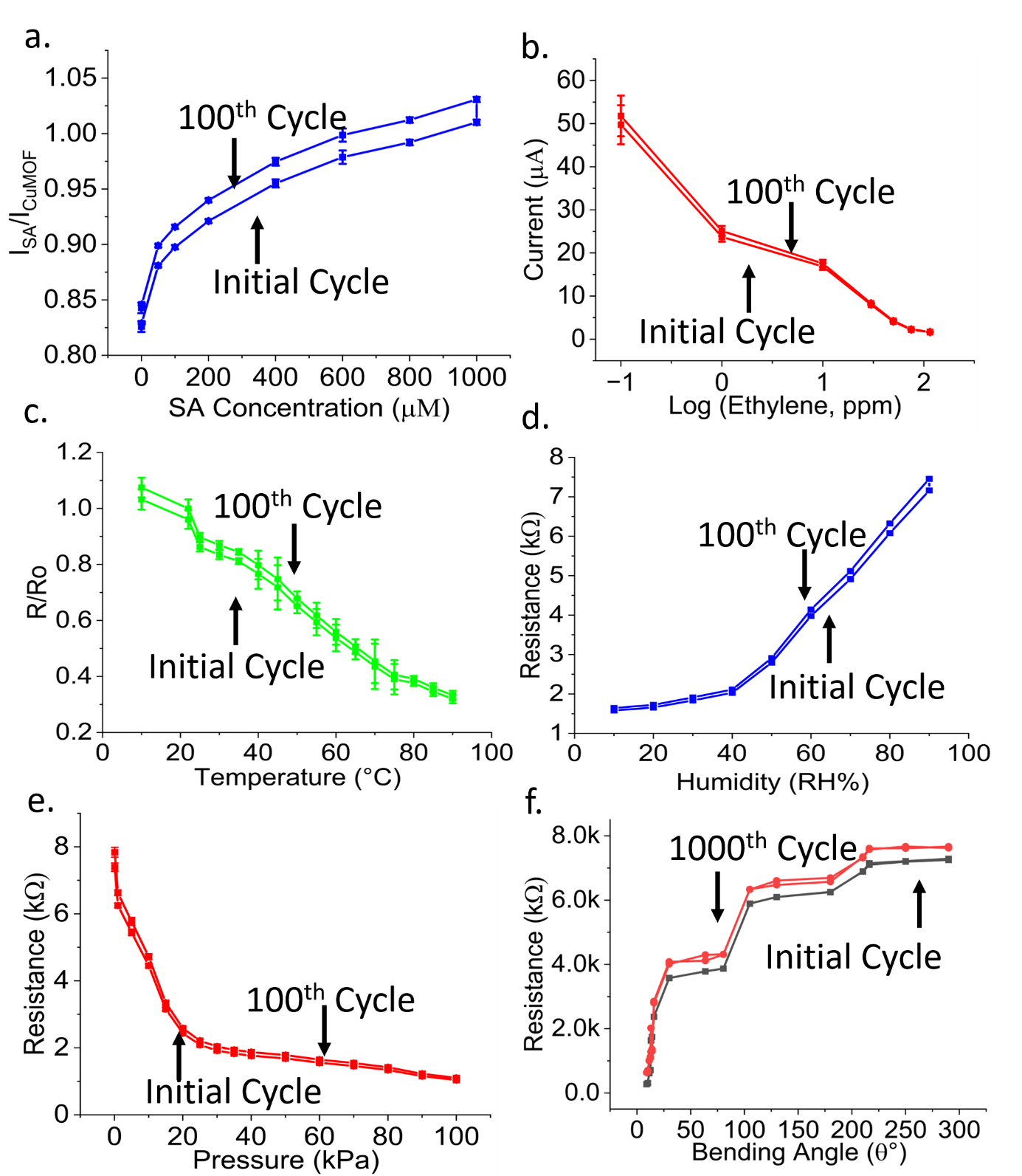


**Figure S11.** Hysteresis of (a) SA, (b) ethylene, (c) temperature, (d) humidity, (e) pressure, and (f) strain sensors. All measurements were repeated 3 times, with the error bars representing mean and standard error.


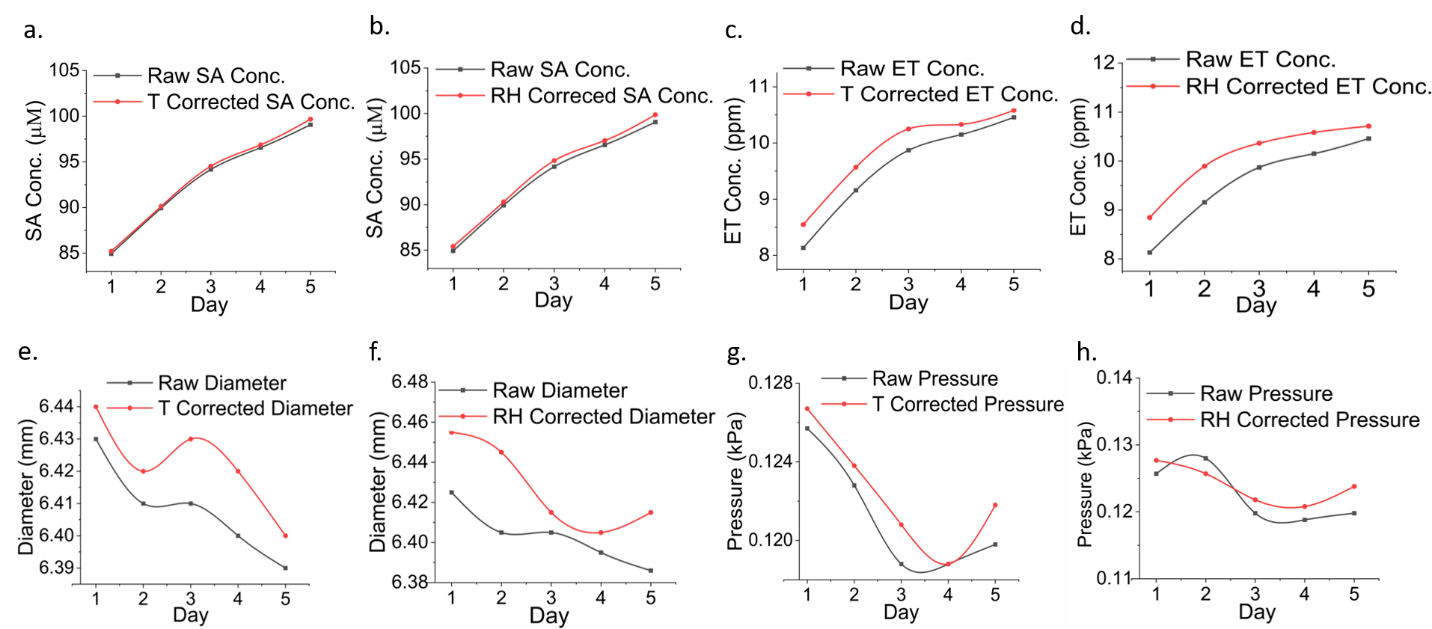


**Figure S12:** Real-time variations in SA concentrations before and after correcting for (a) temperature (T) and (b) relative humidity (RH) variations. Real-time variations in ethylene (ET) concentrations before and after correcting for (c) temperature (T) and (d) relative humidity (RH) variations. Real-time variations in stem diameter before and after correcting for (e) temperature (T) and (f) relative humidity (RH) variations. Real-time variations in the pressure applied on the strain sensor before and after correcting for (g) temperature (T) and (h) relative humidity (RH) variations. All measurements were recorded in water-stressed bell pepper plants.

**S5. Selectivity Analysis**

To successfully deploy the sensor in live plants, it is essential to evaluate the response of the sensors to interfering species. Hence, the SA sensor was tested against other chemical compounds typically found in plant sap (Figure S12a). The sensor was exposed to the following interfering species and their mixtures: (i) 50 µM of glucose, (ii) 50 µM of sucrose, (iii) 50 µM of soluble starch, (iv) 50 µM of L-tryptophan, (v) 50 µM of L-cysteine, (vi) 50 µM of abscisic acid (ABA), (vii) 50 µM of gibberellic acid (GA), (viii) 50 µM of Jasmonic acid (JA), (ix) 50 µM of oleic acid (OA), (x) 50 µM of indole-3-acetic acid (IAA), (xi) 50 µM of citric acid (CA), (xii) 50 µM of salicylic acid (SA), (xiii) a mixture of 50 µM glucose, soluble starch, L-tryptophan, L-cysteine, ABA, GA, JA, OA, IAA, CA each, (xiv) a mixture of 50 µM glucose, soluble starch, L-tryptophan, L-cysteine, ABA, GA, JA, OA, IAA, CA each, and 100 µM of SA, (xv) 900 µM of SA, and (xvi) a mixture of 50 µM glucose, soluble starch, L-tryptophan, L-cysteine, ABA, GA, JA, OA, IAA, CA each, and 900 µM of SA. The relative signals ($\frac{Ra-Rb}{Rb}$, where Ra = ratio of hormone redox current and CuMOF current and Rb =ratio of base current and CuMOF current) for the interfering compounds were significantly lower compared to the solutions containing SA. Moreover, the calibration curve of the SA sensor was plotted in presence of 50 µM of glucose, soluble starch, L- tryptophan, L-cysteine, ABA, GA, JA, OA, IAA, and CA each. Minute variation was observed between the calibration curves in the absence and presence of interferents (Figure S12b), particularly at lower SA concentrations, thereby confirming the very good selectivity of the sensor. With the addition of interfering molecules, the overall current response is decreased owing to the formation of a partition barrier that allows the passage of all molecules including the target analyte, into the recognition layer ^[S18]^. This phenomenon was also observed in another research work that reports SA sensing in the presence of interfering compounds ^[S19]^.

The ethylene sensor was also tested in response to various gaseous interferents typically present in agricultural lands or emitted from plants. For instance, the ethylene sensor was tested against the following gases: (i) 50 ppm of nitrogen (N_2_), (ii) 50 ppm of methane (CH_4_), (iii) 50 ppm of nitrous oxide (N_2_O), (iv) 50 ppm of ammonia (NH_3_), (v) 50 ppm of CO_2_, (vi) a mixture of 50 ppm of N_2,_ CH_4,_ N_2_O, NH_3_, CO_2_ each, (vii) 10 ppm of ethylene, (viii) 115 ppm of ethylene, (ix) a mixture of 50 ppm of N_2,_ CH_4,_ N_2_O, NH_3_, CO_2_ each, and 10 ppm of ethylene, and (x) a mixture of 50 ppm of N_2,_ CH_4,_ N_2_O, NH_3_, CO_2_ each, and 115 ppm of ethylene. The peak current variations from the baseline for gases other than ethylene were significantly less compared to when ethylene was present in the mixture (Figure S12c). Particularly, the interference current caused by 50 ppm of CO_2_ was higher compared to other interfering species. However, in comparison to ethylene, CO_2_ exhibited significantly lower interference current, indicating that the developed sensor demonstrated a much higher selectivity towards ethylene than CO_2_. Moreover, a negligible difference was observed between the ethylene sensor calibration plots in the presence and absence (Figure S12d) of the interfering gases (including CO_2_).


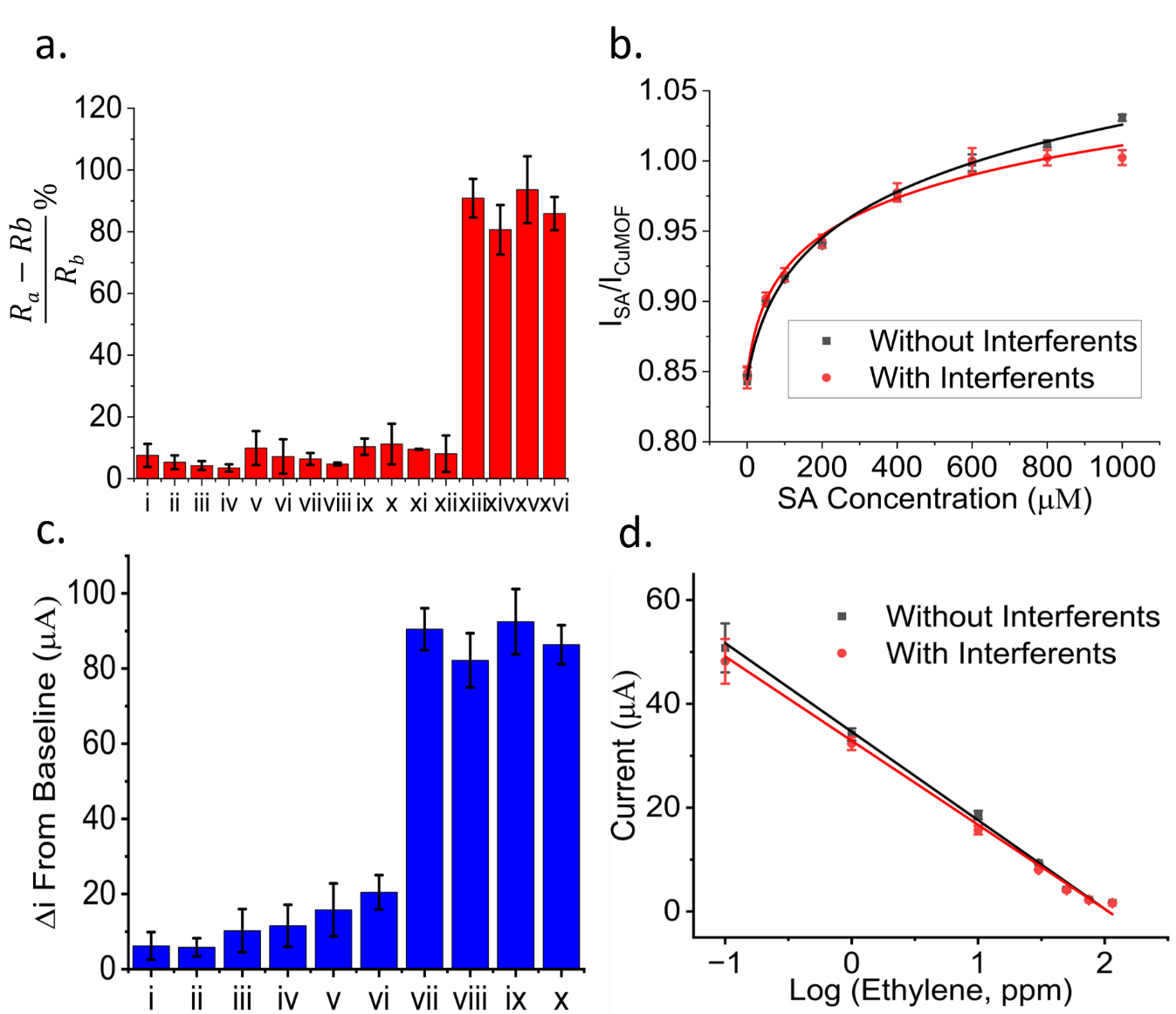


**Figure S13.** (a) Relative signal in response to different solutions introduced to the SA sensor, where i-xvi denote: (i) 50 µM of glucose, (ii) 50 µM of sucrose, (iii) 50 µM of soluble starch, (iv) 50 µM of L-tryptophan, (v) 50 µM of L-cysteine, (vi) 50 µM of abscisic acid (ABA), (vii) 50 µM of gibberellic acid (GA), (viii) 50 µM of Jasmonic acid (JA), (ix) 50 µM of oleic acid (OA), (x) 50 µM of indole-3-acetic acid (IAA), (xi) 50 µM of citric acid (CA), (xii) 50 µM of salicylic acid (SA), (xiii) a mixture of 50 µM glucose, soluble starch, L-tryptophan, L-cysteine, ABA, GA, JA, OA, IAA, CA each, (xiv) a mixture of 50 µM glucose, soluble starch, L-tryptophan, L-cysteine, ABA, GA, JA, OA, IAA, CA each, and 100 µM of SA, (xv) 900 µM of SA, and (xvi) a mixture of 50 µM glucose, soluble starch, L-tryptophan, L-cysteine, ABA, GA, JA, OA, IAA, CA each, and 900 µM of SA. (b) Calibration curve of the SA sensor in presence of 50 µM of glucose, soluble starch, L- tryptophan, L-cysteine, ABA, GA, JA, OA, IAA, CA each. (c) Current difference (in μA) from the baseline for the ethylene sensor where i-ix represent: (i) 50 ppm of nitrogen (N_2_), (ii) 50 ppm of methane (CH_4_), (iii) 50 ppm of nitrous oxide (N_2_O), (iv) 50 ppm of ammonia (NH_3_), (v) 50 ppm of CO_2_, (vi) a mixture of 50 ppm of N_2,_ CH_4,_ N_2_O, NH_3_, CO_2_ each, (vii) 10 ppm of ethylene, (viii) 115 ppm ethylene, (ix) a mixture of 50 ppm of N_2,_ CH_4,_ N_2_O, NH_3_, CO_2_ each, and 10 ppm of ethylene, and (x) a mixture of 50 ppm of N_2,_ CH_4,_ N_2_O, NH_3_, CO_2_ each, and 115 ppm of ethylene. (d) Calibration curve of the ethylene sensor in presence of 50 ppm of N_2,_ CH_4,_ N_2_O, and NH_3_ each. All measurements were repeated 3 times, with the error bars representing mean and standard error.

**Table S3.** Real-time monitoring of stem diameter over 40 days

| Day | Stem diameter in mm for plant #2 (unstressed and kept in sunlight) | Stem diameter in mm for plant #4 (unstressed and kept in shade) | Stem diameter in mm for plant #1  (stressed and kept in sunlight) | Stem diameter in mm for plant #3 (stressed and kept in shade) |
| --- | --- | --- | --- | --- |
| 1  2  3  4  5  6  7  8  9  10  11  12  13  14  15  16  17  18  19  20  21  22  23  24  25  26  27  28  29  30  31  32  33  34  35  36  37  38  39  40 | 6.49  6.49  6.5  6.52  6.52  6.53  6.54  6.57  6.6  6.61  6.64  6.65  6.68  6.68  6.68  6.68  6.69  6.69  6.7  6.7  6.7  6.71  6.72  6.73  6.74  6.75  6.75  6.75  6.78  6.79  6.8  6.81  6.83  6.84  6.85  6.87  6.87  6.87  6.87  6.87 | 6.17  6.17  6.18  6.19  6.19  6.2  6.21  6.24  6.27  6.28  6.31  6.32  6.35  6.35  6.35  6.35  6.36  6.36  6.37  6.37  6.37  6.37  6.38  6.39  6.4  6.41  6.41  6.41  6.44  6.45  6.46  6.47  6.49  6.5  6.51  6.53  6.53  6.53  6.53  6.53 | 6.94  6.94  6.94  6.92  6.91  6.9  6.88  6.87  6.86  6.85  6.82  6.82  6.82  6.81  6.8  6.79  6.78  6.77  6.77  6.77  6.76  6.76  6.75  6.75  6.75  6.75  6.72  6.71  6.68  6.67  6.64  6.61  6.6  6.59  6.59  6.57  6.55  6.55  6.55  6.54 | 6.59  6.59  6.59  6.57  6.56  6.56  6.54  6.53  6.52  6.51  6.48  6.48  6.48  6.47  6.46  6.45  6.44  6.43  6.43  6.43  6.42  6.42  6.41  6.41  6.41  6.41  6.38  6.37  6.35  6.34  6.31  6.28  6.27  6.26  6.26  6.24  6.22  6.22  6.22  6.21 |
|  |  |  |  |  |


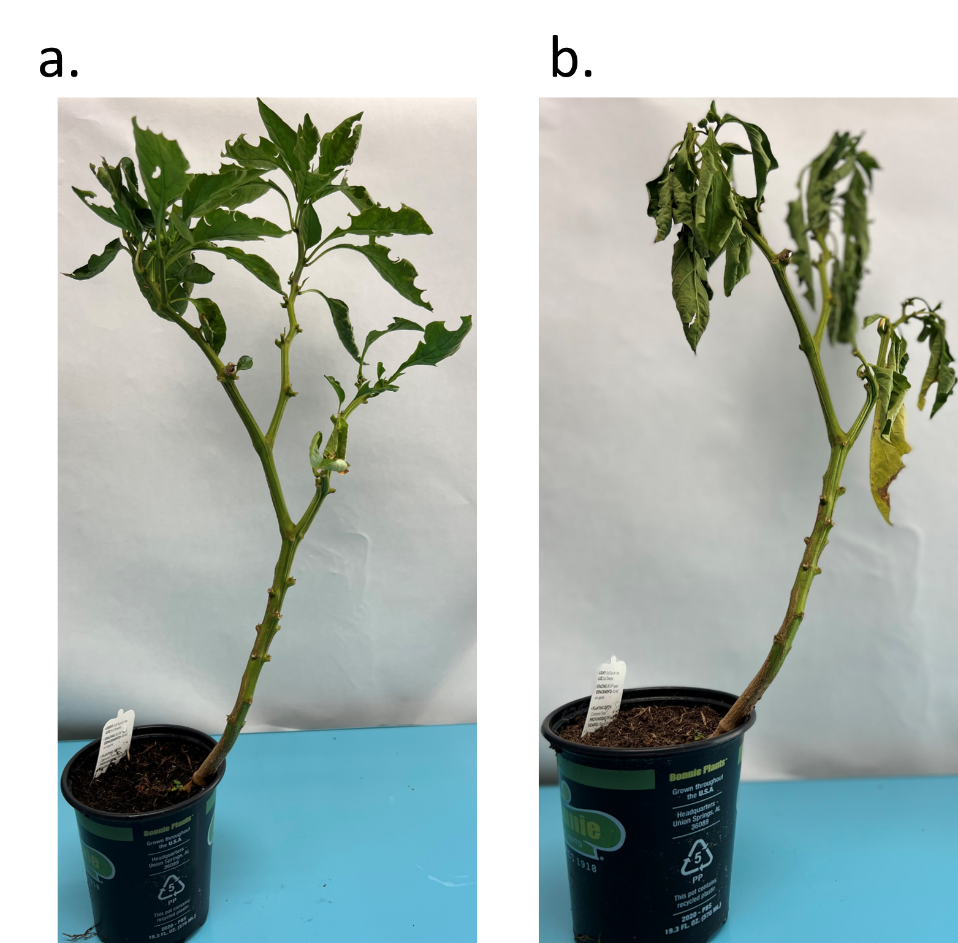


**Figure S14.** Optical images of (a) unstressed and (b) water-stressed bell pepper plants taken after 40 days of measurements.


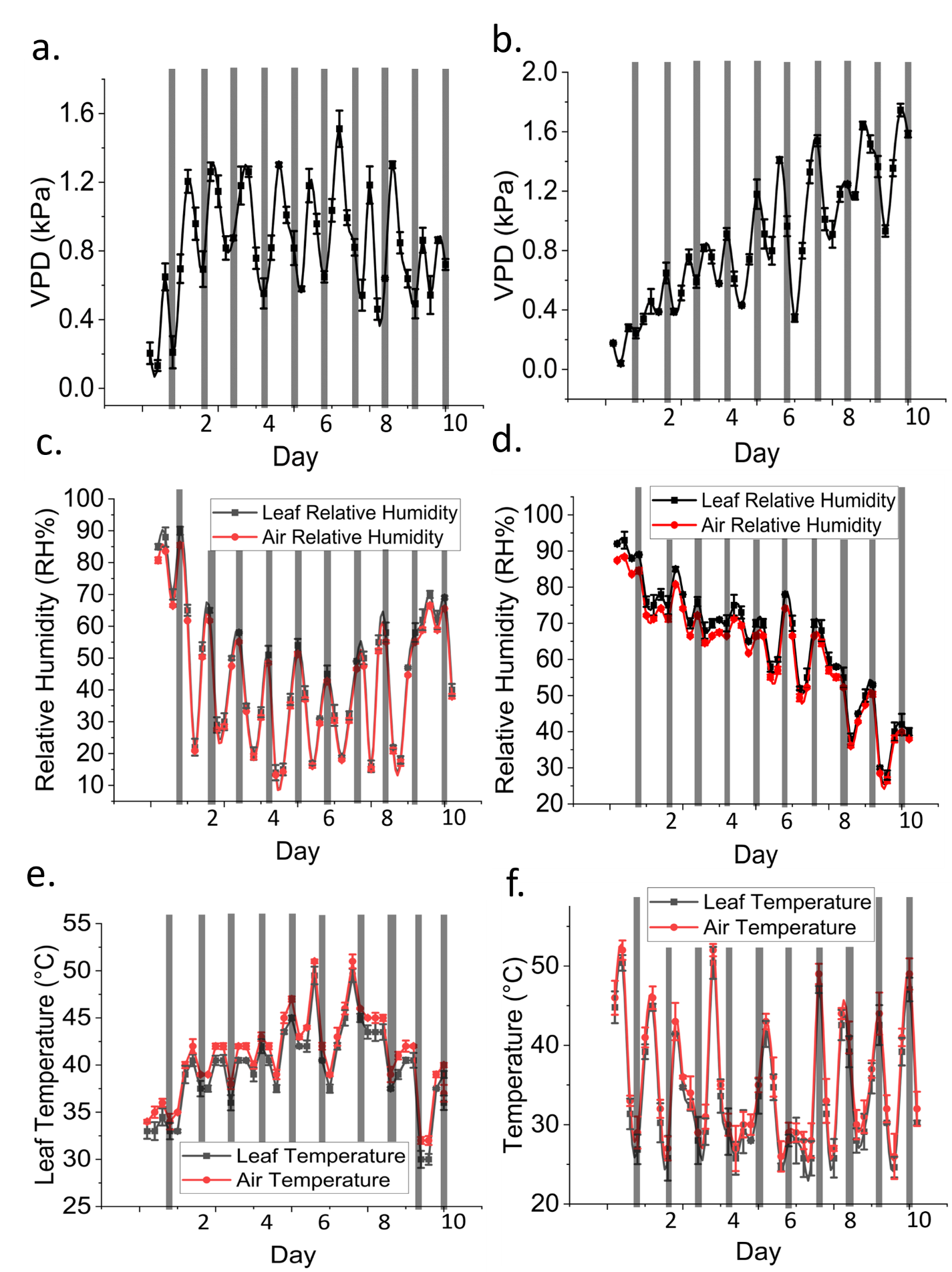


**Figure S15.** 10 days of VPD measurements from (a) control and (b) water-stressed plants. 10 days of leaf relative humidity measurements from (c) control and (d) water-stressed plants. 10 days of leaf temperature measurements from (e) control and (f) water-stressed plants.


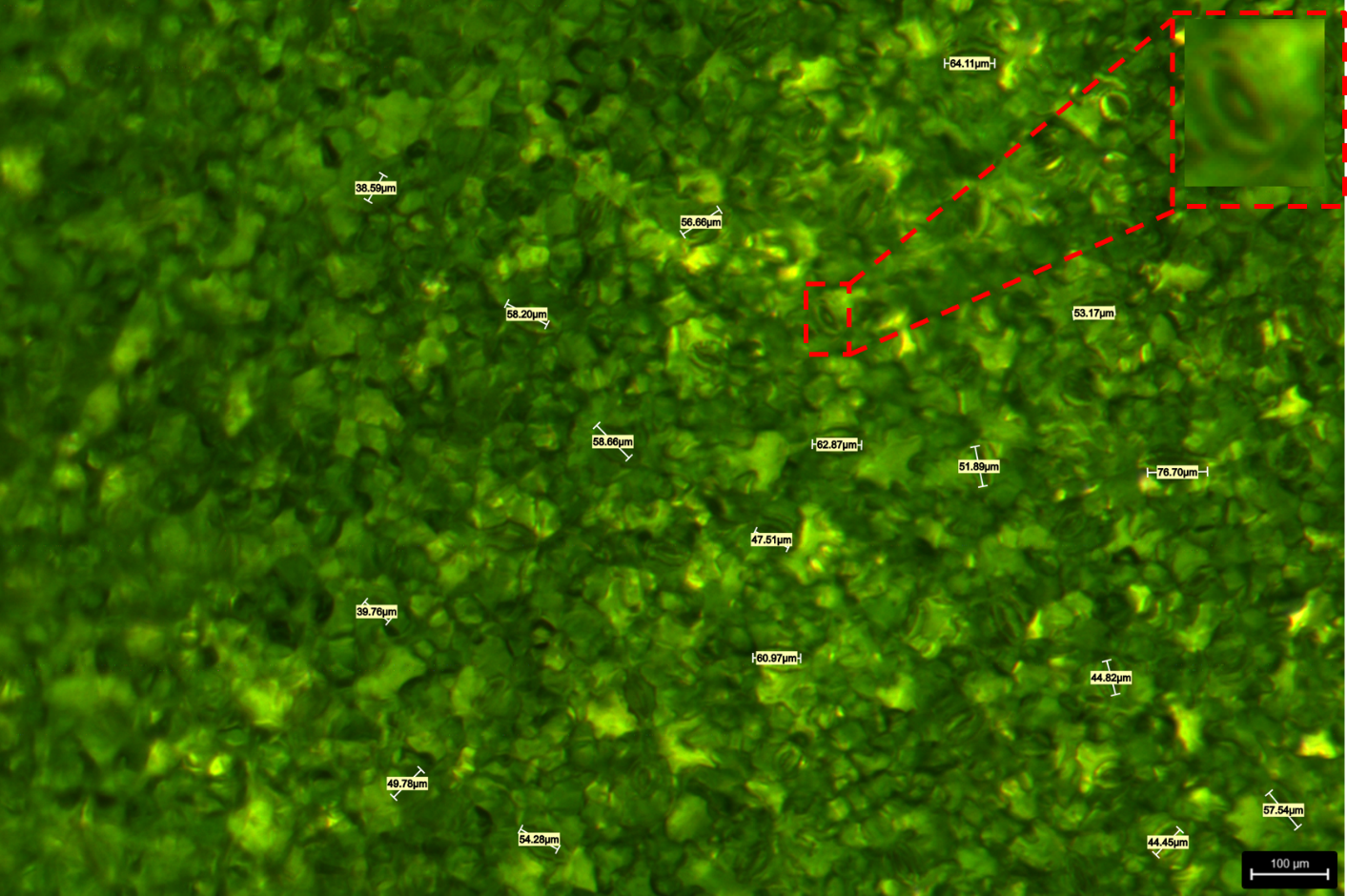


**Figure S16.**  Microscopic image of the leaf of an unstressed plant, depicting open stomata. The inset at the top-right corner shows the zoomed-in image of one opened stomata.


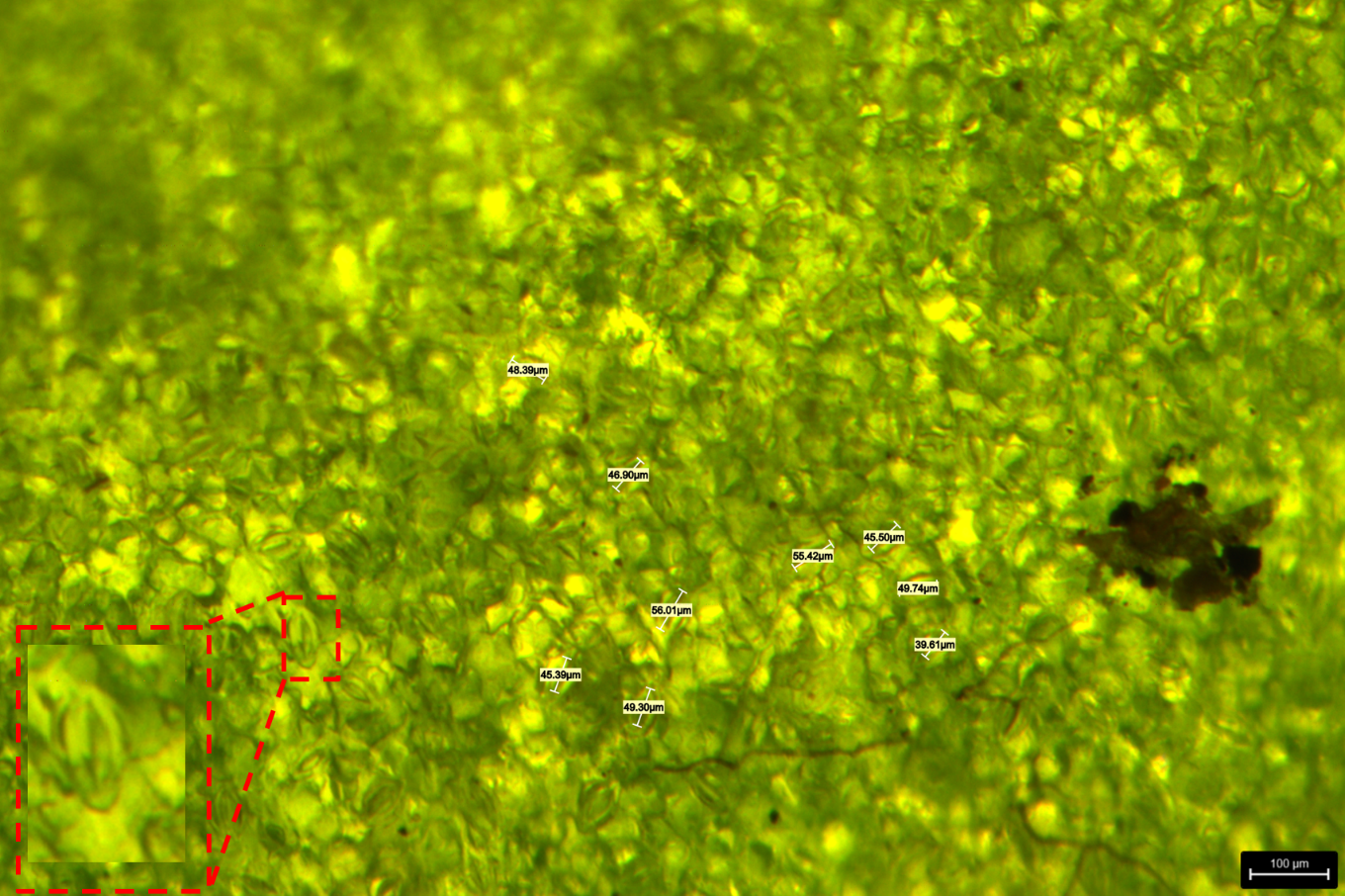


**Figure S17.**  Microscopic image of the leaf of a water-stressed plant, depicting closed stomata. The inset at the bottom-left corner shows the zoomed-in image of one closed stomata.


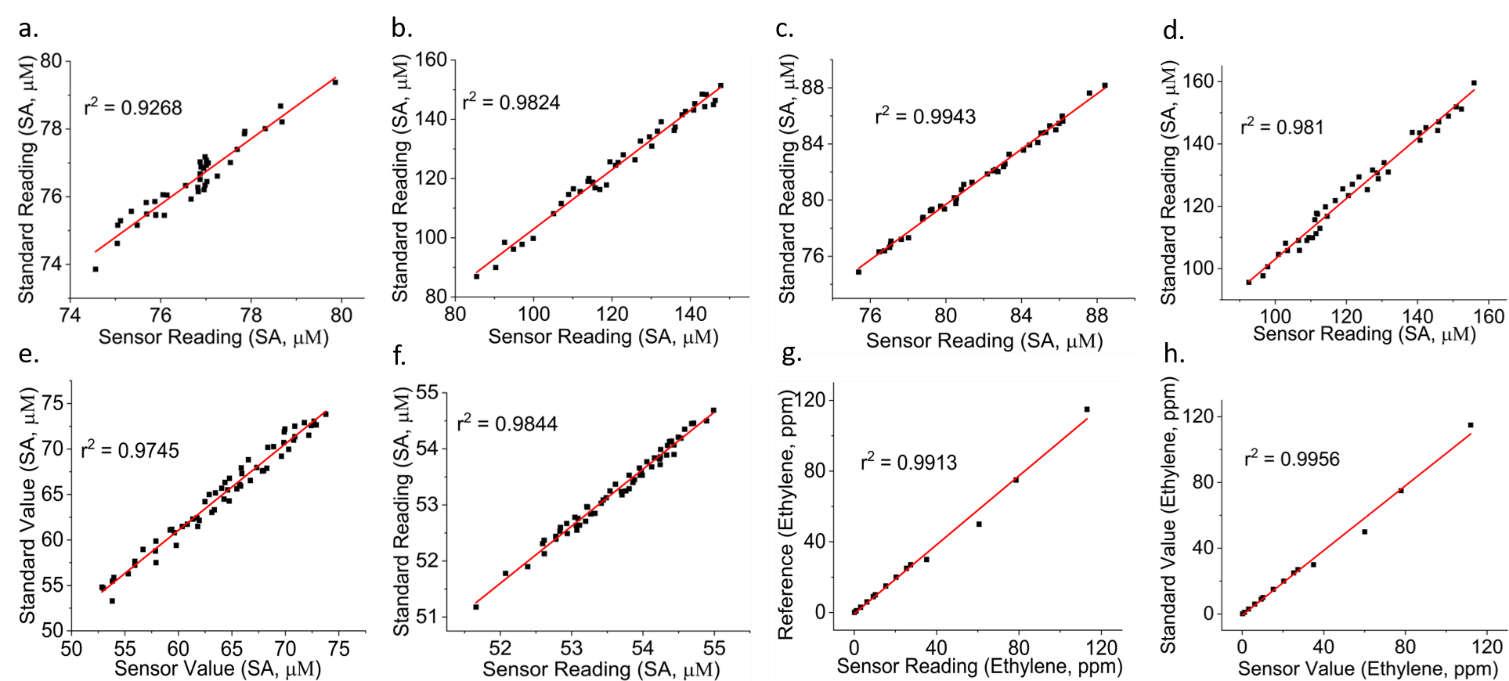


**Figure S18.** Comparing the SA levels measured with our sensor against the values from high-performance liquid chromatography in (a) control and (b) water-stressed bell pepper plants kept in sunlight, and (c) control and (d) water-stressed bell pepper plants kept in the shade over 40 days. Comparing the SA levels measured with our sensor against the values from high-performance liquid chromatography in (e) control and (f) water-stressed cabbage plants over 2 months. Comparing the ethylene levels estimated with our sensor against known concentrations of (g) pure ethylene and (h) ethylene mixed with interfering gases (i.e., N_2,_ CH_4,_ N_2_O, and NH_3_).

**Table S4**: VPD measurements with commercial (temperature sensor LM35, Texas Instruments, TX and humidity sensor DHT11, Adafruit, NY) and developed sensors when the plants were kept in sunlight

| VPD of Plants kept in the Sunlight | | | | |
| --- | --- | --- | --- | --- |
| Hour | VPD in kPa with our sensors (stressed plant) | VPD in kPa with commercial sensors (stressed plant) | VPD in kPa with our sensors (control plant) | VPD in kPa with commercial sensors (control plant) |
| 12 | 0.04081 | 0.040402 | 1.16426 | 1.152617 |
| 24 | 0.28002 | 0.27722 | 1.34812 | 1.334639 |
| 36 | 0.24321 | 0.240778 | 0.68033 | 0.673527 |
| 48 | 0.33989 | 0.336491 | 0.85107 | 0.842559 |
| 60 | 0.45781 | 0.453232 | 0.95876 | 0.949172 |
| 72 | 0.38812 | 0.384239 | 0.58601 | 0.58015 |
| 84 | 0.64913 | 0.642639 | 0.7987 | 0.790713 |
| 96 | 0.38948 | 0.385585 | 0.63471 | 0.628363 |
| 108 | 0.51421 | 0.509068 | 0.63864 | 0.632254 |
| 120 | 0.75737 | 0.749796 | 0.81893 | 0.810741 |
| 132 | 0.59086 | 0.584951 | 0.55071 | 0.545203 |
| 144 | 0.81817 | 0.809988 | 1.01109 | 1.000979 |
| 156 | 0.75737 | 0.749796 | 0.74774 | 0.740263 |
| 168 | 0.57939 | 0.573596 | 1.0105 | 1.000395 |
| 180 | 0.91186 | 0.902741 | 0.64663 | 0.640164 |
| 192 | 0.43272 | 0.428393 | 0.70106 | 0.694049 |
| 204 | 0.74088 | 0.733471 | 1.06413 | 1.053489 |
| 216 | 1.1796 | 1.167804 | 0.90935 | 0.900257 |
| 228 | 0.91186 | 0.902741 | 0.82068 | 0.812473 |
| 240 | 0.7987 | 0.790713 | 1.20236 | 1.190336 |
| 252 | 1.40924 | 1.395148 | 0.93562 | 0.926264 |
| 264 | 0.96434 | 0.954697 | 1.21331 | 1.201177 |
| 276 | 0.34617 | 0.342708 | 1.00643 | 0.996366 |
| 288 | 0.7987 | 0.790713 | 0.77384 | 0.766102 |
| 300 | 1.3292 | 1.315908 | 0.76809 | 0.760409 |
| 312 | 1.53938 | 1.523986 | 1.18792 | 1.176041 |
| 324 | 1.01109 | 1.000979 | 0.92666 | 0.917393 |
| 336 | 0.90939 | 0.900296 | 0.81673 | 0.808563 |
| 348 | 1.17902 | 1.16723 | 0.81716 | 0.808988 |
| 360 | 1.24642 | 1.233956 | 1.27052 | 1.257815 |
| 372 | 1.16843 | 1.156746 | 1.06466 | 1.054013 |
| 384 | 1.64114 | 1.624729 | 0.74672 | 0.739253 |
| 396 | 1.51726 | 1.502087 | 1.03386 | 1.023521 |
| 408 | 1.36528 | 1.351627 | 1.25912 | 1.246529 |
| 420 | 0.93185 | 0.922532 | 0.84885 | 0.840362 |
| 432 | 1.35444 | 1.340896 | 0.70106 | 0.694049 |
| 444 | 1.74544 | 1.727986 | 0.95811 | 0.948529 |
| 456 | 1.58344 | 1.567606 | 1.22043 | 1.208226 |
| 468 | 0.99493 | 0.984981 | 0.9445 | 0.935055 |
| 480 | 0.83091 | 0.822601 | 0.82124 | 0.813028 |
| 492 | 1.85308 | 1.834549 | 0.68908 | 0.682189 |
| 504 | 1.0909 | 1.079991 | 1.20574 | 1.193683 |
| 516 | 1.05008 | 1.039579 | 1.06466 | 1.054013 |
| 528 | 1.0909 | 1.079991 | 0.95207 | 0.942549 |
| 540 | 2.11424 | 2.093098 | 0.64913 | 0.642639 |
| 552 | 2.13706 | 2.115689 | 0.90935 | 0.900257 |
| 564 | 0.9334 | 0.924066 | 0.71793 | 0.710751 |
| 576 | 1.16368 | 1.152043 | 0.77895 | 0.771161 |
| 588 | 2.17331 | 2.151577 | 0.71793 | 0.710751 |
| 600 | 1.69142 | 1.674506 | 1.24262 | 1.230194 |
| 612 | 1.24632 | 1.233857 | 1.06286 | 1.052231 |
| 624 | 0.73503 | 0.72768 | 0.77895 | 0.771161 |
| 636 | 1.43894 | 1.424551 | 0.18082 | 0.179012 |
| 648 | 2.23919 | 2.216798 | 0.09314 | 0.092209 |
| 660 | 0.95811 | 0.948529 | 0.59816 | 0.592178 |
| 672 | 0.68908 | 0.682189 | 0.18668 | 0.184813 |
| 684 | 1.64381 | 1.627372 | 0.61842 | 0.612236 |
| 696 | 1.73809 | 1.720709 | 1.18062 | 1.168814 |
| 708 | 0.80781 | 0.799732 | 1.0105 | 1.000395 |
| 720 | 0.54052 | 0.535115 | 0.81673 | 0.808563 |
| 732 | 1.03093 | 1.020621 | 1.12133 | 1.110117 |
| 744 | 0.66023 | 0.653628 | 1.17902 | 1.16723 |
| 756 | 0.77895 | 0.771161 | 0.80428 | 0.796237 |
| 768 | 0.74678 | 0.739312 | 1.14973 | 1.138233 |
| 780 | 1.65532 | 1.638767 | 1.12042 | 1.109216 |
| 792 | 2.04416 | 2.023718 | 0.93474 | 0.925393 |
| 804 | 0.77895 | 0.771161 | 0.61293 | 0.606801 |
| 816 | 0.22749 | 0.225215 | 0.51337 | 0.508236 |
| 828 | 0.09314 | 0.092209 | 1.30793 | 1.294851 |
| 840 | 0.68029 | 0.673487 | 0.98436 | 0.974516 |
| 852 | 0.18668 | 0.184813 | 1.21222 | 1.200098 |
| 864 | 0.61842 | 0.612236 | 0.78782 | 0.779942 |
| 876 | 1.57314 | 1.557409 | 0.43587 | 0.431511 |
| 888 | 1.31383 | 1.300692 | 1.24202 | 1.2296 |
| 900 | 0.74086 | 0.733451 | 1.02279 | 1.012562 |
| 912 | 1.86773 | 1.849053 | 0.78782 | 0.779942 |
| 924 | 1.75197 | 1.73445 | 0.77563 | 0.767874 |
| 936 | 1.47095 | 1.456241 | 0.8887 | 0.879813 |
| 948 | 1.09498 | 1.08403 | 0.7881 | 0.780219 |
| 960 | 1.69663 | 1.679664 | 0.91092 | 0.901811 |
| 972 | 2.24093 | 2.218521 | 1.21331 | 1.201177 |
| 984 | 1.85994 | 1.841341 | 1.37517 | 1.361418 |
| 996 | 1.05008 | 1.039579 | 0.71793 | 0.710751 |
| 1008 | 2.24093 | 2.218521 | 0.64913 | 0.642639 |
| 1020 | 2.35817 | 2.334588 | 1.01736 | 1.007186 |
| 1032 | 2.30645 | 2.283386 | 1.36922 | 1.355528 |
| 1044 | 1.10787 | 1.096791 | 0.7987 | 0.790713 |
| 1056 | 0.54642 | 0.540956 | 0.68437 | 0.677526 |
| 1068 | 1.77409 | 1.756349 | 0.68029 | 0.673487 |
| 1080 | 1.82309 | 1.804859 | 0.95876 | 0.949172 |
| 1092 | 1.23097 | 1.21866 | 0.96041 | 0.950806 |
| 1104 | 1.38149 | 1.367675 | 0.97265 | 0.962924 |
| 1116 | 2.40694 | 2.382871 | 0.77421 | 0.766468 |
| 1128 | 2.24042 | 2.218016 | 0.49042 | 0.485516 |
| 1140 | 0.98477 | 0.974922 | 1.03627 | 1.025907 |
| 1152 | 1.21331 | 1.201177 | 0.61548 | 0.609325 |
| 1164 | 2.51465 | 2.489504 | 0.34927 | 0.345777 |
| 1176 | 1.30938 | 1.296286 | 0.23335 | 0.231017 |
| 1188 | 1.0386 | 1.028214 | 0.11668 | 0.115513 |
| 1200 | 1.52291 | 1.507681 | 0.07001 | 0.06931 |
| 1212 | 2.59986 | 2.573861 | 0.46129 | 0.456677 |
| 1224 | 1.43894 | 1.424551 | 1.06466 | 1.054013 |
| 1236 | 1.23186 | 1.219541 | 0.98436 | 0.974516 |
| 1248 | 0.68029 | 0.673487 | 0.64913 | 0.642639 |
| 1260 | 0.95876 | 0.949172 | 0.2549 | 0.252351 |
| 1272 | 1.9581 | 1.938519 | 0.54341 | 0.537976 |
| 1284 | 0.91186 | 0.902741 | 0.54818 | 0.542698 |
| 1296 | 1.16368 | 1.152043 | 0.31158 | 0.308464 |
| 1308 | 1.29824 | 1.285258 | 0.95876 | 0.949172 |
| 1320 | 2.68707 | 2.660199 | 0.69658 | 0.689614 |
| 1332 | 0.98477 | 0.974922 | 0.56649 | 0.560825 |
| 1344 | 0.39116 | 0.387248 | 0.5817 | 0.575883 |
| 1356 | 0.28002 | 0.27722 | 0.74323 | 0.735798 |
| 1368 | 0.11668 | 0.115513 | 0.95596 | 0.9464 |
| 1380 | 0.11668 | 0.115513 | 0.60085 | 0.594842 |
| 1392 | 0.51604 | 0.51088 | 0.56541 | 0.559756 |
| 1404 | 1.41937 | 1.405176 | 0.58773 | 0.581853 |
| 1416 | 1.8479 | 1.829421 | 0.86273 | 0.854103 |
| 1428 | 0.77895 | 0.771161 | 0.93562 | 0.926264 |
| 1440 | 0.2549 | 0.252351 | 0.71011 | 0.703009 |
| 1452 | 0.68029 | 0.673487 | 0.83091 | 0.822601 |
| 1464 | 0.80428 | 0.796237 | 1.31296 | 1.29983 |
| 1476 | 0.5193 | 0.514107 | 1.37747 | 1.363695 |
| 1488 | 0.95876 | 0.949172 | 0.58762 | 0.581744 |
| 1500 | 0.81817 | 0.809988 | 0.37496 | 0.37121 |
| 1512 | 0.90403 | 0.89499 | 0.77384 | 0.766102 |
| 1524 | 0.75059 | 0.743084 | 0.81806 | 0.809879 |
| 1536 | 0.83705 | 0.82868 | 0.70751 | 0.700435 |
| 1548 | 1.68298 | 1.66615 | 0.18029 | 0.178487 |
| 1560 | 1.39424 | 1.380298 | 1.25328 | 1.240747 |
| 1572 | 1.25646 | 1.243895 | 1.27301 | 1.26028 |
| 1584 | 1.18253 | 1.170705 | 0.63005 | 0.62375 |
| 1596 | 2.3993 | 2.375307 | 0.73324 | 0.725908 |
| 1608 | 1.04032 | 1.029917 | 0.99372 | 0.983783 |
| 1620 | 0.79888 | 0.790891 | 1.12946 | 1.118165 |
| 1632 | 1.04032 | 1.029917 | 0.77384 | 0.766102 |
| 1644 | 2.41257 | 2.388444 | 0.88194 | 0.873121 |
| 1656 | 2.28933 | 2.266437 | 1.27848 | 1.265695 |
| 1668 | 0.83946 | 0.831065 | 1.0909 | 1.079991 |
| 1680 | 0.44995 | 0.445451 | 0.9334 | 0.924066 |
| 1692 | 0.97282 | 0.963092 | 0.07147 | 0.070755 |
| 1704 | 0.81806 | 0.809879 | 1.1796 | 1.167804 |
| 1716 | 0.77384 | 0.766102 | 1.34812 | 1.334639 |
| 1728 | 0.23658 | 0.234214 | 0.66329 | 0.656657 |
| 1740 | 1.52291 | 1.507681 | 1.41937 | 1.405176 |
| 1752 | 1.85052 | 1.832015 | 1.30743 | 1.294356 |
| 1764 | 0.70005 | 0.69305 | 0.86273 | 0.854103 |
| 1776 | 0.73324 | 0.725908 | 0.64438 | 0.637936 |
| 1788 | 1.59673 | 1.580763 | 1.00643 | 0.996366 |
| 1800 | 1.70567 | 1.688613 | 1.22549 | 1.213235 |
| 1812 | 0.99493 | 0.984981 | 0.74814 | 0.740659 |
| 1824 | 0.93823 | 0.928848 | 0.70005 | 0.69305 |

**Table S5**: VPD measurements with commercial (temperature sensor LM35, Texas Instruments, TX and humidity sensor DHT11, Adafruit, NY) and developed sensors when the plants were kept in shade

| VPD of Plants kept in the Shade | | | | |
| --- | --- | --- | --- | --- |
|  | VPD in kPa with our sensors (stressed plant) | VPD in kPa with commercial sensors (stressed plant) | VPD in kPa with our sensors (control plant) | VPD in kPa with commercial sensors (control plant) |
| 12 | 0.70914 | 0.702049 | 0.77458 | 0.766834 |
| 24 | 1.11057 | 1.099464 | 0.64594 | 0.639481 |
| 36 | 0.86288 | 0.854251 | 0.70824 | 0.701158 |
| 48 | 0.61548 | 0.609325 | 0.46883 | 0.464142 |
| 60 | 0.7676 | 0.759924 | 0.80522 | 0.797168 |
| 72 | 1.25578 | 1.243222 | 0.45781 | 0.453232 |
| 84 | 1.06286 | 1.052231 | 0.57367 | 0.567933 |
| 96 | 0.91186 | 0.902741 | 0.49543 | 0.490476 |
| 108 | 1.01736 | 1.007186 | 0.76678 | 0.759112 |
| 120 | 1.38257 | 1.368744 | 0.62267 | 0.616443 |
| 132 | 0.86273 | 0.854103 | 0.51182 | 0.506702 |
| 144 | 0.75988 | 0.752281 | 0.70718 | 0.700108 |
| 156 | 1.46573 | 1.451073 | 0.962 | 0.95238 |
| 168 | 0.66619 | 0.659528 | 0.7987 | 0.790713 |
| 180 | 0.43272 | 0.428393 | 0.76669 | 0.759023 |
| 192 | 0.4881 | 0.483219 | 0.77895 | 0.771161 |
| 204 | 1.53262 | 1.517294 | 0.87367 | 0.864933 |
| 216 | 0.99576 | 0.985802 | 0.58689 | 0.581021 |
| 228 | 0.67347 | 0.666735 | 0.51421 | 0.509068 |
| 240 | 0.74146 | 0.734045 | 0.88308 | 0.874249 |
| 252 | 1.30793 | 1.294851 | 1.0105 | 1.000395 |
| 264 | 0.80428 | 0.796237 | 0.77539 | 0.767636 |
| 276 | 0.96434 | 0.954697 | 0.6054 | 0.599346 |
| 288 | 0.57123 | 0.565518 | 0.55274 | 0.547213 |
| 300 | 1.29854 | 1.285555 | 1.00643 | 0.996366 |
| 312 | 1.45744 | 1.442866 | 0.87367 | 0.864933 |
| 324 | 0.75737 | 0.749796 | 0.68029 | 0.673487 |
| 336 | 0.53532 | 0.529967 | 0.58338 | 0.577546 |
| 348 | 1.68456 | 1.667714 | 1.03169 | 1.021373 |
| 360 | 1.51893 | 1.503741 | 1.28357 | 1.270734 |
| 372 | 1.21331 | 1.201177 | 1.01109 | 1.000979 |
| 384 | 0.66329 | 0.656657 | 0.86168 | 0.853063 |
| 396 | 1.58344 | 1.567606 | 1.14727 | 1.135797 |
| 408 | 2.01173 | 1.991613 | 1.18064 | 1.168834 |
| 420 | 1.36465 | 1.351004 | 0.70267 | 0.695643 |
| 432 | 1.16675 | 1.155083 | 0.61548 | 0.609325 |
| 444 | 1.85518 | 1.836628 | 1.25685 | 1.244282 |
| 456 | 2.01861 | 1.998424 | 0.99576 | 0.985802 |
| 468 | 1.69142 | 1.674506 | 1.07791 | 1.067131 |
| 480 | 1.15711 | 1.145539 | 0.90878 | 0.899692 |
| 492 | 2.01328 | 1.993147 | 0.86218 | 0.853558 |
| 504 | 1.87786 | 1.859081 | 1.32068 | 1.307473 |
| 516 | 1.1244 | 1.113156 | 1.10013 | 1.089129 |
| 528 | 0.97944 | 0.969646 | 0.80781 | 0.799732 |
| 540 | 1.36903 | 1.35534 | 0.36395 | 0.360311 |
| 552 | 1.82005 | 1.80185 | 0.71793 | 0.710751 |
| 564 | 1.75197 | 1.73445 | 0.5193 | 0.514107 |
| 576 | 0.96071 | 0.951103 | 0.6401 | 0.633699 |
| 588 | 0.42942 | 0.425126 | 1.24202 | 1.2296 |
| 600 | 2.16742 | 2.145746 | 0.78737 | 0.779496 |
| 612 | 1.24202 | 1.2296 | 0.63618 | 0.629818 |
| 624 | 0.68908 | 0.682189 | 0.20415 | 0.202109 |
| 636 | 0.36395 | 0.360311 | 0.13279 | 0.131462 |
| 648 | 0.71793 | 0.710751 | 0.64913 | 0.642639 |
| 660 | 0.5193 | 0.514107 | 0.21002 | 0.20792 |
| 672 | 0.77468 | 0.766933 | 0.69631 | 0.689347 |
| 684 | 1.06466 | 1.054013 | 1.20513 | 1.193079 |
| 696 | 1.51985 | 1.504652 | 0.95876 | 0.949172 |
| 708 | 1.68113 | 1.664319 | 0.69419 | 0.687248 |
| 720 | 0.22749 | 0.225215 | 1.26136 | 1.248746 |
| 732 | 0.54341 | 0.537976 | 1.14727 | 1.135797 |
| 744 | 0.75299 | 0.74546 | 0.81716 | 0.808988 |
| 756 | 0.28002 | 0.27722 | 0.87599 | 0.86723 |
| 768 | 0.36016 | 0.356558 | 1.18062 | 1.168814 |
| 780 | 1.74086 | 1.723451 | 1.26136 | 1.248746 |
| 792 | 1.18285 | 1.171022 | 0.75737 | 0.749796 |
| 804 | 0.9334 | 0.924066 | 0.55274 | 0.547213 |
| 816 | 1.98133 | 1.961517 | 0.81864 | 0.810454 |
| 828 | 1.37517 | 1.361418 | 1.30192 | 1.288901 |
| 840 | 0.81716 | 0.808988 | 1.00977 | 0.999672 |
| 852 | 0.82124 | 0.813028 | 0.81673 | 0.808563 |
| 864 | 2.04416 | 2.023718 | 0.57942 | 0.573626 |
| 876 | 2.25825 | 2.235668 | 1.18064 | 1.168834 |
| 888 | 1.66924 | 1.652548 | 0.95825 | 0.948668 |
| 900 | 0.88439 | 0.875546 | 0.64913 | 0.642639 |
| 912 | 2.42741 | 2.403136 | 1.03627 | 1.025907 |
| 924 | 1.94082 | 1.921412 | 1.51144 | 1.496326 |
| 936 | 1.44253 | 1.428105 | 0.99372 | 0.983783 |
| 948 | 0.70005 | 0.69305 | 0.82124 | 0.813028 |
| 960 | 1.4662 | 1.451538 | 0.54261 | 0.537184 |
| 972 | 2.51699 | 2.49182 | 1.18371 | 1.171873 |
| 984 | 1.95144 | 1.931926 | 0.46129 | 0.456677 |
| 996 | 1.42808 | 1.413799 | 0.6401 | 0.633699 |
| 1008 | 2.27437 | 2.251626 | 1.30192 | 1.288901 |
| 1020 | 2.06527 | 2.044617 | 0.84759 | 0.839114 |
| 1032 | 2.1288 | 2.107512 | 0.63864 | 0.632254 |
| 1044 | 1.36873 | 1.355043 | 0.49274 | 0.487813 |
| 1056 | 0.95876 | 0.949172 | 0.86218 | 0.853558 |
| 1068 | 2.43329 | 2.408957 | 0.54341 | 0.537976 |
| 1080 | 0.81716 | 0.808988 | 0.86273 | 0.854103 |
| 1092 | 0.86168 | 0.853063 | 0.72126 | 0.714047 |
| 1104 | 2.51582 | 2.490662 | 1.18062 | 1.168814 |
| 1116 | 2.45937 | 2.434776 | 1.24262 | 1.230194 |
| 1128 | 0.86273 | 0.854103 | 0.90935 | 0.900257 |
| 1140 | 0.95811 | 0.948529 | 0.81673 | 0.808563 |
| 1152 | 0.86218 | 0.853558 | 0.4121 | 0.407979 |
| 1164 | 0.62554 | 0.619285 | 0.23335 | 0.231017 |
| 1176 | 0.95876 | 0.949172 | 0.11668 | 0.115513 |
| 1188 | 0.86552 | 0.856865 | 0.11668 | 0.115513 |
| 1200 | 2.16191 | 2.140291 | 0.54341 | 0.537976 |
| 1212 | 2.56326 | 2.537627 | 1.12133 | 1.110117 |
| 1224 | 1.66924 | 1.652548 | 1.1356 | 1.124244 |
| 1236 | 0.9334 | 0.924066 | 0.98549 | 0.975635 |
| 1248 | 0.5168 | 0.511632 | 0.11776 | 0.116582 |
| 1260 | 0.28002 | 0.27722 | 0.48705 | 0.48218 |
| 1272 | 0.11668 | 0.115513 | 0.46129 | 0.456677 |
| 1284 | 0.11668 | 0.115513 | 0.36929 | 0.365597 |
| 1296 | 0.54341 | 0.537976 | 1.32516 | 1.311908 |
| 1308 | 1.49453 | 1.479585 | 0.95876 | 0.949172 |
| 1320 | 1.3839 | 1.370061 | 0.61015 | 0.604049 |
| 1332 | 0.95811 | 0.948529 | 0.75341 | 0.745876 |
| 1344 | 0.167 | 0.16533 | 0.57751 | 0.571735 |
| 1356 | 0.48705 | 0.48218 | 0.84885 | 0.840362 |
| 1368 | 0.59816 | 0.592178 | 0.74086 | 0.733451 |
| 1380 | 0.44315 | 0.438719 | 0.83764 | 0.829264 |
| 1392 | 1.94421 | 1.924768 | 1.31296 | 1.29983 |
| 1404 | 0.95876 | 0.949172 | 1.22584 | 1.213582 |
| 1416 | 0.70862 | 0.701534 | 0.66339 | 0.656756 |
| 1428 | 0.99133 | 0.981417 | 0.56294 | 0.557311 |
| 1440 | 0.62418 | 0.617938 | 0.64438 | 0.637936 |
| 1452 | 2.09586 | 2.074901 | 0.74774 | 0.740263 |
| 1464 | 1.39424 | 1.380298 | 0.73911 | 0.731719 |
| 1476 | 1.29834 | 1.285357 | 0.70751 | 0.700435 |
| 1488 | 2.3771 | 2.353329 | 0.37496 | 0.37121 |
| 1500 | 2.66214 | 2.635519 | 0.43499 | 0.43064 |
| 1512 | 1.14503 | 1.13358 | 0.48009 | 0.475289 |
| 1524 | 1.03206 | 1.021739 | 0.77384 | 0.766102 |
| 1536 | 1.55316 | 1.537628 | 0.95876 | 0.949172 |
| 1548 | 2.09586 | 2.074901 | 1.11881 | 1.107622 |
| 1560 | 1.88885 | 1.869962 | 0.54341 | 0.537976 |
| 1572 | 1.23814 | 1.225759 | 0.60085 | 0.594842 |
| 1584 | 0.44995 | 0.445451 | 1.19597 | 1.18401 |
| 1596 | 0.83297 | 0.82464 | 1.39131 | 1.377397 |
| 1608 | 0.63486 | 0.628511 | 0.6054 | 0.599346 |
| 1620 | 0.88439 | 0.875546 | 0.59064 | 0.584734 |
| 1632 | 1.43894 | 1.424551 | 0.62151 | 0.615295 |
| 1644 | 2.48072 | 2.455913 | 1.23843 | 1.226046 |
| 1656 | 0.95403 | 0.94449 | 0.68029 | 0.673487 |
| 1668 | 0.53084 | 0.525532 | 0.58338 | 0.577546 |
| 1680 | 1.86773 | 1.849053 | 0.14845 | 0.146966 |
| 1692 | 2.77942 | 2.751626 | 1.18062 | 1.168814 |
| 1704 | 1.36528 | 1.351627 | 1.03627 | 1.025907 |
| 1716 | 0.76752 | 0.759845 | 0.58338 | 0.577546 |
| 1728 | 1.04032 | 1.029917 | 0.95876 | 0.949172 |
| 1740 | 2.74255 | 2.715125 | 0.48705 | 0.48218 |
| 1752 | 1.0909 | 1.079991 | 0.74086 | 0.733451 |
| 1764 | 0.9334 | 0.924066 | 1.47115 | 1.456439 |
| 1776 | 0.10423 | 0.103188 | 0.86218 | 0.853558 |
| 1788 | 2.04416 | 2.023718 | 0.74814 | 0.740659 |
| 1800 | 2.2331 | 2.210769 | 0.70005 | 0.69305 |
| 1812 | 0.9334 | 0.924066 | 0.57751 | 0.571735 |
| 1824 | 1.27888 | 1.266091 | 0.73324 | 0.725908 |


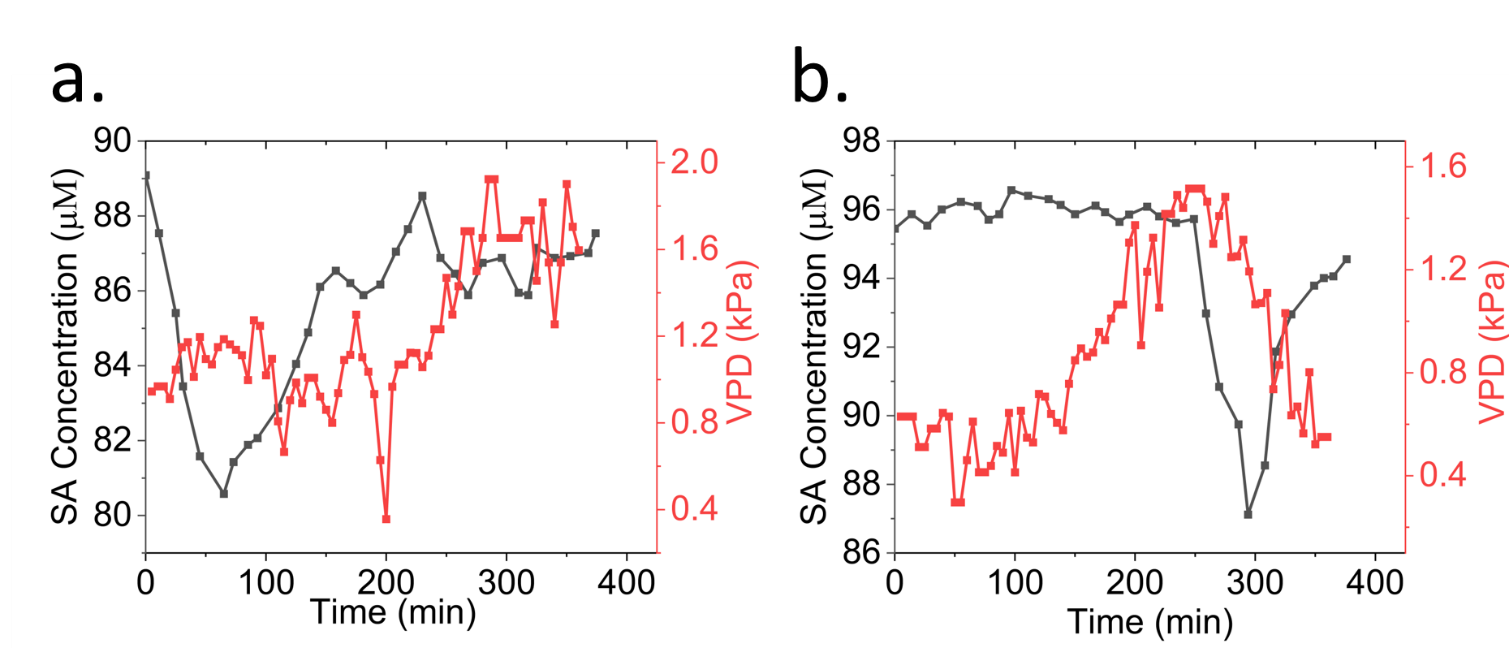


**Figure S19.** (a) A comparative analysis of the kinetics of SA and VPD at the (a) lower leaf (located at 40 cm from the soil surface) and (b) upper leaf (located at 105 cm from the soil surface) of a bell pepper plant.


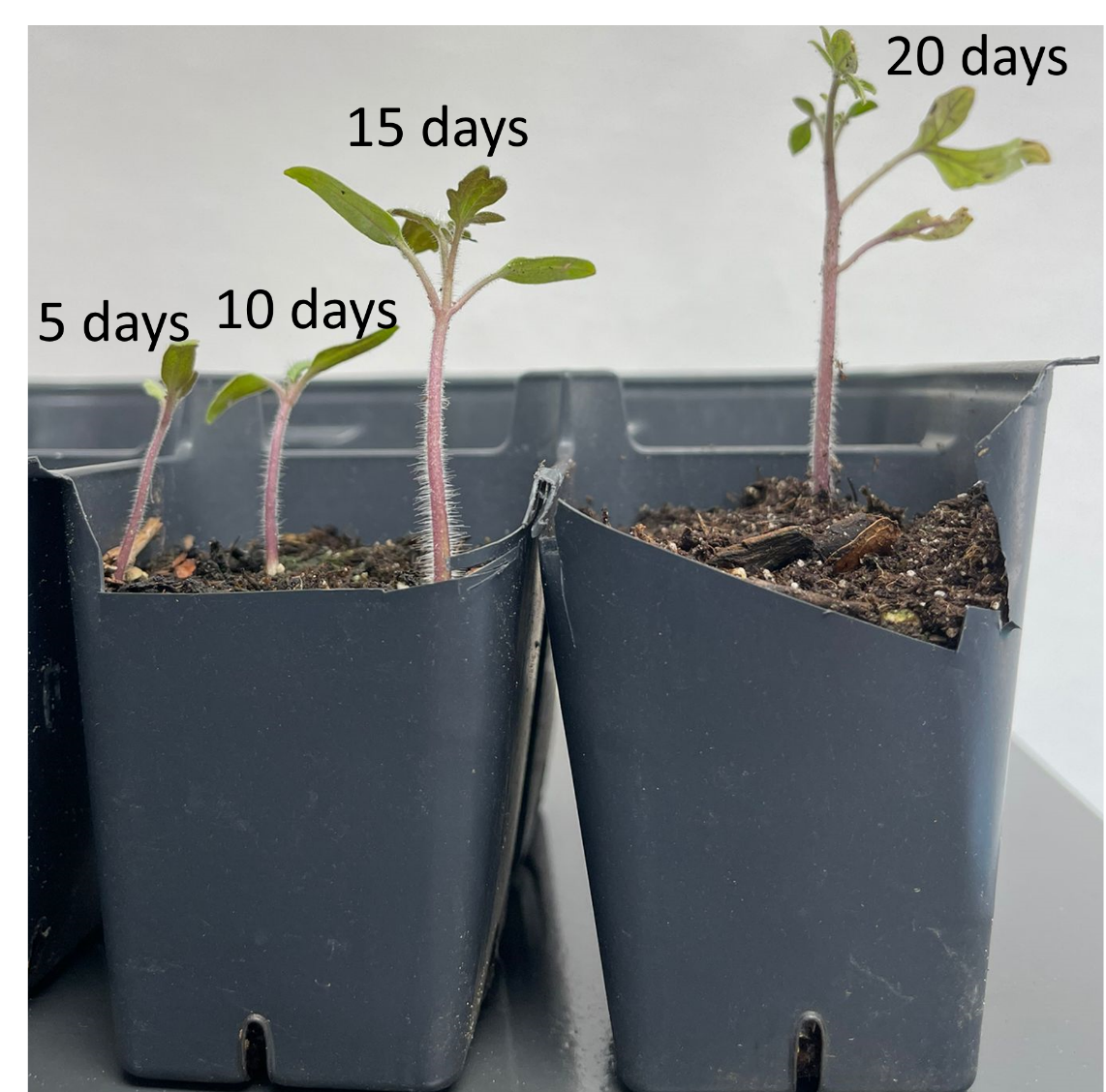


**Figure S20.**  Optical images of tomato plants at different stages of growth, i.e., 5, 10, 15, and 20 days after germination.


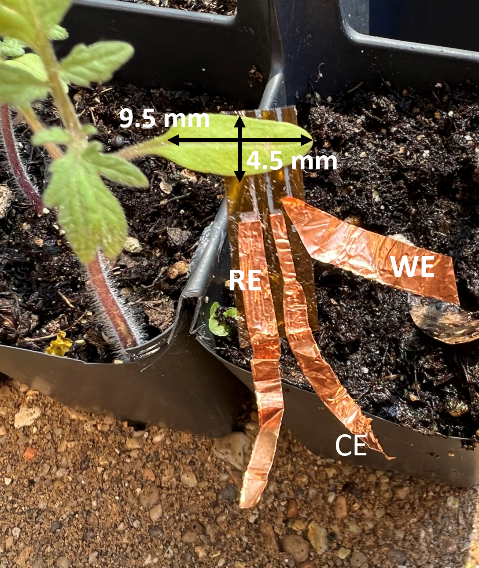


**Figure S21.** The reconfigured sensor suite was installed on the leaf of a 15-day-old tomato plant. The sensing regions of the combined SA, ethylene, temperature and humidity sensors fit into the leaf that was 9.5 mm long and 4.5 mm wide near the center.


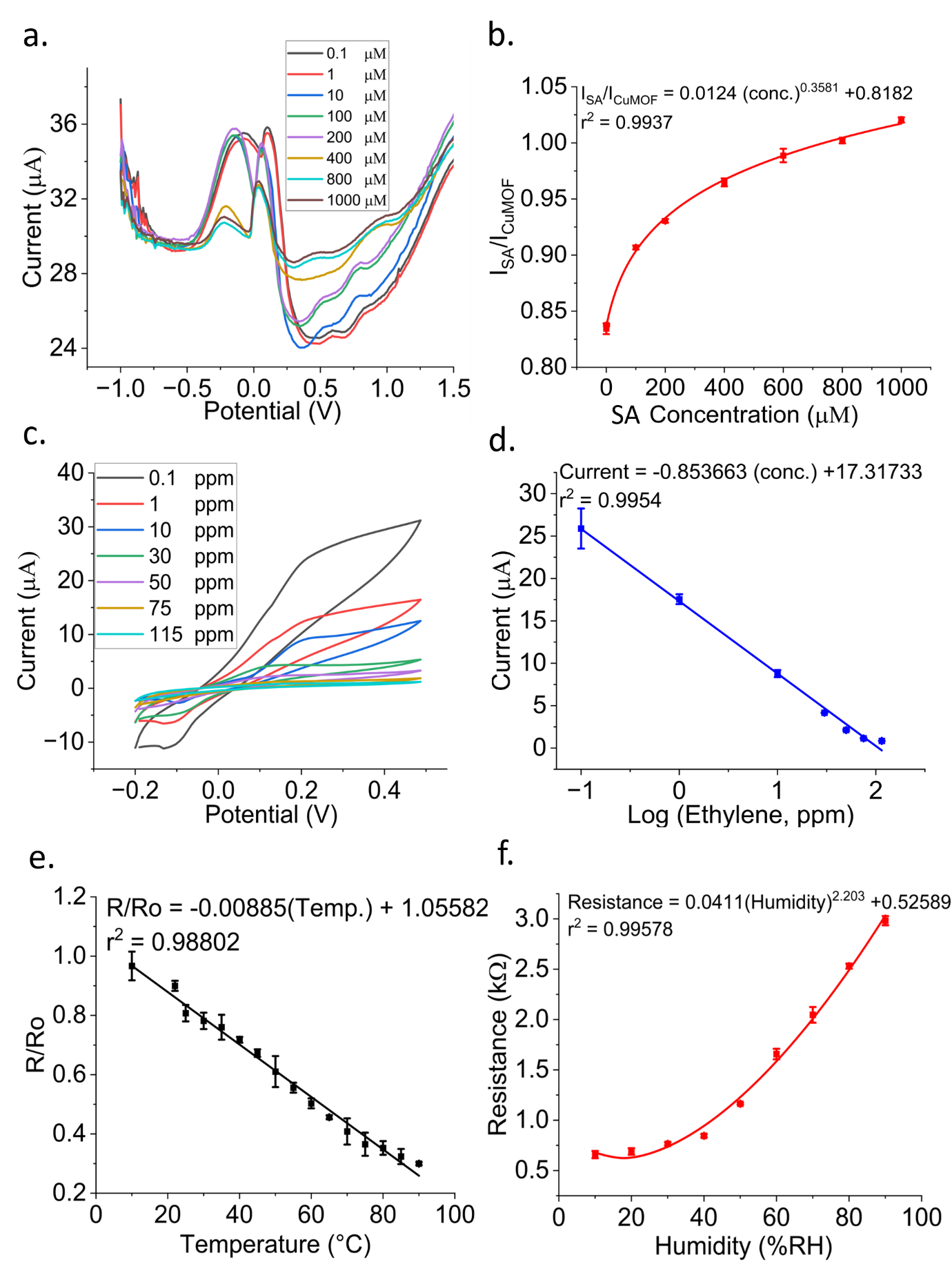


**Figure S22.**  Calibration plots of the modified sensor, as illustrated in Figure S15. (a) DPV responses of the SA sensor in response to varying concentrations of salicylic acid. (b) Calibration curve of the SA sensor indicating the plot of I_SA_/I_CuMOF_ against SA concentrations. (c) CV responses of the ethylene sensor for different concentrations of gaseous ethylene. (d) Calibration curve of ethylene sensor representing the peak current vs. logarithm of the ethylene concentration. Calibration curves for (e) temperature and (f) humidity sensors.


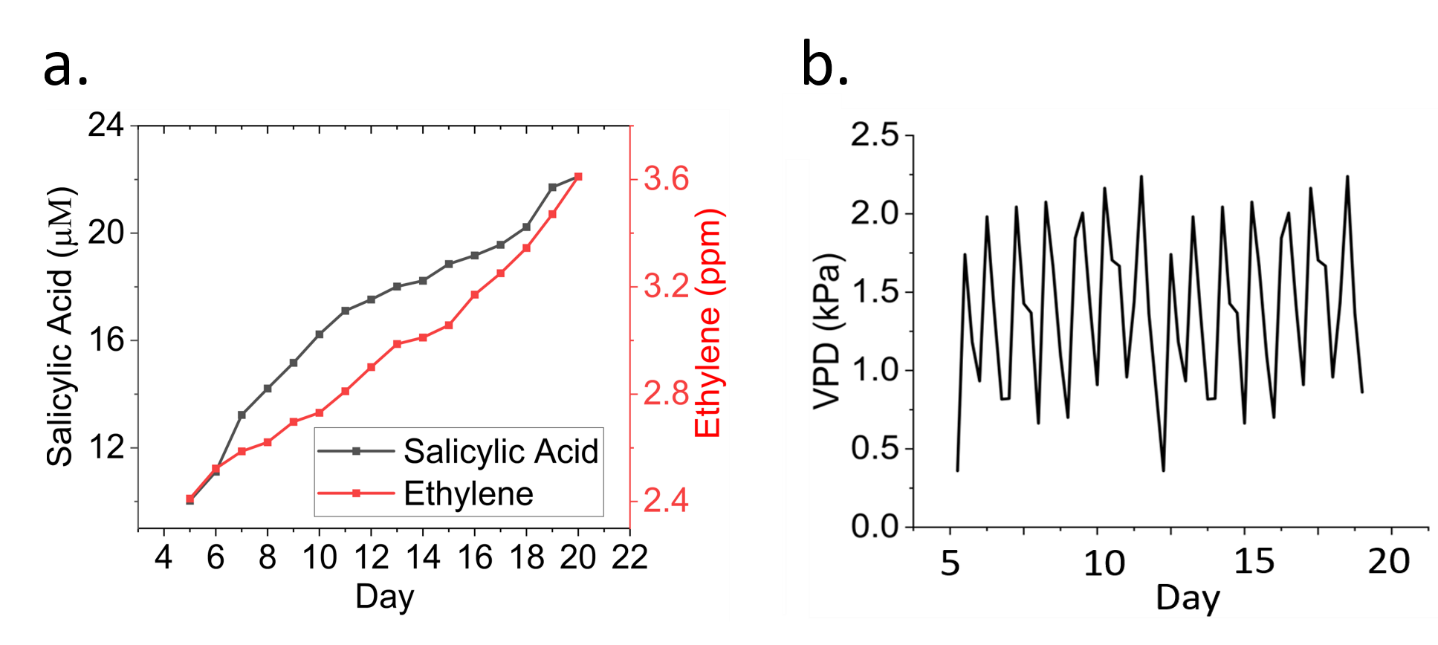


**Figure S23.** (a) Real-time SA and ethylene measurements and (b) Real-time VPD measurements from the tomato seedlings starting from day 5 to 20 of their growth period.


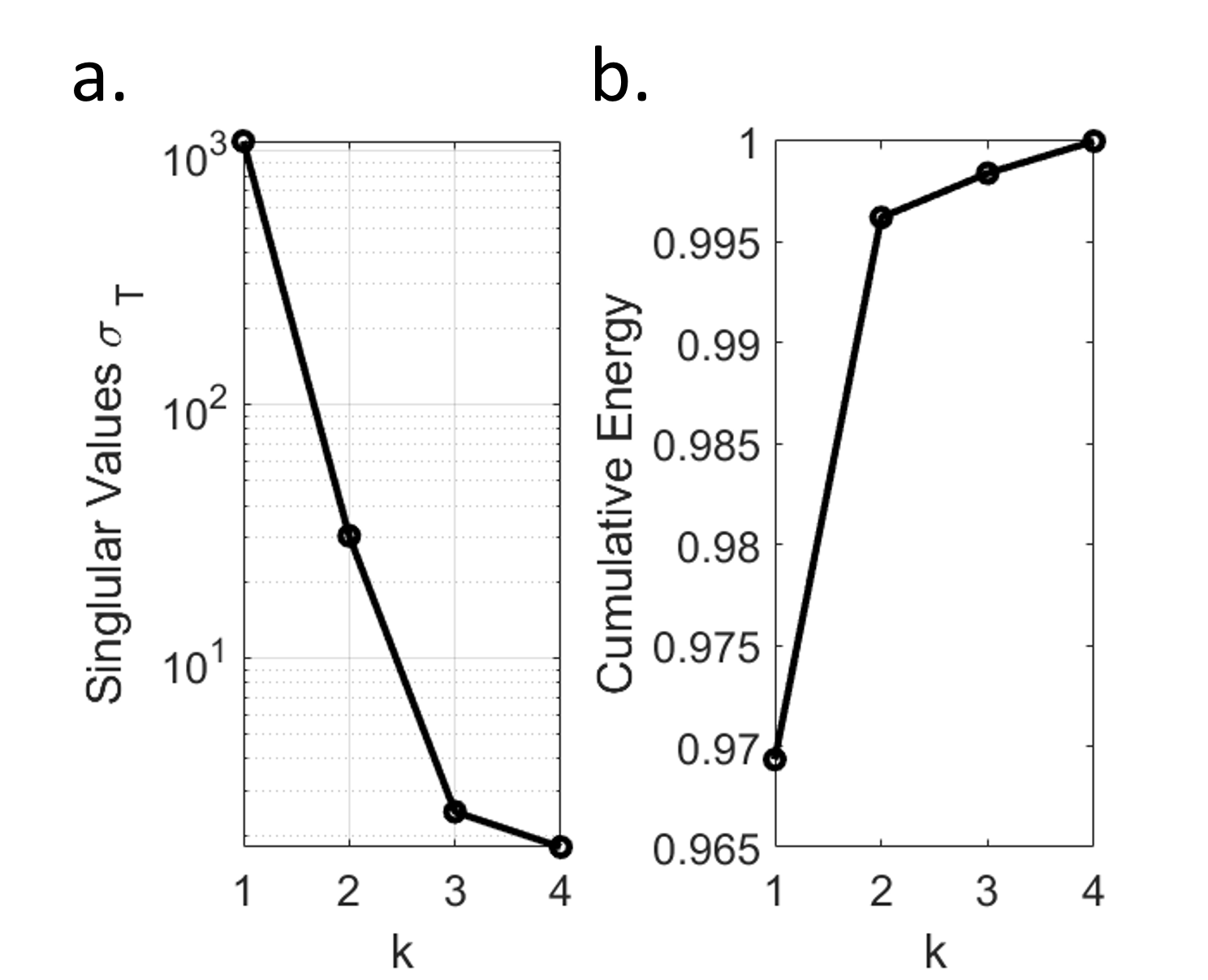


**Figure S24.** (a) Singular Value Deposition and (b) Cumulative Energy of different principal components. Here, k represents the principal component number. The first two principal components have larger singular values. In addition, the cumulative energy analysis demonstrates that the first principal component captures almost 97% of the total energy. These results confirm that the most significant features can be reliably extracted by the first two principal components.

**Code S1.** MATLAB code for autocorrelation analysis

clear all;

close all;

clc;

%% Begin data processing.

% Load data from the file.

signal= xlsread("data_Ethylene_orSA_40_plants",'Sheet1','A1:B40' );

% Weight of the curve

W=[1;1;1;1;1;1;1;1;1;1;1;1;1;1;1;1;1;1;1;1;1;1;1;1;1;1;1;1;1;1;1;1;1;1;1;1;1;1;1;1]

%% boxplot

boxplot(signal)

xlabel('Day')

ylabel('Cor. Coef. of ET Level')

xticks([1 10 20 30 40])

xticklabels({'1','10','20','30','40'});

**Code S2.** MATLAB code for Principle Component Analysis (PCA)

clear all

close all

clc

PD = xlsread('SA_Ethylene_VPD_.xlsx','A1:D40'); % load data

[U,S,V] = svd(PD); % Singular value Deposition

figure()

subplot(1,2,1)

semilogy((diag(S)),'k-o','LineWidth',2.5)% plot the singular values of each component

xlabel('k')

ylabel('Singlular Values \sigma _T')

set(gca),axis tight,grid on

subplot(1,2,2)

plot(cumsum(diag(S))./sum(diag(S)),'k-o','LineWidth',2.5)% plot cumulative energy of each component

xlabel('k')

ylabel('Cumulative Energy')

[coeff,score,latent,tsquared,expalined,mu]=pca(PD); Find the principle compont

pc1=score(:,1)% Get Principle component 1

pc2=score(:,2)% Get Principle component 2

S1 = xlsread('Book2.xlsx','A1:B10'); % load data(0-10 days of stress data or 0-5 days of growth data)

S2 = xlsread('Book2.xlsx','A11:B20'); % load data (11-20 days of stress data or 6-10 days of growth data)

S3 = xlsread('Book2.xlsx','A21:B30'); % load data (21-30 days of stress data or 11-15 days of growth data)

S4 = xlsread('Book2.xlsx','A31:B40'); % load data (31-40 days of stress data or 16-20 days of growth data)

S1_pc1=S1(:,1);% Sorting Principal Component 1 (0-10 days of stress data or 0-5 days of growth data)

S1_pc2=S1(:,2);% Sorting Principal Component 2 (0-10 days of stress data or 0-5 days of growth data)

S2_pc1= S2(:,1);% Sorting Principal Component 1 (11-20 days of stress data or 6-10 days of growth data)

S2_pc2=S2(:,2);% Sorting Principal Component 2 (11-20 days of stress data or 6-10 days of growth data)

S3_pc1=S3(:,1);% Sorting Principal Component 1 (21-30 days of stress data or 11-15 days of growth data)

S3_pc2=S3(:,2);% Sorting Principal Component 2 (21-30 days of stress data or 11-15 days of growth data)

S4_pc1=S4(:,1);% Sorting Principal Component 1 (31-40 days of stress data or 16-20 days of growth data)

S4_pc2=S4(:,2);% Sorting Principal Component 2 (31-40 days of stress data or 16-20 days of growth data)

figure()

plot(S1_pc1,S1_pc2,'rx','LineWidth',2);% plot 0-10 days of stress data or 0-5 days of growth data

hold on

plot(S2_pc1,S2_pc2,'bo','LineWidth',2);% plot 11-20 days of stress data or 6-10 days of growth data

hold on

plot(S3_pc1,S3_pc2,'go','LineWidth',2);% plot 21-30 days of stress data or 11-15 days of growth data

hold on

plot(S4_pc1,S4_pc2,'kx','LineWidth',2);% plot 31-40 days of stress data or 16-20 days of growth data

**Code S3.** MATLAB code for cross-correlation analysis

clear all

close all

clc

PD = xlsread('data_for_xcor_SA_ET_VPD.xlsx','A1:C40'); % load data

SA=PD(:,1); % Salicylic Acid data

ET=PD(:,2); % Ethylene data

VPD =PD(:,3);% VPD data

[c1,lags1] = xcorr(SA,ET,'normalized'); % Computing the cross correlation between SA and ET

[c2,lags2] = xcorr(SA,VPD,'normalized'); % Computing the cross correlation between SA and VPD

[c3,lags3] = xcorr(ET,VPD,'normalized'); % Computing the cross correlation between ET and VPD

figure(1)

stem(lags1,c1)% Cross-correlation coefficient plot of Salicylic acid and Ethylene

ylabel('X Corr. SA and ET')

xlabel('Lags')

figure(2)

stem(lags2,c2)% Cross-correlation coefficient plot of Salicylic acid and VPD

ylabel('X Corr. SA and VPD')

xlabel('Lags')

figure(3)

stem(lags3,c3)% Cross-correlation coefficient plot of Ethylene and VPD

ylabel('X Corr. ET and VPD')

xlabel('Lags')

**References**

# [S1] Poltarzewski, Z., Staiti, P., Alderucci, V., Wieczorek, W. & Giordano, N. Nafion Distribution in Gas Diffusion Electrodes for Solid‐Polymer‐Electrolyte‐Fuel‐Cell Applications. *J. Electrochem. Soc.* 139, 761 (1992).

# [S2] Dewulf, D. W. & Bard, A. J. The electrochemical reduction of CO_2_ to CH_4_ and C_2_H_4_ at Cu/Nafion electrodes (solid polymer electrolyte structures). *Catalysis Letters* 1, 73-79 (1988).

[S3] Hossain, N. I., Noushin, T. & Tabassum, S. Leaf-FIT: A wearable leaf sensor for in-situ and real-time monitoring of plant phytohormones. *IEEE Sensors*, Sydney, Australia, Oct. 31- Nov. 4, 1-4 (2021). DOI: 10.1109/SENSORS47087.2021.9639842.

[S4] Tabassum, S., Kumar, D. P. & Kumar, R. *IEEE Sens. J.* **21**, 17420-17429 (2021).

# [S5] Rahman, S., Mohamed, N. M. & Sufian, S. Effect of acid treatment on the multiwalled carbon nanotubes. *Mater. Res, Innovations* 18, 196-199 (2014).

# [S6] Osorio, A. G., Silveira, I. C. L., Bueno, V. L. & Bergmann, C. P. H_2_SO_4_/HNO_3_/HCl—Functionalization and its effect on dispersion of carbon nanotubes in aqueous media. *Appl. Surf. Sci.* 255, 2485-2489 (2008).

## [S7] Menon, H., Aiswarya, R. & Surendran, K. P. Screen printable MWCNT inks for printed electronics. *RSC Adv.* **7**, 44076-44081 (2017).

# [S8] Wang, W., Wang, J., Kang, Y. & Wang, A. Synthesis, swelling and responsive properties of a new composite hydrogel based on hydroxyethyl cellulose and medicinal stone. *Composites: Part B Engineering* 42, 809-818 (2011).

[S9] Turkani, V. S., Maddipatla, D., Narakathu, B. B., Saeed, T. S., Obare, S. O., Bazuin, B. J. & Atashbar, M. Z. [A highly sensitive printed humidity sensor based on a functionalized MWCNT/HEC composite for flexible electronics application](https://pubs.rsc.org/en/content/articlehtml/2019/na/c9na00179d). *Nanoscale Adv.* **1**, 2311-22 (2019).

# [S10] Noushin, T., Hossain, N. I. & Tabassum, S. Kirigami-patterned highly stable and strain insensitive sweat pH and temperature sensors for long-term wearable applications. [*IEEE Healthcare Innovations and Point of Care Technologies (HI-POCT)*](https://ieeexplore.ieee.org/xpl/conhome/9744017/proceeding), Houston, USA, March 10-11, 1-4 (2021). DOI: [10.1109/HI-POCT54491.2022.9744070](https://doi.org/10.1109/HI-POCT54491.2022.9744070).

# [S11] More, C. V., Alsayed, Z., Badawi, M. S., Thabet, A. A. & Pawar, P. P. Polymeric composite materials for radiation shielding: a review. *Environ. Chem. Lett.* 19, 2057–2090 (2021).

[S12] Kim, C. B., Jeong, K. B., Yang, B. J., Song, J. -W., Ku, B. -C., Lee, S., Lee, S. -K. & Park, C. [Facile supramolecular processing of carbon nanotubes and polymers for electromechanical sensors](https://onlinelibrary.wiley.com/doi/abs/10.1002/ange.201708111). *Angew. Chem. Int. Ed.* **56**, 16180-16185 (2017).

[S13] Wang, Y. -F., Sekine, T., Takeda, Y., Hong, J., Yoshida, A., Kumaki, D., Shiba, T. & Tokito, S. Deep eutectic solvent induced porous conductive composite for fully printed piezoresistive pressure sensor. *Adv. Mater. Technol.* **6**, 2100731 (2021).

[S14] Hossain, N. I. & Tabassum, S. An internet-of-things enabled flexible strain sensor for stem growth measurements. *IEEE Sensors*, Sydney, Australia, Oct. 31- Nov. 4, 1-4 (2021). DOI: 10.1109/SENSORS47087.2021.9639472.

# [S15] Khan, M. R. R. & Kang, S. -W. Highly Sensitive Multi-Channel IDC Sensor Array for Low Concentration Taste Detection. *Sensors* 15, 13201-13221 (2015).

# [S16] Wang, S., Deng, W., Yang, L., Tan, Y., Xie, Q. & Yao, S. Copper-Based Metal-Organic Framework Nanoparticles with Peroxidase-Like Activity for Sensitive Colorimetric Detection of Staphylococcus aureus. *ACS Appl. Mater. Interfaces* 9, 24440–24445 (2017).

[S17] Noushin, T., Hossain, N. I. & Tabassum, S. [IoT-enabled integrated smart wound sensor for multiplexed monitoring of inflammatory biomarkers at the wound site](https://www.frontiersin.org/articles/10.3389/fnano.2022.851041/full). *Front. Nanotechnol.* **4**, 851041 (2022).

# [S18] Zhao, M., Hibbert, D. B. & Gooding, J. J. Solution to the problem of interferences in electrochemical sensors using the fill-and-flow channel biosensor. *Anal. Chem.* 75, 593–600 (2003).

## [S19] Yang, L., Chen, D., Wang, X., Luo, B., Wang, C., Gao, G., Li, H., Li, A. & Chen, L. Ratiometric electrochemical sensor for accurate detection of salicylic acid in leaves of living plants. *RSC Adv.* **10**, 38841-46 (2020).
